# An international survey and modified Delphi process revealed editors’ perceptions, training needs, and ratings of competency-related statements for the development of core competencies for scientific editors of biomedical journals

**DOI:** 10.12688/f1000research.12400.1

**Published:** 2017-09-04

**Authors:** James Galipeau, Kelly D. Cobey, Virginia Barbour, Patricia Baskin, Sally Bell-Syer, Jonathan Deeks, Paul Garner, Larissa Shamseer, Straus Sharon, Peter Tugwell, Margaret Winker, David Moher

**Affiliations:** 1 Centre for Journalology, Clinical Epidemiology Program, Ottawa Hospital Research Institute, Ottawa, Canada; 2 Department of Psychology , University of Stirling, Stirling, UK; 3 School of Epidemiology, Public Health and Preventive Medicine, Faculty of Medicine , University of Ottawa, Ottawa, Canada; 4 Office of Research Ethics and Integrity, Division of Research and Commercialisation and Library, Division of Technology, Information and Library Services QUT, Brisbane, Australia; 5 Council of Science Editors , Denver , Colorado, USA; 6 American Academy of Neurology , St. Paul , Minnesota, USA; 7 Cochrane Central Executive , St Albans House, London, UK; 8 Department of Health Sciences , University of York, York, UK; 9 Institute of Applied Health Research , College of Medical and Dental Sciences , University of Birmingham , Birmingham, UK; 10 Department of Clinical Sciences , Liverpool School of Tropical Medicine , Liverpool, UK; 11 Department of Medicine , University of Toronto , Toronto, Canada; 12 Department of Medicine , Faculty of Medicine , University of Ottawa , Ottawa, Canada; 13 World Association of Medical Editors , Greater Chicago Area, Chicago, USA

**Keywords:** scientific editor, core competencies, biomedical, journal, training needs, Delphi

## Abstract

**Background:** Scientific editors (i.e., those who make decisions on the content and policies of a journal) have a central role in the editorial process at biomedical journals. However, very little is known about the training needs of these editors or what competencies are required to perform effectively in this role.

**Methods:** We conducted a survey of perceptions and training needs among scientific editors from major editorial organizations around the world, followed by a modified Delphi process in which we invited the same scientific editors to rate the importance of competency-related statements obtained from a previous scoping review.

**Results:** A total of 148 participants completed the survey of perceptions and training needs. At least 80% of participants agreed on six of the 38 skill and expertise-related statements presented to them as being important or very important to their role as scientific editors. At least 80% agreed on three of the 38 statements as necessary skills they perceived themselves as possessing (well or very well).  The top five items on participants’ list of top training needs were training in statistics, research methods, publication ethics, recruiting and dealing with peer reviewers, and indexing of journals. The three rounds of the Delphi were completed by 83, 83, and 73 participants, respectively, which ultimately produced a list of 23 “highly rated” competency-related statements and another 86 “included” items.

**Conclusion: **Both the survey and the modified Delphi process will be critical for understanding knowledge and training gaps among scientific editors when designing curriculum around core competencies in the future.

## Background

The Declaration of Helsinki (2013) asks editors to ensure that the quality of what they publish is of the highest quality possible ^1^. However, very little is known about the training needs of scientific editors (i.e., those who make decisions on the content and policies of a journal) or what competencies are required to meet these standards. Presently, a large portion of scientific editors’ learning is informal, often learned on the job through mentoring ^2^. While formal training opportunities for scientific editors do exist in the form of fellowships and intensive courses, these opportunities are limited to a very small number of editors annually. In addition, there may be variations across training opportunities, which are not evidence-based, owing to the lack of any consensus or evidence on the knowledge, skills, and abilities that editors should possess to be competent in their job.

While there is no shortage of published literature on the role of scientific editors, most of this takes the form of opinion-based editorials; very few recommendations are evidence-based. For example, a 2015 systematic review of training in writing for scholarly publication, journal editing, and peer review found no studies of formalized training programs for journal editors ^3^. While a 2016 scoping review from our group ^4^ found 25 research-based publications relating to scientific editors, the majority were surveys on a wide variety of topics relating to scientific editors. The same study found 136 published articles, of which 133 were non-research based editorials relating to various aspects of scientific editorship. An associated environmental scan also found an additional 35 documents that were not published in scientific journals, of which 18 were produced by journals, while nine were from associations and societies, six from organizations providing guidance to editors, and two from publishers.

In 1999, the World Association of Medical Editors conducted a global survey of journal editors regarding the characteristics of their respective journals. This survey included one item related to scientific editor training: of the 269 respondents, 75% said they wanted training for newly appointed editors. Other surveys have echoed this opinion as well ^2, 5, 6^; however to our knowledge, there have been no dedicated attempts to formally assess the training needs of scientific editors of academic journals. We are also unaware of any large scale collaborative effort to determine the competencies that are required for the role of scientific editor. There are a number of examples of editorial organizations, publishers, and individuals ^7^ who have put forth their opinions on what makes a good scientific editor, yet there lacks clarity on how these ideas were derived, how they relate to each other, or whether they are universal. Additionally, the perceptions and training needs of scientific editors are not well represented in the literature.

The objective of this research was to better understand the training needs and perceptions of competence of scientific editors of journals. We also sought to solicit editors’ opinions on the importance of particular knowledge, skills, abilities, and characteristics to carrying out their editorial duties.

## Methods

The research presented here is the second step of a larger program that our team is carrying out to develop a universal, minimum set of core competencies for scientific editors of biomedical journals. In our first project, we carried out a scoping review of the published research literature and an environmental scan of non-research-based materials to identify competency-related statements found online and in previous research ^4^. In the current project described here, we aimed to solicit the perspective of editors worldwide and have them narrow and refine the number of potential core competencies related to their position. In the third and final step of the process, we brought international experts together to decide on a final set of core competencies, publication of which is forthcoming.

The current research comprised a survey of scientific editors to understand their perceptions of their role as an editor and to identify any training needs, followed by a modified Delphi process whereby editors rated the importance of 200+ competency-related statements obtained from the aforementioned previous scoping review (describing knowledge, skills, and abilities associated with the role of scientific editor) ^4^. Surveying to gauge training needs and to gather consensus are common tools for creating a competency-based core curriculum in the biomedical field ^8– 12^. The study was approved by the Ottawa Health Science Network Research Ethics Board.

### Participants

We approached current or former scientific editors of journals, defined as editors who make decisions on the content and policies of a journal – including editors-in-chief and associate/academic editors. Recruitment advertisements were sent to editorial organizations and groups having a large scientific editor membership from around the world. These organizations forwarded the advertisement about the survey of editors to their members through a distribution list email or an announcement on a listserv or message board. The organizations are listed in Box 1:

**Box 1. Organizations forwarding the advertisement about the survey of editors to their members**

**Table:** 

Organization	Website
Cochrane (formerly The Cochrane Collaboration)	http://www.cochrane.org
Council of Science Editors (CSE)	https://www.councilscienceeditors.org
Committee on Publication Ethics (COPE)	http://publicationethics.org
Eastern Mediterranean Association of Medical Editors (EMAME)	http://www.emro.who.int/entity/emame
European Association of Science Editors (EASE)	http://www.ease.org.uk
PLoS One (A journal of the Public Library of Science)	http://journals.plos.org/plosone
World Association of Medical Editors (WAME)	http://www.wame.org

### Phase 1: Survey of Editor Perceptions and Training Needs

We developed an online-based assessment of editors’ perceptions and training needs that was anonymous and self-administered ( Supplementary File S1). The questionnaire was developed based on data collected in our previous scoping review 4, as well as with input from our research team, comprising scientific editors, representatives from publishing houses, educational experts, and specialists in publication science. Questions were designed to broadly cover major areas associated with the scientific editor role, including editors’ knowledge, expertise, skills, and experience. The questionnaire was not validated; however, it was piloted among five experienced scientific editors of biomedical journals and subsequently revised based on their feedback. The revised questionnaire was uploaded to SurveyMonkey ( www.surveymonkey.com) and the survey URL was sent to the editorial organizations for distribution.

The survey contained 19 demographic questions relating to participants’ age, sex, education level, job title, editorial experience, the journal they edit, and their editorial training experience. The perceptions of respondents were also examined in four areas: Participants indicated in the first two instances how important they thought a series of competency-related statements was to the scientific editor role, and, in the latter two instances, how much they thought they possessed these same competencies. Response options on a 1–7 Likert scale were provided (as indicated):

1. The degree to which participants perceive that expertise-related items are important to their job as editor (18 items) (Response options: Not Important to Very Important)
2. The degree to which participants perceive that particular skills and experience are important to their job as editor (20 items) (Response options: Not Important to Very Important)
3. The degree to which participants perceive they possess particular expertise-related items related to their job as editor (same 18 items as #1) (Response options: Not Much to Very Much)
4. The degree to which participants perceive they perform particular skills and possess particular experience related to their job as editor (same 20 items as #2) (Not Well/Not Much to Very Well/Very Much)

Finally, participants were asked to create a ranked list of their top 10 training needs (from #1 being the most important to #10 being least important). Participants were also asked if they would be willing to participate in a Delphi process to rate the importance of a much larger and more detailed list of competency-related statements for scientific editors of biomedical journals. If so, they were asked to provide their email addresses and were included in the list of potential participants for the modified Delphi process.

### Phase 2: Modified Delphi Process

We carried out a three-round modified Delphi process in order to rate and refine the list of potential competencies derived in a previous scoping review we conducted ^4^. A Delphi process typically involves experts and takes place in the form of iterations or “rounds” (normally 2 to 4) in which data is collected anonymously, often on-line, and then fed back to the group in an aggregated and de-identified way, along with individual participants’ comments ^13^. In the current project, we modified the Delphi in several ways: First, we solicited the involvement of any scientific editors of biomedical journals, not only experts, as is normally the case with a Delphi process. Next, we did not require all participants to be involved in all rounds of the modified Delphi. In addition, the number of items included in the modified Delphi was much greater than a Delphi would generally include. Finally, for the sake of efficiency (due to the large number of items), in the third round we did not ask participants to re-rate items that had reached consensus for inclusion or exclusion in Round 2.

Interested Phase 1 participants were invited to participate in the online modified Delphi, which was also administered via SurveyMonkey. For each Delphi round, an invitation was sent to the entire list of potential participants, regardless of whether they responded in the previous round.

**Round 1.** Participants were asked to rate each competency-related statement on a 5-point Likert scale, from 1 (Not at all Important) to 5 (Absolutely Essential). At the end of each section, an open text box was provided for participants to include comments relating to items in that section if desired. Participants were also asked to name any potential competencies that were not included in the Delphi. Participants were reminded that all competency-related statements exclusively related to the position of Editor-in-Chief had been intentionally removed from the Delphi, as was the case for the scoping review on which the Delphi was built.

**Round 2.** Phase 1 participants were invited to participate in Round 2 of the Delphi, regardless of whether they completed Round 1. Along with the email invitation, they were provided the mean score for each of the items from Round 1, the participant’s own score for each of the items, if relevant, and collated (de-identified) comments from the text boxes. Participants were then asked to re-rate and provide their rationale for disagreement only for those items for which they disagreed with the mean score from Round 1. Any new competency-related statements arising from the final question in Round 1 were included for rating in the Round 2 survey. Participants were asked to rate these new items in the same way that items had been rated in Round 1, that is, on a 5-point Likert scale from 1 (Not at all Important) to 5 (Absolutely Essential).

**Round 3.** Phase 1 participants were invited by email to participate in Round 3 of the Delphi, regardless of whether they completed Round 1 and/or Round 2. Attached to the e-mail was a document listing the Round 1 and Round 2 average scores for all items and participants’ comments from previous rounds. Items that did not reach consensus for inclusion or exclusion in Round 2 were highlighted in yellow. Participants were asked to re-rate these highlighted items in Round 3 using a 3-point Likert scale from 1 (Less Important) to 3 (Essential). The shift to a 3-point Likert scale was aimed at simplifying the process for participants by limiting the number of options to the manner in which the ratings of each competency statement would be analysed (i.e., consensus on a ‘1’ would mean the item was excluded and consensus on a 3 would mean the item was included).

### Analysis

To establish inclusion and exclusion criteria for both the survey of editors and the modified Delphi, we pre-specified the consensus level at 80% of respondents. This decision was based on the use of an 80% cut-off rate in previous Delphi studies in healthcare ^14^ and education ^15^.

The data on the 76 perception-related survey questions were summarized by calculating mean scores for each of the questions. These scores were then classified as reaching consensus for inclusion (≥6 on a 7-point Likert scale) or exclusion (≤3 on a 7-point Likert scale), for use in a future consensus meeting. The ranked top 10 lists of training needs were collated by one author (JG) by regrouping similar statements and these groupings were then verified by another author (KDC).

In Round 1 of the Delphi, means were calculated for each of the items and consensus for inclusion (≥4 on a 5-point Likert scale) or exclusion (≤2 on a 5-point Likert scale) was determined. In Round 2, means were calculated for each of the items from Round 1 and consensus for inclusion and exclusion was updated. For any new items suggested by participants in Round 1, mean scores were calculated and consensus for inclusion and exclusion was determined in Round 2. In Round 3, means were calculated for items that had not reached consensus for inclusion or exclusion in Round 2. Final consensus for inclusion was set at 80% of participants selecting a ‘3’ on a 3-point Likert scale, while exclusion was set at 80% selecting a ‘1’ on the scale. A final list of included and excluded items was created for use in a future consensus meeting. Due to the large volume of included items in the final list, a post-hoc decision was made to create a shorter list of “highly rated” items that reached 90% consensus for inclusion.

### Ethical considerations

All participants provided consent through an online form preceding the survey of editors and Delphi. In order to stimulate participation and thank participants, an iPad Mini was awarded as a draw prize after the survey of editors and after each round of the Delphi. Participants who wished to be entered into the draw were asked to provide their email address. These email addresses were used for the sole purpose of the draw and removed from the data prior to analysis. All data from this research was pooled for presentation in the results section.

## Results

**Figure 1.**
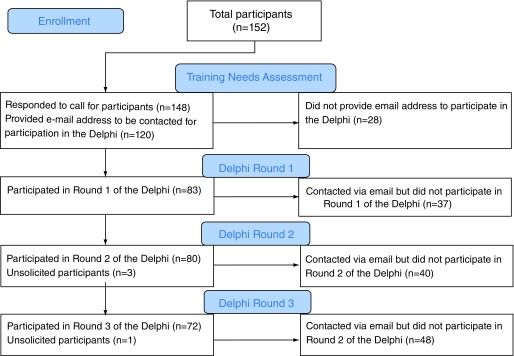
Participant Flow – flow of participants completing the needs assessment and round 1, 2, and 3 of the Delphi exercise.

A total of 152 participants completed either the survey of editors’ perceptions and assessment of their training needs or the Delphi process ( Figure 1). As described in Figure 1, four respondents did not complete the survey of editors but completed one round each of the Delphi and their responses were included in the analysis. We presume these four respondents were invited by other participants as access was not restricted for completing the Delphi.

### Editors’ Perceptions and Ranking of their Training Needs

***Demographic data.*** A total of 148 editors from around the world accessed and completed the survey of their perceptions ( Table 1 & Supplementary File S2). Respondents were mainly those replying to the notices for participation posted by the World Association of Medical Editors (WAME) and Cochrane. Nearly 2/3 of respondents were male. Close to 2/3 had more than seven years of experience as an editor. The majority of editors indicated that their journal had provided formal or informal training related to their job and that they had sought both formal and informal training beyond what was provided by their employer. The large majority indicated having formal or informal training in research methods and statistics.

**Table 1.** Participant characteristics – demographic data of editors and journals.

EDITOR CHARACTERISTICS		N (%)
Gender (n=150)	Male	88 (58.67)
	Female	61 (40.67)
	Prefer not to answer	1 (0.67)
What is your age? (n=150) • 18–24 • 25-34 • 35–44 • 45–54 • 55–64 • 65–74 • Older than 74 • Prefer not to answer		2 (1.33) 9 (6.00) 36 (24.00) 44 (29.33) 36 (24.00) 16 (10.67) 4 (2.67) 3 (2.00)
Education * (n=146)	Bachelor Degree	10 (6.85)
	Master Degree	33 (22.60)
	Ph.D.	75 (51.37)
	M.D. or equivalent	54 (36.99)
Referring organization (n=150)	COPE	3 (2.00)
	WAME	53 (35.33)
	CSE	2 (1.33)
	Cochrane	49 (32.67)
	EASE	20 (13.33)
	PLOS One	12 (8.00)
	Other (EMAME, Twitter, OHRI, friend)	11 (7.33)
Journal Role (n=148)	Editor-in-Chief	51 (34.46)
	Associate Editor	34 (22.97)
	Academic Editor	7 (4.73)
	Other (e.g., Editor, Section Editor, Deputy Editor, Senior Editor)	56 (37.84)
Primary professional role outside of journal (n=150)	My scientific editorship is my main employment	28 (18.12)
	Other (e.g., Professor, Researcher, Research Fellow, Senior Lecturer)	122 (81.88)
Number of years of experience as editor) (n=150)	• Less than a year • 1–3 years • 4–6 years • 7–10 years • 11–15 years • 16–20 years • 20+ years	5 (3.33) 23 (15.33) (23.33) 29 (19.33) 24 (16.00) 17 (11.33) 17 (11.33)
Are you a member of any of the following editorial organizations * (Check all that apply): (n=88)	World Association of Medical Editors (WAME)	57
	Committee on Publication Ethics (COPE)	37
	Council of Science Editors (CSE)	15
	European Association of Science Editors (EASE)	21
	Forum of African Medical Editors (FAME)	2
	Asia Pacific Association of Medical Journal Editors (APAME)	10
	Other (e.g., EMAME, ICMJE, None)	30
Received formal training as a scientific editor (e.g., workshops, courses, formal mentoring) beyond or before what was provided at your current journal? (n=147)	Yes No	94 (63.95) 53 (36.05)
Received informal training as scientific editor (e.g., books, websites, informal mentoring) beyond or before what was provided at your current journal? (n=145)	Yes No	81 (55.86) 64 (44.14)
Trained (formal or informal) in research methods (n=148)	Yes No	133 (89.86) 15 (10.14)
Trained (formal or informal) in statistics (n=148)	Yes No	122 (82.43) 26 (17.57)
**JOURNAL CHARACTERISTICS**		**N (%)**
Type of journal (n=149)	General Journal	49 (32.89)
	Specialty Journal	77 (51.68)
	Other (e.g., Cochrane, Cochrane Library)	23 (15.44)
Journal or publisher Location (n=148)	North America	41 (27.70)
	South America or Central America	3 (2.03)
	Europe	63 (42.57)
	Asia	26 (17.57)
	Africa	7 (4.73)
	Australia	8 (5.41)
Intended audience (n=150)	National	8 (5.33)
	International	138 (92.00)
	Other (e.g., both, global, international but with emphasis on a region)	4 (2.67)
Number of publications in 2015 (n=145)	1–10	8 (5.52)
	11–30	23 (15.86)
	31–50	33 (22.76)
	51–100	17 (11.72)
	More than 100	64 (44.14)
Committee on Publication Ethics (COPE) member (n=145)	Yes	82 (56.55)
	No	29 (20.00)
	Unsure	34 (23.45)
Training (formal or informal) offered by journal (n=148)	Yes No	86 (58.11) 62 (41.89)

*This question allowed participants to enter multiple responses

***Editor Perceptions.* The degree to which participants perceive that expertise-related items are important to their job as editor.** Of the 18 questions related to participants’ perceptions of the importance of particular expertise-related items to their job as editor, two items reached consensus for inclusion ( Table 2). The highest consensus was for ‘expertise in research methods’ (85.4%), followed by ‘expertise in dealing with publication ethics, including conflicts of interest of authors, reviewers, and editors and the editorial board’ (80.4%). None of the items reached consensus for exclusion.

**Table 2.** Perceived importance of, and degree to which scientific editors thought they possess, particular expertise and skills related to their editorial role.

Item	% Rating the Perceived Importance of Expertise Highly ^1^	% Rating the Perceived Possession of Expertise Highly ^2^
Expertise in research methods	85.4% *	72.1%
Expertise in dealing with publication ethics including conflicts of interest of authors, reviewers, and editors and the editorial board	80.4% *	70.6%
Expertise in dealing with research misconduct (falsification, fraud, plagiarism, duplicate publication); how to deal with allegations of misconduct; retraction	76.2%	69.9%
Expertise in the subject areas in which your journal publishes	72.7%	60.7%
Expertise in dealing with authorship issues	69.5%	60.3%
Expertise related to the roles and responsibilities within a journal	69.2%	58.8%
Expertise in the publication process (decision-making aspects) for research papers, commentary, and correspondence	67.3%	55.9%
Expertise in dealing with human and animal ethical concerns, patient protection and confidentiality, data deposition issues and confidentiality	66.4%	54.4%
Expertise with figures and tables, including evaluation and appropriate construction	54.1%	51.1%
Expertise in statistics	52.4%	47.4%
Expertise in understanding the general journal publishing landscape and publishing business models, open access mandates	49.2%	39.0%
Expertise in understanding copyright/CC-BY (Creative Commons)	46.4%	36.8%
Expertise with supplemental material, including evaluation and appropriate selection	44%	36.6%
Expertise with journal indexing and how to get a journal indexed	44%	36.0%
Expertise in evaluating journal and article impact technologies related to publication (e.g., metrics)	43%	33.8%
Expertise in post-publication peer review	39.8%	31.9%
Expertise with the role of social media for journals	38.1%	28.8%
Expertise in the article production process (i.e., technical aspects) for online and/or print	36.1%	24.3%
**Item**	**% Rating the Perceived** **Importance of Skills Highly ^3^**	**% Rating the Perceived** **Possession of Skills Highly ^4^**
Behaving with integrity/professionalism	94.4% *	90.2% *
Using good judgment in decision-making	93.7% *	87.5% *
Language/writing skills	90.1% *	81.5% *
Author and peer reviewer correspondence; how to evaluate peer reviews, draft a revision letter and evaluate an author response letter and revision	86.6% *	77.9%
Skills in guidance and supervision	77.1%	72.6%
Interactions/maintaining a working relationship with staff at your journal	75.5%	71.9%
Identifying, evaluating, and rewarding peer reviewers	75.5%	64.4%
Assessing how well the needs and interests of your journal’s readership are being met	68.5%	51.5%
Interactions/maintaining a working relationship with the publisher of your journal and understanding and maintaining editorial freedom	57.9%	51.1%
Managerial skills	57.7%	51.1%
Working with, training, and supervising other editors at your journal	57.3%	48.5%
How to select and appoint an editorial board at your journal and understanding the pros and cons of the different models of editorial boards	55.6%	46.3%
Interactions/maintaining a working relationship with the journal’s owner and understanding and maintaining editorial freedom	55.1%	42.2%
Previous experience in scientific editing of a journal	54.5%	41.0%
Increasing manuscript submissions to your journal	52.8%	37.9%
Journal promotion/public relations skills	40.8%	29.8%
Interactions/maintaining a working relationship with the general public	35.2%	25.8%
Interactions/maintaining a working relationship with the third party company that manages your journal’s submission management system	34.3%	23.0%
Writing news releases and maintaining relationships with the news media	31.0%	23.0%
Business skills	29.6%	23.0%

* = Reached 80% consensus of ≥6 on a 7-point Likert scale with endpoints of Not Important/Not Much/Not Well and Very Important/Very Much/Very Well ^**1**^Question asked: “Please rate THE IMPORTANCE of the following expertise-related items to the performance of your job as editor”; % indicates the percentage of respondents endorsing ≥6 on a 7-point Likert scale with endpoints of Not Important and Very Important ^**2**^Question asked: “Please rate THE IMPORTANCE of the following skills and experience to the performance of your job as editor”; % indicates the percentage of respondents endorsing ≥6 on a 7-point Likert scale with endpoints of Not Important and Very Important ^**3**^Question asked: “Please rate HOW MUCH YOU POSSESS the following expertise in your job as editor”; % indicates the percentage of respondents endorsing ≥6 on a 7-point Likert scale with endpoints of Not Much and Very Much ^**4**^Question asked: “Please rate HOW WELL YOU PERFORM the following skills or HOW MUCH YOU POSSESS the following experience in your job as editor”; % indicates the percentage of respondents endorsing ≥6 on a 7-point Likert scale with endpoints of Not Well and Very Well

**The degree to which participants perceive that particular skills and experience are important to their job as editor.** Of the 20 questions related to perceptions of the importance of particular skills and experience related to their job as editor, four items reached consensus for inclusion ( Table 2). The highest consensus was for ‘behaving with integrity/professionalism’ (94.4%), followed by ‘using good judgment in decision-making’ (93.7%), ‘language/writing skills’ (90.1%), and ‘author and peer reviewer correspondence; how to evaluate peer reviews, draft a revision letter and evaluate an author response letter and revision’ (86.6%). No items reached consensus for exclusion.

**The degree to which participants perceive they possess particular expertise-related items related to their job as editor.** Of the 18 questions related to participants’ perceptions of how much they possess particular expertise related to their job as editor, no items reached consensus for inclusion ( Table 2). The highest rated item was ‘expertise in research methods’ (72.1%), followed by ‘expertise in the publication process (decision-making aspects) for research papers, commentary, and correspondence’ (70.6%), ‘expertise in the subject areas in which your journal publishes’ (69.9%), and ‘expertise in dealing with authorship issues’ (60.7%). No items reached consensus for exclusion.

**The degree to which participants perceive they perform particular skills and possess particular experience related to their job as editor.** Of the 20 questions related to participants’ perceptions of how much they thought they performed particular skills and possessed particular experience related to their job as editor, three items reached consensus for inclusion ( Table 2). The highest consensus was for ‘behaving with integrity/professionalism’ (90.2%), followed by ‘using good judgment in decision-making’ (87.5%), and ‘language/writing skills’ (81.5%). No items reached consensus for exclusion.

**Ranked training needs.** Training needs from participants’ ranked top ten lists were categorized into 109 unique items ( Dataset 1). Of the 114 respondents to this question, the top priority listed was training in statistics; mentioned by 36.8% of respondents, with a median ranking of 2 (IQR=2). The second ranked need was for training in research methods; mentioned by 21.9% of respondents, with a median ranking of 2 (IQR=1.5). The third highest training need was in publication ethics; mentioned by 20.2% of respondents, with a median ranking of 3 (IQR=2). The fourth highest need was in recruiting and dealing with peer reviewers; mentioned by 17.5% of respondents, with a median ranking of 3 (IQR=4). The fifth highest training need was in indexing of journals; mentioned by 15.8% of respondents, with a median ranking of 2 (IQR=1).

Ranked list of training needs The dataset lists all of the training needs named by participants (regrouped into categories of similar items) in their respective lists of top 10 training needs from the survey of editors. Click here for additional data file. Copyright: © 2017 Galipeau J et al. 2017 Data associated with the article are available under the terms of the Creative Commons Zero "No rights reserved" data waiver (CC0 1.0 Public domain dedication).

### Modified Delphi

We compiled a list of 202 unique competency-related statements identified in our previous research ^9^ and 12 additional statements identified by participants in the survey of editors in Phase 1 ( Supplementary File S3). These 214 items were categorized into seven areas:

- Journal publishing (29 competency-related statements)
- Publication ethics and research integrity (23 competency-related statements)
- Journal editing (46 competency-related statements)
- Journal promotion (23 competency-related statements)
- Dealing with authors (27 competency-related statements)
- Editor qualities and characteristics (43 competency-related statements)
- Dealing with peer reviewers (23 competency-related statements)

**Round 1.** Eighty-three people participated, all of whom had completed the survey of editors. Of the 214 items listed, 88 items reached consensus for inclusion (≥4 on a 5-point Likert scale). Only one item – ‘act with integrity and accountability’ - was rated as 5 out of 5 by more than 80% of respondents. The items with the highest average score were ‘act with integrity and accountability’ (4.84), ‘identify and address allegations of fraud or plagiarism’ (4.70), ‘act on concerns about plagiarism, data fabrication, or an authorship issue and follow up with authors and then institutions’ (4.65), and ‘request full disclosure of potential conflicts of interest by the authors’ (4.65). No items reached consensus for exclusion (≤2 on a 5-point Likert scale) (see Supplementary File S3 for participants’ comments from all 3 rounds of the Delphi).

**Round 2.** Eighty-three people participated, 80 of whom had completed the survey of editors and 68 of whom participated in Round 1. Of the 214 items listed, 99 items reached consensus for inclusion (≥4 on a 5-point Likert scale). Sixteen items were added for assessment based on the suggestions made by Round 1 participants, of which 4 reached consensus for inclusion, bringing the total of included items to 103. The items with the highest average score in Round 2 were ‘act with integrity and accountability’ (4.82), ‘identify and address allegations of fraud or plagiarism’ (4.68), ‘demonstrate the ability to assess the quality of papers’ (4.64), ‘Demonstrate accountability to authors and ensure they are treated with fairness, courtesy, and objectivity’ (4.63), and ‘request full disclosure of potential conflicts of interest by the authors’ (4.61). Again, no items achieved consensus for exclusion (≤2 on a 5-point Likert scale).

**Round 3.** Round 3 included 73 participants, 72 of whom participated in the survey of editors, 58 of whom participated in both previous rounds, and 10 of whom participated in only one of the two previous rounds. The 103 items that reached consensus in Round 2 were not re-rated in Round 3. Additionally, 5 items with >80% consensus but a rating of between 3.95 and 3.99, as well as one item rated 4.0 but with only 78% consensus were inadvertently left out of the Round 3 Delphi and were therefore added (with a note) to the final list of included competency-related statements. This, therefore, left a total of 121 items to be rated in Round 3. None of these items reached consensus in Round 3, leaving a total of 109 included items at the end of Round 3. Similar to Rounds 1 and 2, no items achieved consensus for exclusion in this round.

Due to the large volume of included items after 3 rounds of the Delphi, the post-hoc decision was made (JG, KDC, LS, DM) to further narrow down the list to a more manageable size. This was done by identifying items that achieved 90% consensus for inclusion in Rounds 2 or 3. This produced 23 “highly rated” items, ( Table 3), leaving 86 “included” items with 80% consensus ( Dataset 2). With no items having reached consensus for exclusion, the Delphi exercise was completed with 121 items that did not reach consensus for inclusion or exclusion.

**Table 3.** Competency Related Statements with 90% consensus (rating of ≥4.5 out of 5).

# *	Competency-Related Statement
1	Demonstrate accountability to authors and ensure they are treated with fairness, courtesy, and objectivity
2	Provide constructive criticism to authors
3	Act on concerns about plagiarism, data fabrication, or an authorship issue and follow up with authors and then institutions
4	Request full disclosure of potential conflicts of interest by the authors
5	Develop, facilitate, and monitor the peer review process
6	Ensure that peer review panels for individual papers are not biased
7	Synthesize reviews and make ultimate editorial decisions in light of peer reviewers' comments
8	Evaluate manuscripts in light of reviewers' critiques and various selection criteria
9	Demonstrate knowledge of the goals of the journal
10	Ensure decisions are based on the validity of the work and its importance to the journal's readers
11	Demonstrate the ability to assess the quality of papers
12	Ensure papers selected are suitable to the journal
13	Demonstrate familiarity with the principles of scientific investigation
14	Demonstrate knowledge of and adherence to the principles of editorial independence
15	Demonstrate expertise in ensuring the ethical integrity of publications
16	Identify and address allegations of fraud or plagiarism
17	Demonstrate understanding of privacy, confidentiality, and anonymity issues
18	Identify and address issues related to conflicts of interest
19	Separate decision-making from commercial considerations
20	Ensure the respect and privacy of patients described in clinical studies
21	Communicate clearly with others
22	Demonstrate effective critical appraisal skills
23	Act with integrity and accountability

*The competencies are presented in the order in which they appeared in the Delphi.

All data for Delphi The dataset is a summary of the data collected over the three rounds of the Delphi process. We considered items with 80% consensus of 4 or higher (out of 5) as "Included". and items with 90% consensus of 4.5 or higher as "Highly Ranked". Click here for additional data file. Copyright: © 2017 Galipeau J et al. 2017 Data associated with the article are available under the terms of the Creative Commons Zero "No rights reserved" data waiver (CC0 1.0 Public domain dedication).

## Discussion

The results from the survey of editors revealed some patterns. First, every item on the list of participant perceptions ( Table 2) occupied the same positional ranking for both the degree to which participants perceived an item as important and the degree to which they perceived that they possessed the expertise or skill. Also, consensus was higher on every item for the degree to which participants perceived particular skills or expertise as important compared to the degree to which they possessed each of these skills. This would seem to indicate that the expertise and skills that editors thought were most important were also those for which they believed themselves to be most competent. However, in examining the data more closely, we can see that the largest gaps between the perceived importance and the perceived possession of particular expertise and skills occurred among items where consensus for inclusion was achieved. In particular, ‘expertise in research methods’, ‘expertise in dealing with publication ethics…’, ‘language/writing skills’, and ‘author and peer reviewer correspondence…’ all had near or above double –digit differences between perceptions of the degree of importance vs. the degree to which participants believed they possessed this expertise or skill. This finding could point to the possibility that despite having more training in the areas they deem most important, editors still may not feel adequately trained in some of these areas.

When comparing the top five items on participants’ ranked list of training needs with editors’ perceptions, their perceptions of competency in these areas (based on similarly-themed items in the respondent’s perceptions of their own knowledge, skills, and abilities) varied from moderate competency (for research methods, publication ethics, and recruiting and dealing with peer reviewers) to low competency (for statistics and indexing).

In the Delphi, participants were quite consistent in their ratings, reflected by the fact that no new items were scored as a 4 or above by 80% of participants after the first round of ratings. Also, although it appears from our sample that editors believed that nearly all of the knowledge, skills, and abilities identified in the scoping review and environmental scan were at least somewhat important to the role of scientific editor, none of these items were rated 2 or lower by 80% of respondents in any round of the Delphi. This could indicate that editors see their role as encompassing a very large number of important interrelated skills, abilities, and knowledge.

Generally, there appears to be some agreement between the survey of editors’ perceptions and the Delphi process across many items. When provided with an expanded list of potential competencies in the Delphi (from 38 items in the survey of editors to 230 items in the Delphi), many of the highest rated items from the survey of editors’ perceptions remained among the highest rated in the Delphi. For example, of the six items that achieved consensus in the survey of editors’ perceptions, four were similar to items on the ‘highly rated’ list from the Delphi, while the remaining two (‘writing/language skills’ and ‘using good judgment in decision-making’) were similar to items in the ‘included’ list from the Delphi. Additionally, the top 5 items listed in participants’ top 10 training needs were all included in the final list of competency-related statements arising from the Delphi, with the 2 ^nd^, 3 ^rd^, and 4 ^th^ most cited training needs similar to items in the ‘highly rated’ list from the Delphi. This finding may suggest that some of the most critical skills related to the position of scientific editor may also be some of the ones for which editors feel the least trained. However, it’s unclear whether this is due to the central importance of these elements (and the need for thorough, ongoing training), a true lack of training (whether in terms of availability or quality), both of these factors, or some other reason(s).

### Limitations

There were a number of limitations in this research. The limited number of respondents for both the survey of editors’ perceptions and the modified Delphi, the fact that the study was conducted in English, and the fact that the survey was completed primarily by medical journal editors may limit the generalizability of the findings to the wider pool of scientific editors around the world. Additionally, for the modified Delphi, we chose to only invite respondents from the survey of editors (however we did not restrict participation to only this group). This decision was made since we believed we had used all of the most pertinent communication channels to recruit participants for the survey of editors, therefore (given our biomedical focus) we were unlikely to gain many more respondents by putting out a further call for participation in the modified Delphi. Moreover, although efforts were made to regroup similar items from participants’ top 10 lists of training needs, this subjective process may have failed to regroup some items that could be seen as similar while combining other items that could be judged to be different from one another. A further limitation is in regards to the interpretation of competency-related statements to be rated by participants in the Delphi. While efforts were made to preserve the original wording of competency-related statements in the scoping review, some of the participants’ comments relayed difficulties in understanding some items and misinterpreting others. This may have led to the mis-rating of a few items by some participants. This limitation should be offset at least to some extent by our use of median scores rather than means.

## Conclusion

This research provides an insight into the perceptions of scientific editors of biomedical journals from around the world regarding the importance of particular expertise and skills in their role as scientific editors and the degree to which they believe they possess these competencies. This information complements the competency-related statements identified in the scoping review, as it enabled those who might benefit from educational efforts to identify the most important competency-related statements from their standpoint and to self-identify their greatest needs. Together with a previous scoping review and environmental scan of the literature, these findings were used to inform a consensus-building process in which a minimum set of core competencies for scientific editors of biomedical journals was determined at a consensus meeting by a wide-ranging group of stakeholders (to be reported in a future paper). The findings from both the survey and the modified Delphi process reported on here are critical for understanding knowledge and training gaps among scientific editors when designing curriculum around these core competencies in the future.

## Data Availability

The data referenced by this article are under copyright with the following copyright statement: Copyright: © 2017 Galipeau J et al.

Data associated with the article are available under the terms of the Creative Commons Zero "No rights reserved" data waiver (CC0 1.0 Public domain dedication). 

Dataset 1. Ranked list of training needs

The dataset lists all of the training needs named by participants (regrouped into categories of similar items) in their respective lists of top 10 training needs from the survey of editors.

10.5256/f1000research.12400.d175998 ^16^

Dataset 2. All data for Delphi

The dataset is a summary of the data collected over the three rounds of the Delphi process. We considered items with 80% consensus of 4 or higher (out of 5) as "Included", and items with 90% consensus of 4.5 or higher as "Highly Ranked".

10.5256/f1000research.12400.d175999 ^17^

## Supplementary material

Supplementary File S1 – Questions for survey of editors’ perceptions.

Supplementary File S2 – Text answers for survey of editors’ perceptions.

Supplementary File S3 – Delphi text responses for all 3 rounds.
