# Supplementary material for: An international survey and modified Delphi process revealed editors’ perceptions, training needs, and ratings of competency-related statements for the development of core competencies for scientific editors of biomedical journals
Source: F1000Res. 2017 Sep 4;6:1634. [Version 1] doi: 10.12688/f1000research.12400.1 (PMC5605946; doi:10.12688/f1000research.12400.1)
Supplement: Ranked list of training needs — The dataset lists all of the training needs named by participants (regrouped into categories of similar items) in their respective lists of top 10 training needs from the survey of editors. [file f1000research-6-13429-s0000.tgz › 3943cff6-e206-42c9-9832-db0e13500cda_Dataset_1.docx]

**Dataset 1 – Ranked List of Training Needs**

The following is a table listing all of the training needs named by participants (regrouped into categories of similar items) in their respective lists of top 10 training needs from the survey of editors.

| **# Mentions^1^** | **Median Rank^2^** | **Description^3^** | **Category From Delphi Process^4^** | **Related Item #^5^** |
| --- | --- | --- | --- | --- |
| 42 | 2 | Statistics | Editing | 136 |
| 25 | 2 | Research Methods | Editing | 138 |
| 23 | 3 | Publication Ethics | Ethics and integrity | 151 |
| 20 | 3 | Recruiting and dealing with peer reviewers | Dealing with Peer Reviewers | 28 |
| 18 | 2 | Indexing | Journal promotion | 53 |
| 17 | 3 | Social Media | Journal promotion | 101 |
| 15 | 2 | Misconduct | Ethics and integrity | 20 |
| 12 | 2.5 | Managerial Skills | Qualities and characteristics of editors | 213 |
| 11 | 3 | Peer Review models and process | Dealing with peer reviewers | 38 |
| 11 | 4 | Metrics | Journal promotion | 67 |
| 11 | 4 | Business Skills | Qualities and characteristics of editors | 70 |
| 10 | 2 | Increasing manuscript submissions | Authors | 89 |
| 10 | 3 | Finding, Training and supervising editors | Journal publishing | 127 |
| 9 | 3 | Journal Promotion | Journal promotion | 92 |
| 9 | 3 | Working with authors | Dealing with authors | 8 |
| 8 | 3 | Publication Process | Journal publishing | 104 |
| 8 | 3 | Conflict of Interest | Ethics and integrity | 154 |
| 8 | 3.5 | Public Relations | Journal promotion | 192 |
| 7 | 2 | Editorial Board Selection and Organization | Journal publishing | EIC |
| 7 | 3 | Online Publication | Journal publishing | 68 |
| 6 | 2.5 | Copyright/Creative Commons | Journal publishing | 167; 79 |
| 5 | 2 | Technology | Journal publishing | 126 |
| 5 | 2 | Improving the journal impact | Journal promotion | 86 |
| 5 | 3 | Open Access | Journal publishing | 77 |
| 5 | 5 | Journal submission management systems | Journal publishing | 125 |
| 4 | 3 | Plagiarism detection software | Journal publishing | 126 |
| 4 | 1.5 | Succession planning | Journal publishing | 229 |
| 4 | 2.5 | Dealing with publisher | Journal publishing | EIC |
| 4 | 1.5 | Working with other editors | Journal publishing | 145 |
| 3 | 1 | Public communication/outreach | Journal promotion | 99 |
| 3 | 1 | Clinical relevance | Editing | 111 |
| 3 | 2 | Editorial Process | Editing | 60 |
| 3 | 2 | The new GRADE program | Journal publishing | 85 |
| 3 | 2 | Legal (Liabilities) | Journal publishing | 62 |
| 3 | 3 | Authorship | Ethics and integrity | 22 |
| 3 | 3 | Time management | Qualities and characteristics of editors | 180 |
| 3 | 3 | Role of editorial team | Journal publishing | 65 |
| 3 | 3 | Publication Models | Journal publishing | 77 |
| 3 | 3 | Improving efficiency and turnaround times | Journal publishing | 180 |
| 3 | 4 | Training Peer Reviewers | Dealing with peer reviewers | 39 |
| 3 | 5 | Predatory publishing | Journal publishing | 78 |
| 3 | 5 | Mass Media | Journal promotion | 122 |
| 2 | 1 | Summary of findings tables- editing | Editing | 133 |
| 2 | 1 | Critical appraisal of Research papers | Editing | 189 |
| 2 | 1.5 | Expertise with supplemental material | Editing | 144 |
| 2 | 1.5 | Open journal system | Journal publishing | 125 |
| 2 | 1.5 | Qualitative Research | Editing | 139 |
| 2 | 2 | New technologies affecting publishing | Journal publishing | 72 |
| 2 | 3 | Journal Production | Journal publishing | 71 |
| 2 | 3 | Writing Skills | Editing | 132 |
| 2 | 3.5 | Tables, figures, and other graphics | Journal publishing | 132 |
| 2 | 3.5 | Working with an editorial board | Journal publishing | 210 |
| 2 | 4 | Language editing | Editing | 230 |
| 2 | 4 | Feedback to authors | Dealing with authors | 7 |
| 2 | 4 | Increasing quality of submitted manuscripts | Dealing with authors | 8 |
| 2 | 5 | Dealing with Owner | Journal publishing | EIC |
| 2 | 5.5 | writing news releases | Journal promotion | 102 |
| 2 | 6 | Assessing how authors have made inclusion/exclusion decisions | Dealing with authors | 141 |
| 2 | 8 | Support for managers when training new editorial staff | Journal publishing | 173 |
| 1 | 1 | The correct procedures of clinical trials, and the legal framework in which clinical trials are undertaken. | Journal Publishing | 62 |
| 1 | 1 | Managing Peer Review | Dealing with peer reviewers | 28 |
| 1 | 1 | Journal Publishing Style | Journal publishing | 57 |
| 1 | 1 | Role of co-ordinating editor | Journal publishing | 65 |
| 1 | 1 | Article Management | Editing | 104 |
| 1 | 5 | Journal website development | Journal publishing | 68 |
| 1 | 1 | Continuous Professional Development of Cochrane changes to guidance and methods | Qualities and characteristics of editors | 80 |
| 1 | 1 | Assessing Readers' Views, Interests, and Needs | Journal promotion | 94 |
| 1 | 1 | Data access and publishing requirements | Journal publishing | 167 |
| 1 | 1 | Medical sciences | N/A | 172 |
| 1 | 1 | Public health | N/A | 172 |
| 1 | 1 | Changing publishers' money making schemes | Journal publishing | EIC |
| 1 | 1 | Handling conflict | Qualities and characteristics of editors | 179 |
| 1 | 1 | How to help journal ownership understand the importance of quality of reporting | Journal publishing | EIC |
| 1 | 2 | Decision making in the face of conflicting peer review | Editing | 41 |
| 1 | 2 | Marketing | Journal promotion | 51 |
| 1 | 2 | Editorial standards | Editing | 60 |
| 1 | 2 | Enhancing readability of scientific material | Journal publishing | 63 |
| 1 | 2 | Understanding relationships between academic editors and publishers | Journal publishing | 65 |
| 1 | 2 | Quality of Reporting | Editing | 75 |
| 1 | 2 | Understanding journal decision-making context | Journal publishing | 80 |
| 1 | 2 | Advances in Biomedical sciences |  | 85 |
| 1 | 2 | Communication of evidence | Journal publishing | 90 |
| 1 | 2 | Application of editorial policies to submitted articles - worked examples | Editing | 103 |
| 1 | 2 | Laboratory techniques, esp. related to genetics research | Editing | 138 |
| 1 | 2 | Meta-Analysis | Editing | 136 |
| 1 | 2 | Womens' Health | Qualities and Characteristics of Editors | 172 |
| 1 | 2 | How to improve international outreach | Journal promotion | EIC |
| 1 | 2 | Influencing public policy | Journal promotion | EIC |
| 1 | 3 | How to encourage reviewers to generate a review without determining acceptance or rejection | Dealing with peer reviewers | 32 |
| 1 | 3 | Reproduction sciences | N/A | 172 |
| 1 | 3 | linking content to current affairs (journalistic skills) | Journal promotion | 214 |
| 1 | 3 | Data analysis | Journal publishing | 216 |
| 1 | 3 | Licenses | Journal publishing | EIC |
| 1 | 4 | Innovating to maintain usefulness for changing demographics of readers | Journal promotion | 98 |
| 1 | 4 | How to attract advertisements | Journal promotion | EIC |
| 1 | 5 | Publishing beyond journals | Journal publishing | 74 |
| 1 | 5 | Scientific journalism |  | 122 |
| 1 | 5 | Data sharing | Journal publishing | 146 |
| 1 | 5 | Referencing software | Editing | 147 |
| 1 | 5 | Funding | Journal publishing | EIC |
| 1 | 5 | Negotiation - with publishers and journal owners | Journal publishing | EIC |
| 1 | 5 | Which language to choose to publish our articles- French or English | Journal publishing | EIC |
| 1 | 6 | Decision making | Qualities and characteristics of editors | 119 |
| 1 | 6 | DOI Assignment | Journal publishing | 148 |
| 1 | 7 | Consolidate major journal organizations & meetings | Journal publishing | EIC |
| 1 | 8 | The role of chief editors at the current stage | Journal publishing | 65 |
| 1 | 8 | More information on EQUATOR, COMET, etc. | Editing | 84 |
| 1 | 9 | Letters to the editor | Journal publishing | 142 |
| 1 | 10 | Reporting Guidelines | Editing | 161 |

**^1^**Total number of times the item was named by all participants in their top 10 list of training needs.

^2^Median score of all mentions for the item. If only 1 mention for an item, then this score was entered.

**^3^**Term used by participants. In order to categorize and rank items, we attempted to regroup similar terms under a single description. Thus not all items were identical to the term used in this list.

**^4^**Links the term back to the 7 categories used to classify the competency-related statements

**^5^**Links the term back to a particular item(s) in the list of 230 competency-related statements. EIC indicates that the item was unique to Editors-in Chief (not classified in the competency-related statements).

Item is listed as “highly ranked” in the final results of the Delphi process

Item is listed as “included” in the final results of the Delphi process
