# Supplementary material for: An international survey and modified Delphi process revealed editors’ perceptions, training needs, and ratings of competency-related statements for the development of core competencies for scientific editors of biomedical journals
Source: F1000Res. 2017 Sep 4;6:1634. [Version 1] doi: 10.12688/f1000research.12400.1 (PMC5605946; doi:10.12688/f1000research.12400.1)
Supplement: All data for Delphi — The dataset is a summary of the data collected over the three rounds of the Delphi process. We considered items with 80% consensus of 4 or higher (out of 5) as "Included". and items with 90% consensus of 4.5 or higher as "Highly Ranked". [file f1000research-6-13429-s0001.tgz › 0948e702-d17b-4a2e-8f4f-7150672a3de7_Dataset_2.pdf]

APPENDIX D - All data for Delphi

Below is a summary of the data collected over the three rounds of the Delphi process. The first round consisted of having participants rate all of the 202 items from the scoping review, as well as the 12 new items that emerged from the needs assessment, based on a 5-point rating scale. Participants were also invited to suggest any additional items that they felt should be included to the list. The second round allowed participants to see the average (group) rating from Round 1 of each item along with their own rating and all of the comments made by participants reagrding the items. They were then asked to re-rate only those items for which they disagreed with the average score (and to provide a justification for their new rating). In addition, participants were asked to rate 14 new items that were suggested in Round 1. In the thrid round, only the items for which consesnsus (for inclusion or exclusion) was not reached were included. Participants were provided with average scores and comments and then asked to rate these items a final time, based on a 3-point rating scale. More details regarding each column of data are available below the table.

INTERPRETATION: We considered items with 80% consensus of 4 or higher (out of 5) as "Included". and items with 90% consensus of 4.5 or higher as "Highly Ranked".

		ROUND 1[1]				ROUND 2[6]					ROUND 3[13]		FINAL
Item #	Competency-Related Statements	# Participants for Round 1[2]	Round 1 Average Score[3]	% Agreement for ≥4[4]	% Agreement for ≤2[5]	# Participants That Made Changes in Round 2[7]	Round 2 Average Score[8]	% Agreement for ≥4[9]	% Agreement for ≥4.5[10]	% Agreement for ≤2.5[11]	# Participants for Round 3[14]	% Choosing "Essential" Option[15]	Final Status[16]
**Dealing With Authors**													
1	Review study protocols and methods and encourage authors to make them publicly available	81	3.81	69%	12%	21	3.80	20%	7%	3%	72	46%	Not Included
2	Ensure authors are aware of ethical authorship practices	81	**4.14**	81%	6%	14	**4.06**	95%	5%	3%	N/A	N/A	Included
3	Seek to help authors understand magnitude of effect	79	3.27	43%	27%	16	3.22	11%	4%	11%	72	18%	Not Included
4	Assist potential authors in developing a spirit of inquiry	81	3.05	36%	32%	15	2.99	7%	3%	10%	73	14%	Not Included
5	Develop wide acquaintance with potential authors	81	2.94	35%	38%	17	3.01	13%	4%	8%	73	14%	Not Included
6	Demonstrate accountability to authors and ensure they are treated with fairness, courtesy, and objectivity	80	**4.63**	99%	0%	9	**4.63**	99%	94%	0%	N/A	N/A	Highly Ranked
7	Provide constructive criticism to authors	81	**4.57**	94%	0%	10	**4.60**	98%	96%	0%	N/A	N/A	Highly Ranked
8	Engage in mentorship and education of authors to help them produce work to best effect	81	3.28	52%	22%	21	3.25	16%	1%	7%	72	35%	Not Included
9	Mediate sound communication between the comments of reviewers and responses of authors	80	**4.34**	91%	4%	10	**4.30**	95%	12%	3%	N/A	N/A	Included
10	Ensure publication decisions are clearly communicated to all authors	81	**4.51**	95%	0%	8	**4.48**	99%	89%	0%	N/A	N/A	Included
11	Interact with authors to confirm undisputed changes in authorship and act on any institutional findings concerning authorship disputes	78	3.95	67%	6%	11	3.89	13%	7%	4%	73	70%	Not Included
12	Clarify the peer-review processes to authors	81	3.99	73%	4%	13	3.97	19%	7%	2%	73	74%	Not Included
13	Negotiate manuscript publication delays with authors	81	3.46	49%	14%	11	3.36	7%	0%	6%	73	25%	Not Included
14	Deal with authors who appeal against rejection	80	**4.14**	81%	4%	8	**4.19**	98%	8%	0%	N/A	N/A	Included
15	Ensure authors are informed about journal and article information and/or funding	80	3.64	63%	14%	12	3.56	11%	4%	6%	73	36%	Not Included
16	Ensure that requests from authors that an individual not review their submission are respected, if these are well-reasoned	80	4.00	79%	5%	9	**3.95***	94%	2%	1%	N/A	N/A	Included
17	Engage in critical evaluation of authors' manuscripts and the peer-review process itself	81	**4.20**	81%	5%	8	**4.15**	95%	7%	1%	N/A	N/A	Included
18	Provide active encouragement for revisions of manuscripts	81	3.73	62%	10%	15	3.67	15%	1%	4%	72	56%	Not Included
19	Demonstrate experience as a competent author, academic, researcher or reviewer	81	**4.19**	83%	9%	9	**4.12**	95%	10%	4%	N/A	N/A	Included
20	Demonstrate proficiency in dealing with author misconduct and other issues related to publication ethics	81	**4.44**	91%	2%	6	**4.40**	97%	9%	1%	N/A	N/A	Included
21	Work with publishers to defend author rights and pursue offenders	80	3.50	59%	20%	8	3.41	7%	3%	6%	72	35%	Not Included
22	Act on concerns about plagiarism, data fabrication, or an authorship issue and follow up with authors and then institutions	80	**4.65**	96%	4%	6	**4.56**	97%	92%	3%	N/A	N/A	Highly Ranked
23	Request full disclosure of potential conflicts of interest by the authors	80	**4.65**	96%	1%	6	**4.61**	99%	93%	1%	N/A	N/A	Highly Ranked
24	Support authors in dealing with breaches of copyright and plagiarism issues	81	3.77	63%	11%	10	3.72	13%	4%	4%	73	53%	Not Included
25	Request appropriate documentation from authors when they submit manuscripts	81	**4.07**	77%	9%	4	**3.97***	92%	4%	3%	N/A	N/A	Included
26	Demonstrate the ability to work with authors from developing countries	79	3.78	62%	6%	18	3.78	18%	7%	3%	72	49%	Not Included
27	Assist non-native speakers in dealing with language issues	81	3.04	35%	35%	20	2.98	7%	3%	11%	72	31%	Not Included
**Dealing With Peer Reviewers**													
28	Develop, facilitate, and monitor the peer review process	79	**4.56**	94%	0%	7	**4.57**	99%	94%	0%	N/A	N/A	Highly Ranked
29	Knowledge of different types of peer review	79	3.99	71%	4%	11	3.95	16%	5%	2%	71	66%	Not Included
30	Encourage and demonstrate awareness of new findings on peer review and publishing and how these influence their journal's processes	78	3.90	72%	6%	8	3.86	14%	2%	2%	71	51%	Not Included
31	Review revised manuscripts	79	**4.27**	82%	4%	8	**4.26**	98%	9%	2%	N/A	N/A	Included
32	Provide guidance to peer reviewers	79	**4.23**	85%	5%	10	**4.25**	98%	10%	1%	N/A	N/A	Included
33	Ensure thorough statistical review	78	**4.32**	82%	0%	9	**4.30**	95%	9%	0%	N/A	N/A	Included
34	Ensure that peer review panels for individual papers are not biased	79	**4.57**	95%	0%	7	**4.55**	99%	91%	0%	N/A	N/A	Highly Ranked
35	Evaluate and provide feedback to the reviewers on review quality	79	3.82	71%	8%	15	3.76	14%	5%	3%	72	58%	Not Included
36	Ensure manuscripts' content is matched with the expertise of particular reviewers	79	**4.35**	87%	1%	7	**4.34**	96%	9%	0%	N/A	N/A	Included
37	Monitor and ensure the fairness, timeliness, thoroughness and civility in the processing of manuscripts and in responding to queries from authors and reviewers	79	**4.43**	90%	0%	4	**4.39**	97%	6%	0%	N/A	N/A	Included
38	Demonstrate knowledge of the workings of the peer review process	79	**4.46**	92%	1%	5	**4.45**	99%	9%	0%	N/A	N/A	Included
39	Train peer reviewers	79	3.14	38%	27%	19	3.07	7%	2%	8%	72	31%	Not Included
40	Ensure reviewer comments are shared with all peer reviewers	77	3.25	45%	23%	15	3.21	8%	4%	6%	72	44%	Not Included
41	Synthesize reviews and make ultimate editorial decisions in light of peer reviewers' comments	79	**4.52**	90%	1%	6	**4.53**	98%	94%	0%	N/A	N/A	Highly Ranked
42	Evaluate manuscripts in light of reviewers' critiques and various selection criteria	77	**4.58**	95%	0%	5	**4.57**	99%	91%	0%	N/A	N/A	Highly Ranked
43	Demonstrate the ability to distinguish between objective peer reviewed research and reviews from opinion and the journal content from advertising and other promotional content	78	**4.37**	90%	4%	7	**4.30**	96%	7%	3%	N/A	N/A	Included
44	Ensure reviewers who consistently produce discourteous, poor quality or late reviews are removed from the journal's pool of peer reviewers	79	**4.41**	89%	3%	6	**4.34**	96%	6%	1%	N/A	N/A	Included
45	Ensure a decision is made on a manuscript when reviewers fail to submit a timely review	79	**4.23**	87%	1%	6	**4.21**	97%	5%	0%	N/A	N/A	Included
46	Ensure a very high standard of the referees don't accept sloppy reports from anyone	79	3.82	67%	9%	16	3.78	17%	5%	3%	71	77%	Not Included
47	Demonstrate publication and reviewing skills and experience	79	**4.24**	85%	5%	6	**4.17**	96%	7%	3%	N/A	N/A	Included
48	Ensure that reviewers keep manuscripts, associated material, and the information they contain strictly confidential.	79	**4.19**	82%	8%	9	**4.08**	94%	6%	5%	N/A	N/A	Included
49	Demonstrate sound judgment in the acceptance of research articles, editorials, and reviews that touch on current issues	79	**4.42**	91%	3%	4	**4.40**	98%	8%	1%	N/A	N/A	Included
50	Demonstrate the ability to successfully recruit peer reviewers	78	3.97	74%	5%	11	3.98	16%	7%	0%	71	75%	Not Included
**Journal Publishing**													
51	Demonstrate knowledge of marketing and advertising policies, including ethical issues	81	3.57	58%	15%	11	3.59	15%	7%	5%	69	35%	Not Included
52	Demonstrate knowledge of the article embargo process	80	3.69	60%	9%	11	3.64	13%	3%	3%	70	47%	Not Included
53	Demonstrate knowledge of indexing services	81	3.26	46%	28%	12	3.25	13%	6%	8%	71	25%	Not Included
54	Demonstrate knowledge of reprint processes	80	2.55	21%	48%	13	2.55	7%	3%	14%	71	7%	Not Included
55	Demonstrate knowledge of the specifications of the journal	81	3.88	73%	14%	12	3.88	19%	7%	4%	69	65%	Not Included
56	Demonstrate knowledge of the goals of the journal	81	**4.54**	93%	1%	7	**4.54**	100%	91%	0%	N/A	N/A	Highly Ranked
57	Demonstrate knowledge of formatting of layout for journal issues	81	3.05	37%	37%	17	3.09	11%	4%	7%	70	23%	Not Included
58	Ensure the content of manuscripts submitted for publication is checked for accuracy	81	**4.28**	88%	2%	10	**4.22**	95%	9%	2%	N/A	N/A	Included
59	Demonstrate knowledge of the different parts, purposes, and characteristics of different types of journals	81	3.48	52%	15%	9	3.50	13%	4%	3%	71	31%	Not Included
60	Demonstrate understanding of the editorial office and operations	80	3.85	70%	9%	8	3.85	15%	7%	2%	71	56%	Not Included
61	Ensure that selected/published research is correct	79	**4.41**	89%	4%	7	**4.37**	95%	12%	2%	N/A	N/A	Included
62	Demonstrate knowledge about legal issues relating to the position of scientific editor	81	**4.01**	72%	5%	8	**4.02**	94%	7%	0%	N/A	N/A	Included
63	Be aware of how design can be used to improve the readability of a document	80	3.25	51%	29%	13	3.22	12%	3%	8%	71	24%	Not Included
64	Demonstrate understanding of one's responsibilities and rights as a journal editor	81	**4.47**	89%	2%	4	**4.49**	99%	12%	0%	N/A	N/A	Included
65	Demonstrate knowledge of the roles and responsibilities of the editorial staff	81	**4.23**	80%	1%	6	**4.20**	94%	8%	0%	N/A	N/A	Included
66	Identify and address issues related to data protection and confidentiality	81	**4.22**	79%	2%	4	**4.17**	93%	9%	1%	N/A	N/A	Included
67	Demonstrate knowledge of journal metrics and research impact	81	3.74	68%	11%	8	3.75	15%	7%	4%	71	54%	Not Included
68	Demonstrate knowledge of online publishing and products	80	3.39	49%	19%	6	3.40	11%	5%	5%	70	37%	Not Included
69	Demonstrate knowledge of the parts, purposes, and characteristics of audio and video clips	81	2.62	23%	47%	9	2.60	5%	1%	12%	71	7%	Not Included
70	Demonstrate awareness of intellectual property issues and work with publisher to handle potential breaches	80	3.75	68%	14%	5	3.70	11%	5%	3%	71	56%	Not Included
71	Demonstrate knowledge of technical-economical aspects of medical journal production	81	2.77	25%	40%	7	2.73	5%	2%	10%	70	13%	Not Included
72	Explore and embrace innovative technologies	81	3.31	51%	22%	12	3.25	11%	2%	8%	71	21%	Not Included
73	Maintain close contact with the latest trends in electronic media (e.g., tablets)	81	3.09	42%	35%	15	3.07	12%	5%	11%	70	20%	Not Included
74	Engage in multimedia publishing practices	81	2.85	33%	43%	9	2.87	8%	1%	7%	71	13%	Not Included
75	Act as a gatekeeper and guarantor of publications, checking both the quality and scope of research published in the journal	81	**4.27**	85%	5%	4	**4.19**	95%	7%	3%	N/A	N/A	Included
76	Demonstrate knowledge of the parts, purposes, and characteristics of manuscripts	81	**4.25**	84%	2%	4	**4.20**	96%	7%	2%	N/A	N/A	Included
77	Demonstrate knowledge of open access models	81	3.68	63%	14%	9	3.61	13%	3%	6%	71	55%	Not Included
78	Demonstrate knowledge of issues related to predatory publishing	79	3.81	71%	14%	11	3.75	15%	3%	4%	71	55%	Not Included
79	Demonstrate understanding of Creative Commons licensing	79	3.49	56%	25%	10	3.47	11%	8%	7%	71	41%	Not Included
**Journal Promotion**													
80	Maintain knowledge of important developments and trends in one's own field	79	**4.24**	89%	1%	4	**4.21**	98%	4%	0%	N/A	N/A	Included
81	Demonstrate knowledge of history of journals and scientific publications	79	2.86	24%	34%	7	2.85	6%	2%	8%	70	11%	Not Included
82	Demonstrate knowledge of national and regional variations between journals	79	3.10	37%	29%	11	3.08	9%	2%	8%	71	17%	Not Included
83	Demonstrate knowledge of political and geopolitical issues	79	2.94	30%	33%	11	2.89	6%	1%	11%	70	10%	Not Included
84	Demonstrate familiarity with associations and their educational resources	78	3.13	38%	29%	6	3.10	7%	1%	7%	71	15%	Not Included
85	Stay on top of updates in one's field	79	**4.19**	86%	1%	4	**4.13**	97%	2%	1%	N/A	N/A	Included
86	Demonstrate knowledge of, and work to maintain and improve the journal's policies, vision, scope, content, processes, and goals	77	**4.40**	86%	4%	4	**4.31**	96%	5%	2%	N/A	N/A	Included
87	Ensure decisions are based on the validity of the work and its importance to the journal's readers	79	**4.61**	95%	0%	4	**4.59**	99%	91%	0%	N/A	N/A	Highly Ranked
88	Ensure controversial topics (political, ethical) are dealt with	79	3.94	75%	9%	8	3.93	16%	7%	2%	70	46%	Not Included
89	Stimulate others to write articles and editorials	79	3.86	72%	9%	9	3.83	15%	4%	3%	71	51%	Not Included
90	Engage in the promotion of scholarly research and best practices in conducting and reporting it	79	**4.24**	84%	6%	8	**4.22**	96%	8%	1%	N/A	N/A	Included
91	Entice leading researchers to submit to the journal	78	3.83	72%	12%	7	3.82	14%	4%	2%	71	52%	Not Included
92	Serve as ambassador for the journal in establishing its visibility and image	79	**4.33**	87%	5%	7	**4.31**	98%	8%	1%	N/A	N/A	Included
93	Motivate physicians to read, ponder, and implement the information provided	78	3.62	62%	18%	10	3.55	11%	3%	5%	69	38%	Not Included
94	Seek feedback/opinions on the journal	79	3.91	71%	11%	7	3.93	15%	10%	3%	71	49%	Not Included
95	Enhance public understanding of science	79	3.46	56%	20%	13	3.41	11%	4%	6%	71	18%	Not Included
96	Demonstrate understanding of who one's constituency is	77	3.65	58%	14%	6	3.55	6%	3%	4%	71	38%	Not Included
97	Demonstrate a responsibility to the scientific community	79	**4.14**	85%	5%	6	**4.07**	94%	6%	2%	N/A	N/A	Included
98	Hold paramount the interests of the particular journal's readers	78	3.79	65%	9%	7	3.77	11%	6%	1%	71	51%	Not Included
99	Engage in communication with the public	79	3.11	38%	27%	11	3.12	9%	3%	6%	71	10%	Not Included
100	Engage with existing and new scientific communities	78	3.54	54%	13%	7	3.55	10%	4%	1%	71	25%	Not Included
101	Engage with social media to reach out beyond the usual specialist audiences	79	3.18	42%	27%	9	3.21	9%	6%	6%	71	17%	Not Included
102	Demonstrate proficiency with writing news releases	79	2.95	35%	38%	15	2.97	11%	2%	9%	71	8%	Not Included
**Editing**													
103	Demonstrate knowledge of policies for submission of manuscripts	80	**4.25**	83%	3%	3	**4.26**	100%	5%	0%	N/A	N/A	Included
104	Demonstrate broad and detailed knowledge of the skills needed to refine a piece of scientific work and shepherd it through to publication	81	**4.32**	85%	1%	4	**4.24**	95%	6%	1%	N/A	N/A	Included
105	Demonstrate knowledge of typography	82	2.51	21%	54%	13	2.46	4%	1%	16%	70	9%	Not Included
106	Demonstrate knowledge of and experience with online editing	82	3.12	43%	33%	6	3.15	9%	4%	7%	70	24%	Not Included
107	Demonstrate knowledge of the fundamentals of editing various types of manuscripts	81	3.57	53%	19%	10	3.56	13%	7%	6%	69	39%	Not Included
108	Enforce ICMJE authorship guidelines	81	**4.10**	81%	7%	5	**4.04**	95%	5%	4%	N/A	N/A	Included
109	Ensure logic and consistency of manuscripts	82	**4.32**	89%	2%	5	**4.27**	96%	6%	1%	N/A	N/A	Included
110	Demonstrate the ability to assess the quality of papers	81	**4.64**	100%	0%	5	**4.64**	100%	92%	0%	N/A	N/A	Highly Ranked
111	Ensure papers selected are clinically relevant	82	3.85	63%	10%	10	3.80	15%	7%	5%	69	64%	Not Included

112	Ensure papers selected have a clear story-line	81	3.73	63%	11%	8	3.68	16%	3%	4%	70	60%	Not Included
113	Demonstrate the ability to select material for its merit, interest to readers, and originality alone	82	**4.06**	76%	9%	6	4.00	92%	8%	3%	N/A	N/A	Included
114	Ensure papers selected are suitable to the journal	82	**4.52**	93%	0%	5	**4.54**	100%	92%	0%	N/A	N/A	Highly Ranked
115	Ensure papers selected for review are meaningful	82	**4.35**	85%	1%	4	**4.34**	98%	8%	0%	N/A	N/A	Included
116	Ensure manuscripts are triaged judiciously (for journals that use such a process)	77	4.00	75%	8%	4	**4.02**	95%	5%	1%	N/A	N/A	Included
117	Demonstrate the ability to form preliminary opinions on a submitted manuscript's relevance	82	**4.02**	77%	4%	4	**4.03**	94%	7%	0%	N/A	N/A	Included
118	Demonstrate the ability to make fast, good decisions about papers	82	**4.16**	83%	5%	1	**4.13**	96%	5%	1%	N/A	N/A	Included
119	Demonstrate the ability to make difficult decisions	82	**4.43**	89%	4%	2	**4.40**	97%	10%	1%	N/A	N/A	Included
120	Demonstrate the ability to exercise excellent judgment	81	**4.43**	91%	2%	3	**4.41**	98%	11%	2%	N/A	N/A	Included
121	Handle manuscripts in the areas of one`s expertise and assist in finding persons qualified to handle papers in those areas outside one`s expertise.	82	**4.44**	90%	0%	2	**4.44**	98%	10%	0%	N/A	N/A	Included
122	Engage in, and maintain interactions and good relations with media	82	3.04	33%	30%	8	3.02	7%	1%	7%	70	13%	Not Included
123	Select, curate, and comment on articles for publication	81	3.81	69%	7%	5	3.74	10%	3%	3%	70	54%	Not Included
124	Ensure alterations recommended based on peer reviewers' comments can be justified	81	**4.25**	89%	1%	3	**4.20**	95%	6%	1%	N/A	N/A	Included
125	Demonstrate experience or familiarity with manuscript tracking software (e.g. ScholarOne, AllenTrack, PeerTrack, BenchPress)	82	3.09	39%	37%	12	3.20	15%	8%	8%	70	30%	Not Included
126	Demonstrate aptitude in using technology (computers, Internet, e-mail, Manuscript Submission Systems) to perform his or her editorial duties	81	3.99	69%	7%	8	3.99	17%	9%	3%	70	77%	Not Included
127	Possess a degree in medical editing or be trained as a journal editor	82	2.60	23%	54%	12	2.65	10%	4%	13%	70	13%	Not Included
128	Demonstrate the ability to write editorials	82	3.21	46%	29%	11	3.23	15%	5%	8%	70	33%	Not Included
129	Demonstrate working knowledge of the language in which the journal is published	81	**4.47**	93%	4%	5	**4.47**	98%	11%	1%	N/A	N/A	Included
130	Demonstrate skills in speed reading, skim reading, and critical reading	82	3.39	44%	21%	10	3.44	14%	6%	4%	69	33%	Not Included
131	Demonstrate an aptitude for reading widely, deeply, and continually	81	3.40	46%	19%	7	3.45	11%	5%	2%	70	33%	Not Included
132	Demonstrate experience and/or training in medical journal writing	82	3.43	55%	24%	7	3.40	14%	4%	6%	69	39%	Not Included
133	Demonstrate understanding of the parts, purposes, and characteristics of tables, charts, graphs, and images	82	**4.04**	76%	5%	7	**4.05**	94%	10%	1%	N/A	N/A	Included
134	Demonstrate familiarity with scientific units, numerals, symbols and nomenclature	82	3.78	60%	6%	5	3.80	13%	8%	2%	70	50%	Not Included
135	Demonstrate familiarity with the presentation of data and data presentation problems	82	**4.10**	80%	2%	3	**4.05**	92%	8%	2%	N/A	N/A	Included
136	Demonstrate familiarity with the basic concepts of statistics	82	**4.16**	76%	2%	5	**4.18**	94%	11%	0%	N/A	N/A	Included
137	Demonstrate knowledge of literature reviews	82	3.85	73%	12%	8	3.85	15%	8%	2%	69	62%	Not Included
138	Demonstrate familiarity with the principles of scientific investigation	82	**4.50**	94%	0%	5	**4.50**	99%	91%	0%	N/A	N/A	Highly Ranked
139	Demonstrate familiarity with types of evidence	82	**4.38**	91%	0%	3	**4.38**	98%	9%	0%	N/A	N/A	Included
140	Demonstrate familiarity with scientific referencing	82	**4.20**	79%	0%	5	**4.15**	94%	7%	1%	N/A	N/A	Included
141	Demonstrate familiarity with clinical research design	82	**4.18**	79%	4%	4	**4.19**	96%	10%	2%	N/A	N/A	Included
142	Demonstrate knowledge of types of manuscripts	82	4.00	74%	7%	3	**4.04**	96%	9%	1%	N/A	N/A	Included
143	Be working towards a deeper understanding of multiple research epistemologies	80	3.35	45%	20%	8	3.36	9%	3%	3%	68	21%	Not Included
144	Demonstrate knowledge of issues related to supplemental materials for manuscripts	82	3.39	49%	22%	6	3.42	12%	3%	2%	70	31%	Not Included
145	Demonstrate the ability to work with editors of other journals	82	3.43	54%	20%	6	3.41	11%	2%	5%	69	28%	Not Included
146	Demonstrate knowledge of issues related to open data	81	3.65	60%	15%	6	3.65	12%	5%	2%	70	36%	Not Included
147	Demonstrate familiarity with referencing software	82	2.94	37%	37%	8	2.99	11%	5%	7%	70	17%	Not Included
148	Demonstrate knowledge of Digital Object Identifier assignment	79	2.70	29%	52%	13	2.83	13%	6%	10%	69	19%	Not Included
**Ethics and Integrity**													
149	Demonstrate knowledge of issues around registration (i.e., trials, systematic reviews, protocols)	80	**4.23**	86%	4%	6	**4.25**	98%	8%	0%	N/A	N/A	Included
150	Demonstrate knowledge of and adherence to the principles of editorial independence	80	**4.56**	95%	1%	3	**4.55**	99%	92%	0%	N/A	N/A	Highly Ranked
151	Demonstrate expertise in ensuring the ethical integrity of publications	80	**4.60**	96%	0%	3	**4.59**	99%	93%	0%	N/A	N/A	Highly Ranked
152	Identify and address allegations of fraud or plagiarism	80	**4.70**	98%	0%	3	**4.68**	100%	93%	0%	N/A	N/A	Highly Ranked
153	Demonstrate understanding of privacy, confidentiality, and anonymity issues	80	**4.56**	95%	0%	4	**4.55**	99%	92%	0%	N/A	N/A	Highly Ranked
154	Identify and address issues related to conflicts of interest	80	**4.58**	96%	1%	4	**4.53**	99%	91%	1%	N/A	N/A	Highly Ranked
155	Identify and address issues related to industry-sponsored research	79	**4.39**	89%	1%	3	**4.33**	95%	7%	1%	N/A	N/A	Included
156	Separate decision-making from commercial considerations	79	**4.62**	91%	1%	5	**4.55**	95%	92%	1%	N/A	N/A	Highly Ranked
157	Demonstrate knowledge of the ethical approval process for research involving humans and animals	80	**4.48**	91%	1%	4	**4.49**	99%	11%	0%	N/A	N/A	Included
158	Ensure the respect and privacy of patients described in clinical studies	79	**4.61**	94%	0%	5	**4.59**	98%	93%	0%	N/A	N/A	Highly Ranked
159	Safeguard the rights of study participants and animals	80	**4.38**	86%	4%	3	**4.38**	97%	11%	1%	N/A	N/A	Included
160	Demonstrate understanding of issues related to dual-use research (research with multiple purposes or applications)	79	3.80	70%	10%	6	3.80	13%	4%	2%	68	9%	Not Included
161	Identify and apply appropriate reporting guidelines	80	**4.41**	93%	0%	4	**4.39**	98%	7%	0%	N/A	N/A	Included
162	Guarantee access to, and long term preservation of, the published information	80	3.70	69%	18%	13	3.62	16%	6%	8%	69	19%	Not Included
163	Encourage debate on important topics related to the journal	80	3.66	61%	10%	6	3.63	10%	4%	2%	69	6%	Not Included
164	Promote higher standards of medical journalism	80	3.89	70%	10%	4	3.90	15%	8%	3%	68	6%	Not Included
165	Identify and work to avoid publication bias	78	**4.31**	91%	1%	5	**4.28**	98%	7%	1%	N/A	N/A	Included
166	Demonstrate knowledge of COPE resources for editors, authors, and peer reviewers	78	**4.13**	79%	5%	4	**4.10**	94%	6%	1%	N/A	N/A	Included
167	Demonstrate knowledge of copyright issues	79	3.87	72%	10%	8	3.88	16%	8%	2%	69	6%	Not Included
168	Demonstrate knowledge regarding problems with multiple publications (e.g., salami, duplicate, redundant)	80	**4.38**	93%	4%	4	**4.37**	98%	10%	2%	N/A	N/A	Included
169	Identify and address incongruities and bias in manuscripts	78	**4.36**	87%	1%	4	**4.31**	95%	8%	1%	N/A	N/A	Included
170	Recommend publication of papers that meet standards of scientific rigor	79	**4.42**	87%	1%	5	**4.43**	97%	12%	0%	N/A	N/A	Included
171	Identify and address issues related to image manipulation	79	3.94	66%	9%	8	3.87	13%	6%	4%	69	7%	Not Included
**Qualities and Characteristics of Editors**													
172	Demonstrate experience and broad knowledge of the field(s) covered by the journal and of the people working in those fields	80	**4.16**	86%	3%	2	**4.11**	96%	4%	1%	N/A	N/A	Included
173	Demonstrate the ability to work in a team	80	**4.20**	81%	4%	3	**4.17**	97%	6%	2%	N/A	N/A	Included
174	Delegate/divide the workload	79	**4.05**	77%	6%	5	**4.03**	97%	7%	3%	N/A	N/A	Included
175	Communicate clearly with others	80	**4.54**	93%	0%	4	**4.53**	100%	90%	0%	N/A	N/A	Highly Ranked
176	Effectively summarize manuscripts in fields outside your experience	79	3.44	47%	11%	4	3.41	7%	2%	3%	69	19%	Not Included
177	Possess a Doctorate or Master's Degree in related content area	80	3.33	50%	26%	14	3.30	15%	5%	9%	69	28%	Not Included
178	Demonstrate an academic education that includes science training or experience in a research environment	80	**4.11**	76%	4%	5	**4.09**	95%	8%	2%	N/A	N/A	Included
179	Demonstrate experience and aptitude in conflict resolution	80	3.70	60%	10%	7	3.70	15%	4%	2%	69	57%	Not Included
180	Demonstrate excellent organizational, project, and time management skills, including the ability to work under considerable time pressure	80	**4.21**	80%	1%	3	**4.22**	98%	7%	0%	N/A	N/A	Included
181	Maintain part time professional practice	80	2.65	28%	46%	10	2.64	8%	4%	13%	69	10%	Not Included
182	Maintain membership in learned societies and editing-related associations	79	3.35	52%	23%	7	3.36	13%	3%	4%	68	31%	Not Included
183	Be recognized as a distinguished scholar in one's field	80	3.29	55%	26%	12	3.21	14%	1%	10%	69	26%	Not Included
184	Maintain an active research portfolio/ is employed in a research-oriented university or institute	80	2.95	41%	41%	15	2.95	12%	5%	13%	69	16%	Not Included
185	Demonstrate past experience on an editorial board	80	3.09	39%	29%	8	2.97	9%	3%	14%	69	17%	Not Included
186	Demonstrates competence as a practitioner in his or her field	79	3.18	38%	22%	7	3.10	7%	2%	8%	69	28%	Not Included
187	Demonstrate strong interpersonal skills	80	3.84	68%	8%	5	3.80	14%	3%	2%	69	58%	Not Included
188	Demonstrate good analytical skills	80	**4.31**	89%	3%	4	**4.26**	97%	5%	1%	N/A	N/A	Included
189	Demonstrate effective critical appraisal skills	78	**4.55**	99%	1%	4	**4.55**	100%	92%	0%	N/A	N/A	Highly Ranked
190	Demonstrate the ability to achieve consensus among opinionated scientists	80	3.73	66%	9%	7	3.67	12%	2%	3%	69	45%	Not Included
191	Demonstrate leadership skills	79	3.95	73%	9%	4	3.94	15%	6%	2%	69	62%	Not Included
192	Demonstrate political and public relations sense	80	3.35	53%	21%	4	3.32	10%	2%	6%	69	26%	Not Included
193	Demonstrate self-motivation	80	**4.01**	74%	9%	3	**3.97***	94%	6%	2%	N/A	N/A	Included
194	Demonstrate enthusiasm	80	3.96	73%	9%	4	3.92	12%	7%	3%	69	57%	Not Included
195	Demonstrate tolerance and persistence	80	**4.03**	79%	10%	5	**3.98***	93%	7%	3%	N/A	N/A	Included
196	Demonstrate boldness	80	3.28	44%	23%	6	3.27	10%	2%	5%	69	22%	Not Included
197	Demonstrate independent thinking	78	**4.15**	83%	8%	5	**4.11**	95%	8%	3%	N/A	N/A	Included
198	Maintain visibility and respect among peers and in the larger scientific community	80	3.85	71%	8%	5	3.78	11%	3%	3%	69	57%	Not Included
199	Maintain rigid criteria	79	2.90	33%	34%	8	2.91	7%	3%	7%	69	13%	Not Included
200	Demonstrate the ability to perpetuate or challenge master narratives	75	3.19	43%	27%	4	3.21	8%	3%	5%	67	15%	Not Included
201	Exercise convictions with a positive attitude	77	3.62	66%	17%	6	3.59	11%	4%	6%	68	38%	Not Included
202	Demonstrate a willingness to reconsider decisions	80	**4.06**	75%	3%	5	**4.03**	95%	5%	2%	N/A	N/A	Included
203	Demonstrate practicality	80	3.88	71%	9%	4	3.87	14%	5%	1%	67	49%	Not Included
204	Demonstrate decisiveness	80	**4.08**	79%	3%	3	**4.04**	94%	4%	0%	N/A	N/A	Included
205	Demonstrate personal interest in medical “journalology” or “editology”	80	3.45	55%	23%	6	3.45	10%	4%	3%	68	46%	Not Included
206	Demonstrate an enjoyment of learning and a have questioning mind	79	**4.04**	78%	9%	5	**3.99***	92%	7%	3%	N/A	N/A	Included
207	Demonstrate the desire to advance his or her field of study	80	3.95	78%	8%	5	3.91	14%	4%	2%	69	59%	Not Included
208	Have access to a good academic network or have the potential to grow one	79	3.90	75%	8%	5	3.83	13%	3%	3%	69	57%	Not Included
209	Demonstrate patience when dealing with authors and reviewers	80	**4.24**	84%	1%	5	**4.25**	96%	10%	0%	N/A	N/A	Included
210	Demonstrate knowledge of processes related to the editorial board	79	**4.23**	81%	5%	4	**4.20**	95%	7%	0%	N/A	N/A	Included
211	Respond promptly to complaints	80	**4.34**	89%	0%	3	**4.35**	99%	8%	0%	N/A	N/A	Included
212	Act with integrity and accountability	79	**4.84**	100%	0%	4	**4.82**	99%	96%	0%	N/A	N/A	Highly Ranked
213	Demonstrate managerial skills	79	3.84	71%	14%	7	3.86	18%	6%	2%	69	57%	Not Included
214	Demonstrate the ability to link journal content to current affairs	80	3.38	54%	24%	7	3.36	10%	3%	5%	69	20%	Not Included

Items Suggested by Participants in Round 1 that Did Not Reach Consensus in Round 2

Item #	New Competency-Related Statements	# Respondents for New Items for Round 2[12]	Round 2 Average Score[8]	% Agreement for ≥4[4]	% Agreement for ≤2[5]	% Choosing "Essential"[14]	# Respondents for Round 3[15]	Final Status[16]
215	Able to asses Able to assess appropriateness of research methods in relation to the stated research question	55	**4.18**	89%	0.00	N/A	N/A	Included
216	Able to asses Able to assess validity of conclusions in relation to the research findings	55	**4.38**	91%	0.00	N/A	N/A	Included
217	Understanding of issues relating to reproducibility of research	55	3.78	65%	0.04	68.1%	69%	Not Included
218	Understanding of issues relating to selective outcome reporting	55	**4.00**	78%	0.00	N/A	N/A	Included
219	Ability to detect conflicts that have not reported by authors, and deal with these appropriately	54	3.65	61%	0.07	72.5%	69%	Not Included
220	Having expertise in how to correct the published record	55	3.49	49%	0.15	40.6%	69%	Not Included
221	An understanding of different peer review models to improve efficiency of reviewing processes, such as cascading	55	3.29	40%	0.18	29.0%	69%	Not Included
222	Understand impact factor and how citations can help with impact factor	55	3.36	44%	0.16	42.0%	69%	Not Included
223	Good comma Good command of Scientific English	55	**4.16**	80%	0.04	N/A	N/A	Included
224	Engage with copy editors	54	3.43	48%	0.20	36.8%	68%	Not Included
225	Ability and willingness to arrange regular editorial meetings to discuss matters arising	54	3.72	61%	0.09	65.2%	69%	Not Included
226	Ability to check that you are making consistent and transparent decisions	55	**4.04**	82%	0.02	N/A	N/A	Included
227	Involve patients, research subjects and communities in journal policy	55	3.07	33%	0.33	14.5%	69%	Not Included
228	Awareness of cultural differences	55	3.53	53%	0.15	26.1%	69%	Not Included
229	Ability to hand over his/her tasks and duties to the next scientific editor	55	3.56	55%	0.07	50.0%	68%	Not Included
230	Language competency particularly from a country where English is not the native-tongue	55	3.84	71%	0.11	66.2%	68%	Not Included

*These (5) items were ranked above 4.0 in Round 1 and ranked below a 4.0 in Round 2 but were inadvertently left out of the rating for Round 3.

1. Round 1. Items in Round 1 were ranked on a scale of 1 (not at all important) to 5 (absolutely essential).

2. # of Participants for Round 1. This is the number of participants who provided an answer for the specific item

3. Round 1 Average Score. For each item, the total of all scores was calculated and divided by the number of participants who included a score for the item.

4. % Agreement for ≥4. For each item, the number of participants scoring a 4 or higher was divided by the number of participants who included a score for the item.

A yellow highlight indicates that 80% or more of the participants scored a 4 or higher for the item

	A green highlight indicates that 90% or more of the participants scored a 4 or higher for the item
5. % Agreement for ≤2. For each item, the number of participants scoring a 2 or lower was divided by the total number of participants who included a score for the item	
	A purple highlight indicates that 80% or more of the participants scored a 2 or lower for the item (there were no instances of this occurring in any round)
6. Round 2. All items from Round 1 were included, in addition to new items suggested in Round 1. If an answer was not provided for a specific item from Round 1 (but the participant had answers for any other Round 2 items), it was assumed (as per the instructions) that the participant ageed to the average score from Round 1 for that item and therefore the average score was used. Participants socred the new items using the same 5-point scoring system used in Round 1.	
7. # of Participants That Made Changes in Round 2. This is the number of participants that did not agree with the score of the particular item and included a different score.	
8. Round 2 Average Score. For each item the total of all scores was calculated and divided by the total number of respondents to the Round 2 survey.	
9. % Agreement for ≥4. For each item, the number of participants scoring a 4 or higher was divided by the number of participants who participated in Round 2.	
	A yellow highlight indicates that 80% or more of the participants scored a 4 or higher for the item
	A green highlight indicates that 90% or more of the participants scored a 4 or higher for the item
10. % Agreement for ≥4.5. For each item, the number of participants scoring a 4.5 or higher was divided by the number of participants who participated in Round 2.	
	An orange highlight indicates that 80% or more of the participants scored a 4.5 or higher for the item
	A blue highlight indicates that 90% or more of the participants scored a 4.5 or higher for the item
11. % Agreement for ≤2. For each item, the number of participants scoring a 2 or lower was divided by the total number of participants who participated in Round 2.	
	A purple highlight indicates that 80% or more of the participants scored a 2 or lower for the item (there were no instances of this occurring in any round)
12. # Participants for New Items for Round 2. This is the number of participants who provided an answer for the specific item.	
13. Round 3. In this round, only items not reaching consensus (i.e., 80% agreement of ≥4 or ≤2) were included. Partricipants rated these items on a 3-point rating scale from 1 (Not at all important) to 3 (Absolutely Essential).	
14. # of Participants for Round 3. This is the number of participants who provided an answer for the specific item.	
15. % Choosing "Essential" Option. For each item, the number of participants choosing the "Absolutely Essential" option was divided by the number of participants who responded to the item (No new items reached consensus in Round 3).	
16. Final Status. Each item is listed as "Included" or "Not Included" based on the final Average Score and % Agreement for the item.	
	A red highlight indicates that 90% or more of the participants scored a 4.5 or higher for the item. The final status of this item is "Highly Ranked".
	A pink highlight indicates that 80% or more of the participants scored a 4 or higher for the item. The final status of this item is " Included".
