# Supplementary material for: An international survey and modified Delphi process revealed editors’ perceptions, training needs, and ratings of competency-related statements for the development of core competencies for scientific editors of biomedical journals
Source: F1000Res. 2017 Sep 4;6:1634. [Version 1] doi: 10.12688/f1000research.12400.1 (PMC5605946; doi:10.12688/f1000research.12400.1)
Supplement: Supplementary file 3 [file f1000research-6-13429-s0002.tgz › cafac36d-8372-4570-a7c5-2625bc1c2756.docx]

**APPENDIX A - Questions for survey of editors’ perceptions**

This is a list of all of the questions that were asked in the needs assessment questionnaire.

1. What is your e-mail address? Your e-mail address is necessary in order to contact the winner of the draw prize for participation (an iPad Mini) and for inclusion in the Delphi process which will be conducted in the near future.
2. From which organization did you obtain the link to this survey?
3. What is your job title at your journal?
4. What is your job title outside of your work at your journal?
5. What is your sex?
6. What is your age?
7. What is the highest level of education obtained (MD or other health professional degree may be checked in conjunction with another degree)?
8. How long have you been working as a scientific editor (e.g., Associate Editor, Editor-in-Chief)?
9. What type of journal do you currently work at?
10. Where is the journal located?
11. What is the reach of your journal?
12. How many articles (all article types) did your journal publish in 2014?
13. Is the journal where you work a member of the Committee on Publication Ethics (COPE)?
14. Are you a member of any of the following editorial organizations (Check all that apply):
15. Please give details on the different scientific (i.e., decision-making) editorial positions you've held (i.e., job title and number of years)
16. Has the current journal for which you work provided you with FORMAL or INFORMAL training related to your work as a scientific editor? Please describe.
17. Have you acquired any other FORMAL training related to scientific editing of a journal (e.g., workshops, courses, formal mentoring) beyond or before what was provided at your current journal? Please describe.
18. Have you acquired any INFORMAL training or knowledge related to scientific editing of a journal (e.g., books, websites, informal mentoring) beyond or before what was provided at your current journal? Please describe.
19. Do you have any FORMAL or INFORMAL training in research methods? Please describe.
20. Do you have any FORMAL or INFORMAL training in statistics? Please describe.
21. Please rate THE IMPORTANCE of the following expertise-related items to the performance of your job as editor (18 items to rate).
22. Please rate THE IMPORTANCE of the following skills and experience to the performance of your job as editor (20 items to rate).
23. Please rate HOW MUCH YOU POSSESS the following expertise in your job as editor (18 items to rate).
24. Please rate HOW WELL YOU PERFORM the following skills or HOW MUCH YOU POSSESS the following experience in your job as editor (20 items to rate).
25. Please specify the areas of your work as an editor in which you would like to receive further training or instruction. List these in order of importance (#1 is most important):
26. Would you be interested in participating in an online Delphi process to help develop a minimum set of core competencies for scientific editors of biomedical journals? The Delphi will involve a large group of scientific editors who anonymously reply to a questionnaire about editor competencies and subsequently receive feedback in the form of a statistical representation of the "group response," after which the process will repeat itself two more times (for a total of 3 questionnaires). The goal is to reduce the range of responses and arrive at something closer to expert consensus on core competencies for scientific editors.
