# Supplementary material for: An international survey and modified Delphi process revealed editors’ perceptions, training needs, and ratings of competency-related statements for the development of core competencies for scientific editors of biomedical journals
Source: F1000Res. 2017 Sep 4;6:1634. [Version 1] doi: 10.12688/f1000research.12400.1 (PMC5605946; doi:10.12688/f1000research.12400.1)
Supplement: Supplementary file 4 [file f1000research-6-13429-s0003.tgz › 951d66dd-5ff9-4e1b-b180-3268a2efa7c6.docx]

**APPENDIX B - Text answers for survey of editors’ perceptions**

**TEXT ANSWERS FOR QUESTION #16: *Has the current journal for which you work provided you with FORMAL or INFORMAL training related to your work as a scientific editor? Please describe.***

- These courses are provided by the publication office of our institute as in Iran medical journals are mainly owned by medical institutes. I mean the courses are not designed directly by our journal itself. 1. working with our journal online system: 8 hours (1 workshops), 3 formal mentoring (9 hours) 2. medical journalism: 72 hours (various workshops), 2 formal mentoring (4 hours) 3. ethics in medical publications: 10 hours (2 workshops), 2 formal mentoring (3 hours) 4. manuscript reviewing: 53 hours (various workshops), 1 formal mentoring (2 hours) 5. manuscript preparation: 14 hours (2 workshops), 1 formal mentoring (2 hours)
- Most has been learn by doing and from other editors. There have been numerous formal educational sessions on all aspects of editing and quality of reporting.
- Training in the usage of the Journal management system (ScholarOne) by Sage publisher which is the Journal publisher
- Mentoring from supervisor
- Participation in National and Editorial and scholarly publication workshops
- I attended in different work shops in the field of journalism, how to review the article?, scientific writing, ...
- At my current journal, no. In previous positions, yes. My first year at The BMJ I was editorial registrar, a fellow position that involved training and apprenticeship. This was the first year of my professional career as a journal editor.
- I was aware of research and publication ethics during my post-graduation and the 7 years when I taught at a medical school. Working for the journal gave me a behind the scenes view of the detailed publication processes and a senior editor provided mentorship for the first 18 months. I was encouraged to take online modules (COPE) and participate in WAME discussions.
- My journal "The New Iraqi Journal of Medicine "was stopped but I am provider of training for medical editors; conducting medical editor courses based on books I published in the field
- Elsevier publishing campus, Netherland-2015 Preparing your manuscript How do Editors look at your paper? Step-by-step guide to reviewing The journal publishing cycle Peer review in Asia: special tips for Reviewers Plagiarism Authorship and responsibilities. 2. Springer- Journal Author Academy- 2015 Writing your Manuscript Submitting and Peer review How to Write and Submit a Journal Article.
- BMC provides information for journal editors.
- Informal throught modules and pdf info
- informal orientation from previous editor
- ...But as EIC I have provided others with some basic training materials both through the publisher (generic from Wiley) and individually on publication ethics and COI for reviewers and editors.
- They do for more junior editors.
- workshops on different aspect of research methodology, review process, statistics etc
- I received informal training from the proviso editor-in-chief over an overlap period of 6 months.
- Workshop by a senior member of the editorial board.
- Through nomination to WHO regional TOT in scientific publication 2009
- I was nominated to attend a workshop that was arranged by an International Organization to support local scientific journals and the trainer was a member of WAME. Later, the publisher provided short workshops to the editors.
- No training
- electronic file was sent which contained how to use editorial and managerial software.
- Attended number of workshops and conferences on medical writing , publication ,research and ethics.
- work with experienced editors
- Short Course for Editors of Medical Journals, Oxford UK
- I've improved my editing, reviewing and academic writing skills by following discussions on WAME's Listserve forum since 2008, attending and lecturing at numerous science writing, editing and communication seminars and conferences since 2009, and by managing the editing process in several biomedical journals indexed by Scopus and Web of Science. Besides, I've acquired essential skills in ethical peer review by reviewing about 1000 submissions to numerous indexed biomedical journals (since 2006).
- some workshop by Elsevier publisher
- The journal provides new editors with manuals on tasks of each editor and toubleshooting of possible problems. - Skype meeting for new editors. - Following up on the work of editors every 3 months.
- I'm a doctor and I studied the masters in health sciences with concentration area in epidemiology (1998-2000) as well as various courses on writing scientific papers (on and off), in order to better do editorial work.
- we have attended EASE conference and we received information from EASE
- Only with encouragement and support to attend short courses for new editors; encouragement and support to be a member of and attend annual meetings of several editorial societies; mentoring by Editor-in-Chief at the time for my single journal; trial and learning by experience.
- I have been provided with training at coferences and at the workshops. EASE conference 2014 Split - Pippa Smart workshop for editors Workshop for editors on publication ethics in Split 2012., organized by Ana Marusic
- Guideance rather than training are available. Network of research method experts to consult, meetings and handbook provide information resources to use.
- Informal and formal attendance at workshops run by the Cochrane Collaboration
- Feedback from the Editor in Chief
- Cochrane Review author workshops Scientific/methodology workshops during Cochrane meetings
- Training in use of the online systems; no training in editorial process
- Mentorship with another Editor Handbook
- Informal inhouse training on Editorial processes.
- Formal initial 'buddy' system where I co-reviewed my first manuscript with a senior editor. Informal training through access to organisational methods guidance and publishing policies. Informal training through access to organisational quality assurance findings identifying common errors. Formal feedback from organisational quality assurance process on manuscripts I have edited. Informal training through shared input into manuscripts by statistical editors.
- Only on how to work the journal system although there is informal mentoring from the Editor-in-Chief.
- I am based at the London School of Hygiene and Tropical Medicine, and regularly attend lectures on all areas of science out of personal interest - probably three lectures a week on average. I have received training from Cochrane in relation to my work. My Cochrane Group supported me to do a MSc in Epidemiology, which I did part-time alongside working part-time.
- Training in form of videos and text about publication criteria, how to find reviewers, where to ask for help, how to navigate the submission site and responsibilities as an Academic/ Section Editor. Also, links to discussion forums.
- Mentorship: checking work, providing comments and supervision, assuring quality of work Informal one-to-one training on statistics in systematic reviews Cochrane colloquia workshops (e.g. peer review workshop, understanding risk) Techology training for editing software
- Orientation documents and videos.
- Online seminar
- Informal training takes place during annual editorial board meetings
- Mentoring, probation (decisions reviewed in first year)
- Webinars and formal didactic training.
- Attended a 4-day "Training of trainers on medical journal publishing" course run by psp consulting in 2009 Attended a 5-day Training of trainers course on Writing a scientific paper, and getting it published run by Tim Albert
- Workshops Atypical exams through reviews be fore entering the editorial-board
- Provided powerpoint presentations regarding how to handle manuscripts, how to find expert referees, how to take decisions.
- PLOS ONE has had the greatest level of training (formal), and I have also mentored/advised several other academic editors as they struggle with decision making.
- Informal and ongoing online training
- Seminars in the annual Cochrane Colloquium, and Courses at the UK Cochrane Center though both a very expensive to attend.
- Informal training with videos and offers of webinars, on technical issues regarding new web-based editorial process.
- On-the-job training and I have been able to attend numerous training events/workshops and conferences.
- Workshops at which common errors are presented and discussed. Annual meetings of editors to discuss issues, which have included presentations on specific methodological issues. Online modules stepping through new requirements for publications, how to approach and assess these.
- Informal.
- Informal: two days of mentoring from an experienced managing editor in my first three months. Workshops and seminars on relevant topics sometimes available at national and international meetings (eg UK Symposium and Colloquium) however programmes usually aimed at authors. Training available online for Cochrane authors, which can be utilised by editorial staff, but nothing specific for MEs (Managing Editors). The ME community is a welcoming and friendly group, and I have gratefully received advice and support from my peers. There is an
- ME forum via the Cochrane community website which offers the opportunity to share challenges, experiences and guidance. Each of the 53 review groups has developed their own sets of guidance due to slightly different practices. Although there is a template workflow in Archie, this is not mandated and groups do not necessarily utilise it or follow it in task order.
- Both. Informal training both methodological and related to editorial processes. This is ongoing and extensive via Cochrane Colloquia, mid year meetings, courses. Focus largely on the science but also extensive policy conversations e.g., regarding conflicts, peer review, editorial processes. Ongoing peer support from fellow Coordinating Editors and Central Editorial Unit invaluable.
- The journal has invited me for the training but I could not attend due to the financial constraints.
- One-on-one and group discussion with senior editorial staff
- Formal workshop training Informal peer led training
- POST GRADUATING TRAINING 1992 International Agency for Research on Cancer. European Educational Programme in Epidemiology. 1993 McMaster University. Department of Clinical Epidemiology e Biostatistics. "Workshop on how to teach the Critical Appraisal of Clinical Evidence". Hamilton, Canada, 1993 Biometric Society. "Seminars on regression models in Epidemiology. Analisis of correlated data". Florence University . 1995 Azienda Agraria Sperimentale “Vittorio Tadini”. Statistic methodology, Advanced course 1997 Italian Association of Epidemiology . Master in Epidemiology. 1998 - Seminar “Evidence based medicine”.CEFPAS Caltanissetta, Italy 2000 Intensive Course on Methodology of Systematic Reviews. Italian Cochrane Centre , Modena 2005. Workshop II° level: “Systematic reviews of diagnostic accuracy” Italian Cochrane centre, Milan 2006 Workshop III° level: “Systematic reviews of diagnostic accuracy” Italian Cochrane centre, Milan 2009 Continental Europe Regional Support Unit (CESU) of the Cochrane Diagnostic Test Accuracy Working Group Attended regularly at the Cochrane Colloquium since 2005, followed all the workshops related to risk of bias assessment for RCTs and observational studies, GRADE, meta-analysis, Diagnostic accuracy, common error in SRs
- Cochrane editorial training
- My on boarding education was thorough and experiential. The outgoing editorial team and the incoming team (myself and 3 associate editors) worked together for 6 months before we officially assumed the editorial positions. This allowed us to do the work under expert mentoring. The editorial staff remained stable and excellent and also provided informal training (introduction to WAME, alert to attend Peer Review Congress, COPE, etc) There were no formal courses or recommendations for articles to read or other resources. I feel like in general this was a good process but I do wish that there was a more formal library (other than what is on equator-network to assist the incoming editors.
- Information about editing, publishing, policies as well as mentoring provided by senior collagues
- Informal support and guidance is ongoing from the CSG
- Workshop on how to review a paper
- Cochrane organises regular training events that are occasionally targetted to editors and I had the chance to attend these. I have also worked for Cochrane as a training co-ordinator while I was serving as an editor and within that unique framework I started putting together training materials for editors.
- I had to attend courses conducted on ethics,publication process,plagiarism(Malaysian Journal of Medical Sciences).I was an Associate Editor from 1995 till 2007 before I became Chief Editor in 2008.I am on the animal ethics committee of the Universiti Sains Malaysia for the last 10 years as well as was on the Human Ethics Committee for 9 years
- Extensive training over months when I joined. Usually conducted as 1-2 hour presentation followed by handling work with oversight and becoming progressively independent. Publication ethics (plagiarism, image manipulation, dual pub, copyright etc), research ethics (animal, human, and field studies), reporting guidelines for various study types. We also have weekly meetings to discuss difficult cases, which function as ongoing training.
- First position was manuscript editor (minimum six months on-the-job training); manuscript editing supervisor (no training except for association-sponsored workshops); assistant, associate, and associate senior: on the job training, courses in CSE and AMWA.
- Informal training: mentoring, manuscript discussions. Formal training: relevant seminars held at my workplace.
- Attended Workshop organised by The BMJ for Local Editors and Publishers of its Local editions in 1995-1997 in Athens, Greece; London UK; Manipal, India. Been convener and participant in annual Editors workshops from 2000 - 2014 in Nigeria, Ghana organised by the BMJ West Africa Local Edition.
- When hired I received 6 months of informal training by the editor in chief consisting of him going through my manuscripts and approving my peer review decisions. After 6 months he continued to read through my edited manuscripts but no longer approved peer review decisions. Our journals have never had any formal training but I did institute the first formal CME via editors completing the Statistics in Medicine MOOC run by a friend/colleague at Stanford. In terms of ethics issues and handling of those we follow the recommendations of COPE but unfortunately do not belong. I submitted to the last PRC but this was the first such submission from an editor of this journal.
- Training occurred after being editor through workshops and seminars organized by the academy for scientific research and technology in collaboration with WHO EMR Elsevier and Lippincott participated in running these activities Participating in conferences held by EMAME and WAME and some of our Egyptian universities
- Informal.
- CSE EIC training On the job training.
- Mix of formal and informal on the job type training
- Annual awaydays Also I trained editors for many years
- Informal training over the years including membership on the Editorial Board and Annual Editorial Board meeting for three days.
- I had a informal training about the open journal systems and desktop publishing softwares.
- attended workshops organized by indian association of medical journal editors and reviewers , indian journal of pharmacology , at JIPMER pondicherry
- Session at WAME conference.
- Informal - not through the journal
- Informal training through Elsevier online modules
- I created the training programs
- Informal, on the job training plus participation in CSE meeting
- Shadowed editor in chief for 6 months following my appointment as Editor-elect
- 6-hour training workshop that covered various topics related to ethics in publishing, editorial responsibilities, good writing principles, etc.
- But I provided a one-day workshop for Campbell Editors on 3 October 2015. This covered publication ethics, editors' role, decision making, scientific writing, COPE standards

**TEXT ANSWERS FOR QUESTION #17: *Have you acquired any other FORMAL training related to scientific editing of a journal (e.g., workshops, courses, formal mentoring) beyond or before what was provided at your current journal? Please describe.***

- I am self-motivated and I try to attend the courses as I feel need for them. I have attended various national and international courses about medical journalism, ethics in medical publications, manuscript reviewing, manuscript preparation, in person or online ... 1. working with our journal online system: 5 formal mentoring (9 hours) 2. medical journalism: 12 hours (2 workshops), 5 formal mentoring (5 hours) 3. ethics in medical publications: 8 hours (1 workshops), 4 formal mentoring (5 hours) 4. manuscript reviewing: 6 hours (1 workshops), 4 formal mentoring (6 hours) 5. manuscript preparation: 24 hours (3 workshops), 1 formal mentoring (2 hours)
- Annual meetings of CSE, SSP, EASE, and AMWA - too many years and occurrences to specify Workshops at CSE EQUATOR workshop Several Evidence-Based Medicine workshops Stanford Professional Publishing Course (now managed by Yale)
- Yes, attending workshops run by the European Council of Science Editors ( a week) Others-- Reviewing workshops ( 3 days)
- National and Editorial and scholarly publication workshops - 2 weeks, 2007
- About 4-5 meeetings lasting from two to three days, on methodological issues related to editing, between 2007 and 2014.
- I attended in different work shops in the field of journalism, how to review the article?, scientific writing, ...
- (From my CV): Council of Science Editors’ Course on Journal Editing, Pittsburgh USA, 2-3 May 2003 BMJ Short Course for Medical Editors, Barcelona Spain, 12-16 September 2001 (Sponsored scholar) Medicine in the Media: Reporting on Medical Research course, National Institutes of Health, Washington DC,14-17 Oct 2012. Accredited Trainer for “Writing a Journal Article and Getting it Published” course (since 2007). Tim Albert Training UK. Effective Writing and How to Write a Scientific Paper, Tim Albert Training London UK, Mar 2003 Mediterranean Summer School on Biomedical Research Methods, Catania Sicily, 2-8 June 2003.
- 1.Elsevier -Editors update Webinar training 2. International society of Physiotherapy Journal Editors- Webinars training
- I did a two day editor training course in Christchurch, New Zealand, 5-6 August 2004. (Short Course for Editors of Medical Journals, Tim Albert Training)
- Workshop on scientific writing for publication. 4 hours. 1995. Workshop on "Open Journal System". One day. 2010.
- With Latindex 6 meses months program
- I am Reserach in reproductive system and passed many workshop By american society of andrology as i am active member of this society since 2005
- Publisher Workshop; WAME meeting.
- Workshop at Peer Review Congress
- I was formally trained at the Ciba Foundation in manuscript editing.
- Conferences of EMAME Conferences of Pakistan Association of Medical Editors Workshops of Pakistan Association of Medical Editors
- Workshop by National Association of Medical Editors
- Participation in national conferences for scientific editing: 32 hours (total), 2012, 2013-2014-2015 Participation in training courses for scientific editing: 40 hours (total), 2012, 2013-2014-2015
- Online publisher webinars and self-learning materials were provided by the journals for which I have reviewed manuscripts.
- I have attended meetings EASE practically on many occasions.
- participation in workshops, conferneces on editorship and related matters
- Scientific Writing Course as part of my teaching certificate
- Seminars in science writing and editing arranged by European professional associations - each ranging from 6 to 36 academic hours.
- Many courses and workshops by my affiliated university
- Workshop attended EASE conference in Talline
- During my MSc I received training in statistical analysis, qualitative and quantitative synthesis and user engagement/participation in research
- I got a master degree in Epidemiology, part-time, between 2009-2011.
- Standard English course (2004) Copy-editing course (2004) Formal mentoring (2004) News writing (2005) Journal Marketing (2006) How to Practice Evidence-Based Health Care, ran by the Oxford Centre for Evidence Based Medicine (2009) Editor-led training meetings, covering topics such as equivalence and non-inferiority (2009/2010)
- Am a Board of Editors in the Life Sciences (BELS) certified Editor in Life Sciences (ELS)
- 2 workshops
- As an editor as J Biol Chem, I received training (presentations/discussion) regarding how to handle manuscript (1 day - 2013)
- I did a Masters in Public Health and am currently doing a PhD.
- Cochrane Courses and training in DTA reviews editing process
- .
- 1-day Critical appraisal workshop - 2004 Attendance to COPE forums and seminar 204-2005 Internally run workshops on critical appraisal, qualitative research (at previous position)
- Workshop on how to review a paper
- I've been to a COPE symposium.
- Biostatistics courses during MD program (late 1960s) and later, during employment years (early 1980s?). Many workshops, mainly at conferences, during employment years (early 1980s to late 1990s).
- Attended Workshop organised by The BMJ for Local Editors and Publishers of its Local editions in 1995-1997 in Athens, Greece; London UK; Manipal, India. Been convener and participant in annual Editors workshops from 2000 - 2014 in Nigeria, Ghana organised by the BMJ West Africa Local Edition.
- See above, the Stanford MOOC. And for me personally via my relationships with colleagues at Ann Int Med in particular I have received much personal mentoring, also help from friends at Equator Network. I networked extensively building relationships which have yielded relationships that have led to collaborations for research as well as much benefit to me personally. This for me occurred as I worked to improve the quality of reporting as well as efficiency of our manuscript handling processes. I started about a year after I was hired.
- Workshop, 2012, one week
- Similar to those mentioned in the above section each for 3 days
- A number of courses and informal meetings over the years.
- I have joined some workshop and courses about scientific editing.
- by indian association of medical journal editors and reviewers as part of continuing medical education programmes
- Institutional presentations on various topics related to scientific literature.
- Workshop on editorial writing BMJ maybe 15 years ago
- Lantidex training
- I participated in a short course for medical journal editors run by Pippa Smart in Oxford (UK) in November 2009
- CSE new editor course 2002 - 3 days BMJ Internship - 2002 - 2 weeks EBM and clinical epic training - various
- through conducting trainings for author, reviewer, editors by name- nepal association of medical editors of which i am vice president
- I have a Masters degree in quantitative methods, but this was not specifically focused on editinga nd was obtained prior to my becomng an editor, I also have an undergraduate degree in writing
- Attended workshops on scientific writing skills

**TEXT ANSWERS FOR QUESTION #18: *Have you acquired any INFORMAL training or knowledge related to scientific editing of a journal (e.g., books, websites, informal mentoring) beyond or before what was provided at your current journal? Please describe.***

- I usually review COPE website about ethics and other topics, I discuss the problems I face with the editors of higher ranked journals and I review the review by our journal reviewers and discuss their comments before passing it to the authors
- 4 years are writing articles 1 year of persuasive writing 1 year of biostats
- Yes - working with publishing partners.
- Membership of WAME, past 18 years Reading journalology articles and blogs, past 20 years Learning AMA style guide and updates, past 20 years
- Online resources from WAME, AJOL, COPE
- Knowledge gained from my predecessor, from the Editor-in-Chief of a "sister"-journal, from the publishers that the journal has had and books, largely between and including 2007 and 2014.
- I read the book, articles, etc
- - Continual mentoring by senior editors and editors in chief throughout my career (including a monthly skype call with Richard Smith, my formal mentor) - Mentoring by Tim Albert, professional trainer of medical journal editors, for the first half of my career - Participation in CSE, COPE and WAME meetings and intermittent service on related commmittees - WAME listserve
- During the three years of my postgraduation (MD) I received informal training of medical writing, editing and publication processes from my mentors (i.e., department faculty and senior colleagues)
- 1. How to get your book published- July 2015. 2. Why write a book- July, 2015
- Diploma in clinical research and medical writing
- I have attended three COPE seminars (Washington Nov 2009, Melbourne 2013 and 2014).
- Books (in English and/or Spanish); papers in different journals (in English, Spanish, Portuguese); WAME and other websites. 1974-2015.
- Through online module and coursera courses
- attend conference sponsored by International Association of Nursing Editors read books- Writing for Publication reviewed COPE standards participate in editor discussion boards subscribe to Nurse Author and Editor
- Books, websites, informal mentoring have all been used as well as literature reviews on publication-related topics and statistics, as well as books and publications on the proper use of the English language. The details over the past decade would be difficult to recall.
- Books, Websites and Conferences
- Conferences, particularly EASE conferences. Reading European Science Editing, The Scholarly Kitchen blog and various other publications.
- In house training by peer editors
- Through previous editors
- Study of scientific editing papers, books. 2012, 2013-2014-2015 Browsing the internet for training material from publishers (Elsevier, Emerald), 2012, 2013-2014-2015
- I attended a workshop that was part of the scientific activity of my department, by a member of WAME. I was nominated by the EiC to attend a workshop on manuscript review arranged by the university where I work.
- participation in editorial committees, write instructions for authors, translation of relevant papers in editorship, drafting editorial guidelines, teaching for young editors, use of online selected material
- Books: Scientific Editor's Handbook two more Websites: COPE Equator Network
- many training courses from 1991 till now
- Online course on performing reviews and meta-analysis.
- I have attended national conferences of the Association of publishers to which I belong, to international conferences organized by WAME, and Peer review meetings also. As an autodidact, I read books and articles related to scientific publishing.
- From a couple of books in the 1980s: Bob Day's How to write and publish a scientific paper; Claude Bishop's How to edit a scientific journal.
- guides for paper reviewing, examples of handling manuscripts where there are problems, feedback from senior editors.
- Reading of articles and books on editing and philosophical decision making. Discussions with editors and editors in chief of my primary and other journals.
- Attended meetings with talks by journal editors and general discussion
- Some informal reading on publication ethics. Informal discussions with colleagues on publication ethics.
- From the Editor-in-Chief of the journal where i am Associate Editor. This is done very sensitively but is sporadic depending on what is going on that needs me to be prompted on. I have read a lot and keep an eye on the COPE website - I sometimes go there for advice. I also attended the recent EQUATOR REWARD conference where some of these issues were discussed and there was informal discussion.
- Our editorial team is based at a large research university (London School of Hygiene and Tropical Medicine), and approximately three times a week I attend lectures on a range of subjects related to science - everything from mathematical modelling to infectious disease epidemiology. On my own time, I enrol in free online courses in science through Futurelearn (https://www.futurelearn.com/).
- Mentored by senior editors
- I co-edited two books (2002 and 2007) before I became Academic Editor and learned a lot about proof-reading, arranging chapters and chasing up contributors.
- On-the-job training and informal mentoring for all roles has formed the majority of training I've received, and has been the most valuable.
- Experience.
- Many years of reviewing and editing manuscripts in my own lab and in my research division.
- 3 years
- I am a professional (ASA accredited) statistician, and a research methodologist, so I read and critically evaluate research methods, and the extent(s) to which analysis is appropriate and results support conclusions *for a living* as a scientist and professor. This is based on expertise and formal training (2 PhDs and a doctoral-level certificate; two master's degrees) accrued over about 19 years.
- Have been publishing my own research for a number of years.
- I read articles on relevant methodological and statistical matters, including how to present material in publications.
- My training is in MBE in several courses. It helps for editorial issues
- Cochrane meetings
- informal mentoring adhoc over 15 years.
- Since 2011 I have attended workshops at Cochrane Colloquiums and related events regarding peer review and content of Cochrane reviews
- MSc in Evidence Based Healthcare from University College London, less than one year
- reading books (the best is Richerd Smith's one) and articles
- Trish Greenhalgh's "How to read a paper" series and other articles about appraising research COPE and WAME websites COPE forums and seminars
- Yes, as a referee: 2005 to present
- Cope Publisher online resiurces
- .
- 1. I read the info on Equator-network for editors 2. I've taken an on line course from Bloomberg School on Coursera that was a 6 weeks course on systematic reviews, an area of publication that I was unfamiliar with. 3. When I find an interesting article, I forward that to my staff and associate editors. 4. One resource is the weekend reads from Retraction Watch--these sometimes include some fascinating and varied articles.
- COPE newsletter (since 2014)
- Have attended meetings and discussions at University of Cape Town
- Went along to workshops and presentations offered in my academic institution.
- I have been a systematic reviewer for may years and published over 30 systematic reviews
- yes,I have attended workshops on editing organized by Universiti Sains Malaysia once a year 1 day exposure for the last 4 years
- Reading COPE website.
- Workshops through professional organizations
- Books written mainly by experienced scientific editors
- Attended Workshop organised by The BMJ for Local Editors and Publishers of its Local editions in 1995-1997 in Athens, Greece; London UK; Manipal, India. Been convener and participant in annual Editors workshops from 2000 - 2014 in Nigeria, Ghana organised by the BMJ West Africa Local Edition.
- Please see above.
- websites (specially those of COPE
- I a a researcher and author.
- Serving as a peer reviewer for multiple journals, being mentored by other journal editors and senior scientific editors.
- In running training courses for editors, as an ex chair of C OPE, contributor to CSE and EASE publications and as a lecturer on editorial topics, I have thereby learned much from colleagues. Also am now chair of directors of a journal publisher.
- Yes - all of the above.
- workshops at editorial conferences and peer review conference
- https://pkp.sfu.ca/ojs/ http://www.councilscienceeditors.org/
- I learned the related information by reading books, journals and other published materials about the topic. Also by personal contacts with other editors.
- acquired informal training from syosys technologies in the fields of Web based Solutions, Responsive Websites & Website design, Online strategy development etc .
- Session at WAME conference.
- Informal mentoring previous EIC CMAJ 20 years ago
- through editorial courses online
- Editorial board training on past journals
- through conducting trainings for author, reviewer, editors by name- nepal association of medical editors of which i am vice president
- Networking with colleagues at peer journals
- Informal mentoring when colleagues had editorial positions and I was learning how to be a reviewer.
- Informal mentoring on peer review processes (how to give feedback effectively) from my PhD advisor. Read books on scientific writing.

**TEXT ANSWERS FOR QUESTION #19: *Do you have any FORMAL or INFORMAL training in research methods? Please describe.***

- 334 hours since 2006 when I started my MSc: 150 hours for statistics, 15 hours for scientific search in medical databases, 54 hours for research methods, 34 hours for proposal preparation, 34 hours for manuscript preparation, 8 hours for oral and poster presentation, 34 hours for manuscript reviewing, critical appraisal , 5 hours for journal club management,....
- 3 months Organic Synthesis research 9 months Stem Cell Biology research 1.5 years of medical database research 6 months of spectroscopy research
- Workshop on research methods and quality or reporting. Reading of Users Guides to the Medical Literature and How to Report Statistics in Medicine
- Very extensive. Prominent author; over 400 peer reviewed publications
- (1) During the course of all my degree courses (Bachelor degree, Master degree and PhD course) (2) Attended workshops on Research Methods both in Quantitative and Qualitative Research Methods, in and out of the country
- Reading, 20 years
- In the course of Post Doctoral Fellowship training - 2004
- Yes, in former and more recent years, mainly courses on statistics and to a minor extent on research methodology.
- Workshop in methodology that belonged different duration from 1 day to one week
- PhD in public health sciences (quantitative and qualitative research methods) - 1998-2003 New England Epidemiology Institute (Meta-analysis), Tufts University, Boston USA, June 1998 University of Toronto: SPSS and SAS training (1998,1999)
- Masters in Public Health - Major in Epidemiology - To be completed in 2017
- 1989-1991 - as a Junior Demonstrator in the Department of Pharmacology 1992-1996 - as a Senior Demonstrator in the Department of Pharmacology 1996-1997 - as an Assistant Professor of Pharmacology
- PhD in public health (2000-2005) Other short courses since then in meta-analysis, trial methodology.
- Informal training to carry out undergraduate research papers (1960-1962) to MSc (1965-1966) and PhD (1968-1962) thesis.
- Research fellow training in epidemiology for 2 years and reserch clinical training for 1 year
- research methods courses taken in MN program
- Some in my fellowship (1983-4) but most on my own. Seminars at congresses on occasion as well.
- Research methodology two weeks
- Workshop
- During my PhD: three years
- WHO collaborated workshop on Research methodology and bio statistical writing three day workshop in 2001
- I am an NIH-funded active neuroscientist, 30 years of career so far.
- Biochemistry and molecular/cell biology
- Three days workshop at Aga Khan University, Karachi, Pakistan in 2010
- Workshop by WHO on research methodology
- Cairo University Development center 2006-2011
- Quantitative methods (Masters degree in Statistics: 1995-1998), (Doctoral degree in Statistics: 2010-2015)
- This is part of my pursuit of the Doctorate degree, as well as, standalone workshops and workshops during my specialty conferences. I have special interest in manuscripts and review articles describing research methods.
- During PhD is statistics. Later qualitative methodes
- In my scientific activity and as a university professor I have devoted to the research methodology and partly to research on editorial aspects, in what I have published several articles. I have attended courses in methodology and also I have taught.
- it was part of my curriculum of medical degree
- During MCPS health professional education gone through the research and medical writing module at college of physcians and surgeons Pakistan.
- I participate in reesearch projects on public health and use the siminar methods in research on editorial matters
- I acquired skills in research methods by doing my PhD research and post-doctoral studies.
- I am enrolled in a PhD program and have training in Research methodology, Epidemiology and Statistics
- I am an academically trained researcher - or is that not what you want to know here?
- Online courses on research methodologies.
- Several university courses for many years
- I studied the master of science because its curriculum was fundamentally methodological. It lasted 2 years, from 1998 to 2000.
- during my doctor of pharmacy training am participating in research project at the university
- As part of my graduate training at the University of Michigan from 1966 through 1972.
- I am a psychologist and we have at least 4 courses during our university that are connected with research methodology. First, Introduction to research methodology, then we have Practicums in methodology I and II (we are presented the experiments and we have to write down small scientific reports with IMRAD stucture) and Our own practicum (we do a small research and report), and then we have a one year course how to write our thesis.Finaly, we write the thesis that has to be an investigation.
- PhD in experimental and analysis focused research - 5 years. Postdoc in experimental and analysis focused research - 3 years
- MSc in epidemiology, several years research in epidemiological unit, training and teaching evidence based medicine/clinical epidemiology, setting up centre to produce clinical guidelines and teaching and overseeing critical appraisal of literature
- Electrophoresis 30+ years Molecular methodologies 15 years Westerns blots 10 years
- Currently studying for a Masters in Medical Science, which includes modules on research methods (quantitative (systematic reviews, rcts, cohort studies etc) and qualitative research.
- Masters degree in Clinical Epidemiology
- Psychologists internship, phd, fellowship awards. Primarily self-taught with formal recognition.
- As noted above. I have attended the various Cochrane Review author training workshops and also participated in courses during my MSc relating to research methods.
- Multiple courses. Ongoing academic activity with PhD supervision etc and run multiple research projects working with teams
- Research methods during PhD including critical appraisal, research design, evidence-based practice, etc.
- Good clinical practice training - regularly updated Training courses in generic medical statistics (two days) Training courses in generic research ethics (one day) Participation in training, workshops, special interest groups around research methodology (eg MRC PROGRESS group for prognosis research)
- Both qualitative and quantitative research methods courses during my BSc (1987-90) - 1 course/year lasting 12 weeks each); MSc (1993-95) - 2 courses lasting 12 weeks each; PhD (2001-05) - throughout my PhD. Additionally I taught on undergraduate & postgradute research methods and statistics courses annually from 2007-2012. I have taught a PG systematic reviewing course from 2009 & currently teach an UG systematic reviewing course.
- Cochrane course and workshops and meeting over many years
- Some coverage in Master of Public Health degree (conferred 2007) including trials, non-randomised studies and health economics studies. Multiple workshops on research methodology, including systematic review methods (which I also teach), critical appraisal of multiple research designs. Frequent informal exposure in the workplace to discussions of issues of research methods. Some workplace experience guided by informal reading in conducting qualitative studies in 2007-2009. Formal workshop on software to conduct qualitative analysis. Informal reading of academic literature on research methods.
- Associated with PhD and after related to trials and critical appraisal.
- MSc in Epidemiology, obtained 2011
- >30 years of experience as an active researcher
- Masters degree in research methods (1 year) and PhD in public health involving advanced statistics and research training (3 years)
- During my undergraduate and postgraduate study (courses between 1984-1990). Also a three-day course in statistics in 1995.
- As above
- I have a Ph.D. in physical sciences (1998-2003).
- Undergraduate practical courses, 5 years; 1980-85. Ph.D. practical courses, 4 years; 1989-93.
- Training during PhD and other one to two day courses over the past 15 years relating to a wide range of research methods
- PhD studentship 1991-95 Postdoc 1995-98
- Ph.D. and postdoctoral fellow training in molecular and cellular neurobiology.
- Masters in epidemiology which included reseach methods
- Training provided by The Cochrane Collaboration since becoming an author in 2010
- Courses Books 5 years
- I am a professional (ASA accredited) statistician, and a research methodologist, so I read and critically evaluate research methods, and the extent(s) to which analysis is appropriate and results support conclusions *for a living* as a scientist and professor. I sit on national review panels for grants as well as publications.
- B. Medical Science (including a large component on research methods and design). Currently lecture at the undergraduate and postgraduate levels on research methods and design
- I don't quite know what that means, i.e., informal. Doing research and the feedback one gets in presenting research are training in the methods. I haven degrees or certificates for such training.
- PhD courses, research training through my research, participation in large research projects, chair of a board in a national research council assessing applications, Cochrane activities etc.
- Masters in Public Health. I also do systematic reviews as part of my job so have learned about research methods.
- MSc Clinical Epidemiology, 2000
- Training in research methods as part of psychology major, over 3 years Masters of Public Health, 2 years, including specific courses on research methods and a 1 year research project PhD in epidemiology
- Master in clinical effectiveness 2 years
- Formal: PhD (3 years)
- Research Masters On going access to research methods (formal and informal) sessions over 30 years
- Various methodlogical courses in Italy. Stages in UK (Oxford, Edinburgh)
- PhD - focused on epidemiology, clinical trials, research syntheses
- MPH 2 years full time PhD 6 years part-time Adhoc workshops for advance epidemiology and health economics
- Too much to put here. PhD in pharmacology; 3 week course in Advanced Course in Epidemiological Analysis at the London School of Hygiene and Tropical Medicine; 3 day course on assessing risk of bias in non-randomised studies (ACROBAT, Cochrane); 3 day course on Prognostic Research at Keele University; plus hours and hours of Cochrane workshop training.
- I received detailed training in research methodology as part of my Masters degree (2010-2011) and I have attended specific training courses for research methodology related to my post as a Statistician at the University of Liverpool from 2011 to present
- MSc Clinical Epidemiology
- Part of my university training in Sociology (1974-1980) Various epidemiology and statistics courses 1980-2015 Cochrane Colloquium workshops 2000-2015
- 1. Regular faculty training for research methodology organized by the Institution - 2 days, every year, hands on session for using SPSS 2. Cochrane protocol and review completion workshops, 3 days, every year 3. MSc in Evidence Based Healthcare - Flexi-learning, two years, UCL
- I gained experience in methods by performing my own research. no formal training
- in general during my PhD plus courses of statistics and systematic review preparation
- Undergraduate degree in Biological Sciences, 3 years Masters degree in Genetics, 3 years
- Certificate in practice based research, RCS England
- Masters in clin epidemiology
- 2005. Workshop II° level: “Systematic reviews of diagnostic accuracy” Italian Cochrane centre, Milan 2006 Workshop III° level: “Systematic reviews of diagnostic accuracy” Italian Cochrane centre, Milan 2009 Continental Europe Regional Support Unit (CESU) of the Cochrane Diagnostic Test Accuracy Working Group Attended regularly at the Cochrane Colloquium since 2005, followed all the workshops related to risk of bias assessment for RCTs and observational studies, GRADE, meta-analysis, Diagnostic accuracy, common error in SRs. Participation in MECIR audit tool within Cchrane, Personal reading of text book and scientific articles (e.g. Cochrane Handbook)
- See # 2 ABOVE I ALSO attended the one week course given by Grimes and Scholtz for OB GYN junior researchers about the scientific method
- 1-day Critical appraisal workshop - 2004
- Informal training as an undergraduate student, (mentored research project, published in peer-reviewed journals) Informal training as a registrar and ongoing research meetings in department of medicine Attended workshops at Cochrane colloquia (3 occasions)
- Curriculums
- Through academic qualifications, and as my current job wihch is research in methodology of systematic reviews. So I would say that this is a continuous activity.
- As a professor in a medical school for 25 years, I have conducted various types of research, as well as lectured on various research methods in clinical and public health research.
- I have had 4 days exposure to research methods and statistics,Good Clinical Practice(5 days with exams that you have to pass 80%) 10 years ago with renewal 5 years ago and another last year as it expires after 5 years
- My doctoral work provided training in research methods for my areas of study. We have informal training on the job related to systematic reviews, meta-analyses, qualitative research, etc. Our role as staff editors is normally to make sure our Academic editors have all they info they need to make an assessment.
- Masters degree Professor Run a lab- 15 years
- Formal studies in Methodology (1 year)
- All issues relating to medical journal publishing/editing through workshops and courses over my entire career (eg, statistics)
- I registered and attended an MSc in Research Methodology of the University of Bedfordshire, Luton, UK for 9 months before I dropped out to return to Nigeria. I left at the point of doing the field work.
- There was one course during vet school, then one course in graduate school. I took the MOOC above and these days realize just how much I do not know as I work daily on the methods reviews and on trying to have authors utilize the reporting guidelines!
- PhD in epidemiology, 2006-2010
- Through my job as a professor our work includes two main issues ; 1. Teaching 2. Research
- MPH post MD.
- PhD is in psychometrics and quantitative methods.
- Clinical trials course LSHTM 2001
- As a trainee in paediatrics received regular teaching
- Research training during residency and fellowship.
- No
- In our university, we must attend "How to write a scientific article" workshop. We also have some courses and workshops about how to design a research. On the other hand, in order to create an experimental work, we have to attend Basic Experimental Animals Course.
- along with my doctorate programmes , in pharmaceutical sciences at medical college , trivandrum , kerala, india
- Institutional presentations on various topics.
- Teach research methods course > 10 years
- formal training from NIH USA
- Research fellowship (university department-based) for one year in 1978
- PhD completed in 2008, active researcher for 20+ years
- MPH - health services research - 1975 Multiple courses over 30 years
- during my masters degree programs and then while training masters students to conduct trainings for them
- See above, MPH with aconcentartion in quantitaive methods, completed a fellowship in general internal medicine and clinical epidemiology
- Two semester-long doctoral courses in experimental design and two in statistics, one in questionnaire design; continuing education courses in specific methods (structural equation modeling, research synthesis and meta-analysis, self-report data)
- 6 years of graduate training, which included coursework in quantitative and qualitative research methods (e.g., community based participatory research, ethnographic field research methods).
- PhD courses in research methodology (1985-87) Pre-doc and Post-doc workshops and on-the-job training in experimental design, field experiments, longitudinal surveys, qualitative methods (1986-94)

**TEXT ANSWERS FOR QUESTION #20: *Do you have any FORMAL or INFORMAL training in statistics? Please describe.***

- 150 hours for statistics since 2006 when I started my MSc
- 4 months undergraduate Statistics 1 month of medical school Biostats Informally, 3 books: how to use SPSS, metanalysis, a review of biostats. Also, my medical database background provided more informal training.
- Degree-based class in Probability and Statistics
- parametric, nonparametric
- During the course of all my degrees At statistical workshops
- Bachelor degree module, 3 months, around 1990 AS level in statistics, 1 year course, 1987
- In the course of Post Doctoral Fellowship training - 2004
- About 5 such courses attended throughout my career, both early and late in a span of 40 years.
- I participated in one week period of biostatistics
- as above. PhD in public health sciences (quantitative and qualitative research methods) - 1998-2003 New England Epidemiology Institute (Meta-analysis), Tufts University, Boston USA, June 1998 University of Toronto: SPSS and SAS training (1998,1999)
- Masters in Public Health - Biostatistics (Core Subject) - To be completed in 2017
- Informal training in basic statistics as part of the postgraduate curriculum
- 1.Basic Biostatistics training- 1 week, 2015
- During phd in public health
- Informal training as in Research methods above.
- Undergraduate course. One Semester. 1960. Postgraduate course. One Term. 1966.
- Informal through university courses and online courses Never got a formal degree
- statistic course in MN program
- Formal attendence at congresses, but most informal...on my own.
- Workshop
- Graduate work.
- I have MS degree in Statistics from McGill University, Canada and PhD degree in Applied Statistics from Temple University, Philadelphia, USA
- Quantitative methods (Masters degree in Statistics: 1995-1998), (Doctoral degree in Statistics: 2010-2015)
- Statistics is part of my pursuit for the Master degree. I looked for and read some books describing statistical methods, in order to be able to interpret the studies I read and the research analysis in the work that I take share in.
- In my PhD program on analysis technics
- I did courses many years ago, but the statistical part of my scientific activity and use. In my situation with editor, only in very special circumstances I need a statistical consultant.
- online courses and personal readings.
- I acquired skills in statistics by doing my PhD research and post-doctoral studies.
- I am enrolled in a PhD program and have training in Research methodology, Epidemiology and Statistics
- Undergraduate course
- Online courses on statistics.
- Several university courses
- Basic and intermediate courses on statistics during mastery.
- courses taken during my Doctor of pharmacy proam
- As part of my graduate training at the University of Michigan from 1966 through 1970.
- I have had one year of Statistics and one of Psychometrics (advanced statistical methods in psychology: factor analysis and reliability analsyis. But I have listened to other courses in which my knowledge of statistics was needed.
- Coursework in statistics - 1 year.
- Withing MSc - basic and advanced modules in statistics, Bayes and RCT's (1yr course), EBM courses- attended and facilitated on several Oxford teaching EBM courses, attendance at methods group statistical workshops at Cochrane colloquia, Bristol systematic review methods course
- Informal Self education
- As part of the Masters programme and certified courses run by the University where I work.
- Masters degree in Clinical Epidemiology - one course in statistics
- Advanced level in mathematics, oxbridge.
- I attended statistics courses during my MSc and also as part of the Cochrane Review author training sessions as well.
- Stata Courses
- General stats methods modules as an undergraduate (SPSS) and an optional advanced stats module covering multivariate regression, ANOVA's, etc. (1990's) SAS programming courses (beginner and advanced) (1998-99) Hierarchical Linear Modelling courses (Jon Rasbash and colleagues), Glasgow University 1998 MLWin course, Institute of Child Health, London (1999) Rasch analysis (psychometrics): Beginner, intermediate and advanced, Leeds University. 1999-02)
- University generic training in medical statisics, basic and advanced courses Specialist training in areas relevant to research eg test accuracy; meta-analysis, prognosis research
- As above - BSc module; MSc module; during PhD
- 2 weeks course in Epidemiology and Statistics in 1992
- Epidemiology and statistics courses included in Master of Public Health degree (conferred 2007) Occasional workshops on statistical methods as part of my job, largely focused on systematic reviews.
- Several courses but not expert at all - beginner
- MSc in Epidemiology, obtained in 2011
- >30 years of experience as an active researcher
- Masters degree in research methods (1 year) and PhD in public health involving advanced statistics and research training (3 years) these both involved a strong statistical component. I have also attended advanced statistical training at the London School of Hygiene and Tropical Medicine (2003).
- During my undergraduate and postgraduate study (courses between 1984-1990). Also a three-day course in statistics in 1995.
- As above
- Took 4-5 statistics classes in grad school.
- Undergraduate courses, 2 years; 1981-82. Ph.D. practical courses, 1 year; 1992.
- During undergraduate studies
- Ph.D. and postdoctoral training.
- Masters in epidemiology which included 3 statistics modules
- My Masters program
- Training provided by The Cochrane Collaboration since 2010. The Cochrane Handbook
- PhD in Cognitive sciences (1991-1997) included 1 year of stats coursework and emphasized experimental design. MPH (1999-2002) awarded in the emphasis area of biostatistics and biometry; spent 1997-2002 as a full time staff statistician for a (US) national research consortium; PhD in measurement, statistics, and evaluation (2006-2009) emphasized advanced statistical methods; doctoral level certificate in gerontology was achieved with 24 semester credit hours of applications of advanced statistical methods coursework to issues in the assessment of aging and brain aging, leading to multiple peer reviewed publications.
- See above.
- As above: statistical education came as part of ongoing research though I would describe myself as largely self taught in statistics.
- Yes, through my medical education, my phd and later, but quite minimal, I feel.
- Biostatistics module of Masters in Public Health. Also attended Cochrane training at UK Cochrane Centre and at colloquiums. Also attended regression training at the university where we are based. Also my manager is a professor of medical statistics so I have learned some things this way.
- Courses during PhD track
- First year university statistics courses, equivalent of 1/4 of study program Statistics training as part of psychology major, over 3 years Two courses as part of MPH degree (1/4 of course work) Subsequently a number of short courses on specific topics
- Master in clinical effectiveness 2 years
- Undergraduate statistics subjects
- Research Masters (quantitative and qualitative methods) Annual attendance at Cochrane Colloquium workshops
- PhD
- Part of MPH course
- See above.
- I have a BSc in Mathematics and French (2006-2010), an MSc in Medical Statistics (2010-2011) and I am near completion of a part time PhD in Biostatistics at the University of Liverpool (2012-present). I have worked as a Statistician at the University of Liverpool from 2011 to present and have attended may training courses related to my job and PhD over this time
- MSc Clinical Epidemiology - Course Classical and Modern Methods in Data-analysis
- Same as above
- 1. Regular faculty training for research methodology organized by the Institution - 2 days, every year, hands on session for using SPSS 2. Cochrane protocol and review completion workshops, 3 days, every year 3. MSc in Evidence Based Healthcare - Flexi-learning, two years, UCL
- Experience was gained through my own research, no formal training
- see previous one
- Undergraduate statistics courses, 1 year
- School A level, University, etc.
- As above
- 1992 International Agency for Research on Cancer. European Educational Programme in Epidemiology. 1993 McMaster University. Department of Clinical Epidemiology e Biostatistics. "Workshop on how to teach the Critical Appraisal of Clinical Evidence". Hamilton, Canada, 1993 Biometric Society. "Seminars on regression models in Epidemiology. Analisis of correlated data". Florence University . 1995 Azienda Agraria Sperimentale “Vittorio Tadini”. Statistic methodology, Advanced course 1997 Italian Association of Epidemiology . Master in Epidemiology. Attended regularly at the Cochrane Colloquium since 2005, followed all the workshops related to meta-analysis, Diagnostic accuracy, common error in SRs. Personal reading of text book and scientific articles (e.g. Cochrane Handbook, other textbook on statistics and meta-analysis)
- Have attended workshops, but recognize huge limitations
- Biostatistics curriculum and workshop
- I am a qualified statistician at DPhil level, and I am currently working as a senior statistician, so training and experience are continuous
- PhD, MSc, BSc
- In my MPH program, as well as attending various related workshops over the years. More informal training by discussions with statisticians when doing my own research.
- 4 days exposure to statistics at basic,moderate level and advanced each 10 years ago
- Courses at University
- Yes, university studies in Statistics (1 year)
- See question 19
- As above, biostatistics courses at university: during MD program and during employment years. In addition, biostatistics seminars and workshops during employment years.
- The workshops mentioned above have topics on statistics on the programme but I would like a formal training if possible.
- Please see above. I have given much thought to completing a MSCE as colleagues have but don't have access to funding at this time.
- PhD in epidemiology, 2006-2010
- MPH post MD
- PhD in psychometrics and applied statistics.
- Stats courses mid 1990s and mid 2000s
- As in 19 also much personal reading and listening to statistical editor colleagues
- Statistics course as medical student and fellow
- Stattistics course at medical school (50 hours)
- I have taken Biostatistics and Informatics classes for 2 years at the Medical School. During my residency, I've attended some Biostatistics workshops.
- along with my masters and doctorate programmes , in pharmaceutical sciences at medical college , trivandrum , kerala, india
- Institutional presentations on various topics.
- Certificate course taken > 20 years ago
- informal from my mentor at my research fellowship
- Informal training was part of my 1978 fellowship year
- Australian Consortium of Social and Political Research Courses in Basic statistics, factor analysis, power analysis
- MPH - health services research - 1975 Research Assist Dept Biostatistics - 5 years - 1971-76 Multiple courses over 30 years Teach EBM, Clinical Epidemiology, research methods Direct 2-year post-doctoral research fellowship 2001-2015
- during my masters degree programs and then while training masters students to conduct trainings for them
- see above
- Two semester long courses at the doctoral level in general statistics and one in regression and multiple regression, workshops/continuing education in various forms of data analysis software for data analysis, power analysis
- 6 years of graduate training, which included coursework in statistics (e.g., multivariate analysis, structural equation modeling, event history analysis, multilevel modeling).
- PhD courses in statistics (1985-87) Pre-doc workshops and mentoring on analysis of survey data, secondary analysis of administrative data and survey data, multivariate analysis, event history analysis (1986-1992) Post-doc workshops on HLM, meta-analysis
