# Supplementary material for: An international survey and modified Delphi process revealed editors’ perceptions, training needs, and ratings of competency-related statements for the development of core competencies for scientific editors of biomedical journals
Source: F1000Res. 2017 Sep 4;6:1634. [Version 1] doi: 10.12688/f1000research.12400.1 (PMC5605946; doi:10.12688/f1000research.12400.1)
Supplement: Supplementary file 5 [file f1000research-6-13429-s0004.tgz › 85cac16c-04b0-47ac-b917-b11adc1d6968.docx]

**APPENDIX D - Delphi text responses for all 3 rounds**

**Delphi Round 1 – Text Responses**

For Round 1 of the Delphi, participants were asked to make comments at the end of a section about any specific items in that section. The following are all of the participants’ comments from Round 1 of the Delphi, organized by section. At the end of this document are the participants’ responses to the question of whether there were any missing potential competencies.

**Section 1: Dealing with Authors**

- Requesting full disclosure from authors should be a matter of process rather than editorial competency. Editors may be required to have competency in assessing declarations of conflict? Some of the above (negotiating publication delays, supporting language issues, communication of decisions, clarifying peer review process) could be dealt by other journal staff or information resources, not necessarily scientific editors. 'Ensure that requests from authors that individuals not peer review their paper' - not a competency. Determining whether these requests are well-reasoned and acting accordingly might be a competency.
- If I have downgraded some of these attributes and behaviours it is not necessarily because I rate them as unimportant, just that I do not view them as core to the scientific editor role
- Disputes over authorship are matter for the group of researchers and their institutions; the editor should clarify the criteria to be considered as author
- what is appropriate documentation?
- The two issues I have rated below deserve comments. I was editor of two journals, including both 20 years. My current publication is published in two languages, and is the editorial itself that performs the translation from another language that authors must accept. That is, not to be helped in the language, is to be asked to write in their language and the journal is responsible as a free service to the other version. The second point is that I do not think that is a function of the journals to professionals without experience in research, do research
- There is only so much 'support' that can be given to authors who transgress and to people who first language is not that of the journal or editor. Goodwill and fairness should be the order of the day. As ever, the editor may delegate. Not sure what this involves: "Work with publishers to defend author rights and pursue offenders" Clarification also required for: "Seek to help authors understand magnitude of effect"
- For some of these, it will depend on the specific journal.
- Some questions are hard to answer because I would give different answers to the two parts, e.g. Interact with authors to confirm undisputed changes in authorship and act on any institutional findings concerning authorship disputes - I would say yes to first part and no to the second. Similary with "Act on concerns about plagiarism, data fabrication, or an authorship issue and follow up with authors and then institutions"
- "ability to work with authors from developing countries" is discriminating against them. Suggest to re-formulate the question
- Our journal has other experts to call on to deal with some of these.
- re "Review study protocols and methods and encourage authors to make them publicly available", I don't think (based on my own work in implementing statistical review etc) every scientific editor can reasonably be able to evalute study protocols and methods but if the journal doesn't have statistical or methods editor(s) on staff they need to have access to people who can do this. Journals do need to clearly articulate their expectations of authors in terms of access to raw data (including timeframe following publication). re Engage in mentorship and education of authors to help them produce work to best effect. Again, this has been something I do and has been rewarding (and there are authors I have worked with from residency through promotion in their faculty appointments). Some editor colleagues previously thought I "helped too much" however turnaround time decreased and authors continued to submit manuscripts. Helping does not mean being "easy" on authors, it can be the way in which you communicate to encourage authors to achieve a high standard (and to comply with stat reviews etc). Seek to help authors understand magnitude of effect. I'm sorry is this understanding effect size?

**SECTION 2: Dealing with Peer Reviewers**

- 'Develop, facilitate and monitor' peer review - could be done by other staff at an admin level day to day, although policy-setting should be within the competency of scientific editors. Train peer reviewers - this kind of training should be done by competent editors, but not all competent editors have to be good trainers. 'Ensure all comments are seen by all peer reviewers', 'Ensure decision is made when reviewers fail to submit' & 'Ensure reviewers keep manuscripts confidential - these are policy & process issues rather than competencies. Demonstrate publication and reviewing skills - I think this duplicates one on the previous page about being a competent researcher? 'Demonstrate the ability to recruit peer reviewers' - this could be done by others in the editorial team.
- I don't know how you can refuse sloppy reports. You can certainly make a note of these and not invite the reviewer again. I haven't ever asked someone to re-do their review; I'd be interested to hear if other editors do this.
- The experience of the reviewers is acquired with practice. Then the editor is forming his pool of reviewers with care and training
- some of those statements are poorly or unclearly stated.
- Extremely important - To inform the peer reviewer that their comments will be published alongside the review. (To inform the reviewer whether there is open or closed peer review.)
- Ensure a very high standard of the referees don't accept sloppy reports from anyone. This is badly written
- Some of these tasks can be part of the editorial 'team' or delegated - for instance, ensuring confidentially. Never sure whether a 'sloppy' report from a peer reviewer is worth the effort of chasing up - just don't use them again.
- Some items are policies, not skills
- "Ensure reviewers who consistently produce discourteous, poor quality or late reviews are removed from the journal’s pool of peer reviewers": essential for first two, not for the last. If we eliminated all late reviewers, we might not be left with many. "Ensure that reviewers keep manuscripts, associated material, and the information they contain strictly confidential." There is no way that an editor can ensure this. They could act if they found that such a policy had been breached but they cannot police reviewers.
- Some of these depend on the journal, structure and help from other editors and editorial board, and all editors get better at many of these with experience.
- Ensure thorough statistical review. I believe this, but many of my fellow editors don't! Ensure a very high standard of the referees don't accept sloppy reports from anyone. Current publishing platforms eg Scholar One don't always make it easy to "rate" reviewers so unless time is spent in getting to know reviewers such 'sloppy' or just unhelpful (eg "very nice paper, I enjoyed reading it"....) often remain in the system.

**SECTION 3: Journal Publishing**

- 'Demonstrate knowledge of specifications of the journal' - not sure exactly what this means? But I guess you should know as much about your journal as possible. 'Ensure that research is correct' - not sure if anyone can 100% ensure this, although many of the other competencies are designed to look for this (e.g. knowledge of methods, ensuring statistical review, knowledge of fraud, etc.). Does this somewhat overlap with previous point about checking for accuracy? 'Act as a gatekeeper and guarantor' - this seems a bit grandiose. Checking quality and scope seem like umbrella statements that should be covered by more specific competencies.
- Possessing this knowledge and skills is essential for the proper performance of a scientific editor in connection with the publication of its biomedical journal
- In my opinion, most of the issues are essential to the work of an editor in the usual scientific journals. It is possible that some of them are not needed in very large publications a reasonable specialization of the editorial team. I have not considered as essential reprints and layout processes because there are very technical and although in my case I had been involved in that designs, has been only as suggestions and not as executive form. In addition, in most of journals I think the audio aspects are not import ants.
- Although the editor is the final judge, they cannot "Ensure that selected/published research is correct", they can just take reasonable actions/steps to reduce the risk that the article is incorrect.
- Too many items to make discrete judgments.
- "Ensure that selected/published research is correct": what do you mean by correct? The peer reviewers should ensure that the content is correct as far as is known to science at this point. The editor cannot judge the content or do you just mean ensure via peer review?
- First question - marketing/advertising and ethical - I only put high rating there because of ethical aspect, I think this should be separate to marketing/advertising in general (e.g. strategies) where I would rate lower
- You ask about "correct research". I don't know what that is supposed to mean.

**SECTION 4: Journal Promotion**

- *'Stay on top of updates' duplicates 'knowledge of important developments' 'Decisions based on validity and importance' are two different things that should be separated. Importance to readers not always a criteria for publication for some journals & may contribute to publication bias, although an important part of the selection process for other journals. Understanding of constituency - not sure what this means. Readership? Responsibility to the scientific community not really a competency, but could be demonstrated through other ethical and methodological competencies. Many of the items above relate to public relations - it's essential that some editors do this, but not something every editor must be competent to do.*
- *Physicians are not necessarily your target audience - even in what is called "biomedicine" (nurses, therapists depending on topic...)*
- *Again some of this work can be shared with others*
- *These actions drive the promotion of scientific journals. I think the researchers want to publish their results in a journal that is promoted in this way to readers, peers and decision makers*
- *Engaging with social media and writing press releases would presumably be done by others.*
- *One of the important options for journal promotion is its presentation at professional conferences. So science editors (most of whom should be also actively engaged in research) would be expected to attend such conferences with/without research presentations and promote their journals.*
- *The editor can delegate - some may be all-rounders but some may not be that good, say, at comminucating with the public*
- *Some of these are critically important, but not, in my opinion, the editor's job*
- *Motivate physicians - what about every other health professional in existence?*
- *these are important especially with the rapidly changing landscape of publishing but some journal editors are more or less engaged in a lot of these areas I think*

**SECTION 5: Editing**

- 'Demonstrate broad and detailed knowledge of the skills needed to refine a piece of scientific work and shepherd it through to publication' - not a competency. This process is trying to identify what the skills are that are need to do this. Unclear what's meant by online editing - editing website content? Using online editorial systems? Editing for online publication? 'Demonstrate the ability to select material for its merit, interest to readers, and originality alone' - this is a bit vague and seems to roll several concepts together without enough clarity. Selecting for interest to readers not appropriate in some journals (e.g. journal of negative results trying to avoid publication bias). Selecting for originality might prevent publication of important replications. Triage and preliminary opinions seem related, depending on journal process - are both trying to get at the initial appraisal of whether a manuscript has enough face merit to proceed to peer review? "Make fast, good decisions' seems like two competencies - fast and good? "Make difficult decisions" not clear enough. Do they mean complex judgements about methods, controversial rejections of prominent researchers, navigating conflicting peer review, managing hostile responses from authors, etc.? Excellent judgement not clear enough - judgement about what? Select, curate and comment on - more of a job description than a competency? Possess a degree - no. Be trained as an editor - yes, but this is not a competency - it's a way to achieve the required competencies. Types of manuscripts - is this duplicated earlier? 'Multiple research epistemologies' - don't know what this means. Ways of knowing through research, i.e. types of evidence? Supplemental materials - seems like this is a ethical publishing issue, unless there are content/ethical issues around this that I'm not across.
- Again I have downrated some of these factors because whilst somebody has to do it it doesn't always have to be the Scientific Editor
- A person could learn how to use the editing software, but they already need a good understanding of what constitutes good research.
- Communication with the corresponding author during the publishing process is a valuable resource for training. The online edition is a competition that quickly acquires
- Extremely important - knowledge of ethical standards in medicine and research, such as the Declaration of Helsinki and others!
- Journal editing also requires knowledge and skills in updating instructions for authors and full awarness of updated recommendations of Council of Science Editors, ICMJE and other leading editorial associations.
- In this group of items, most are technical aspects that in my opinion are acquired when they act as editor and so I have lowered the level, not so much because they are not needed, but because develop during the activity. However, experience, the habit as author and reviewer, knowledge of research methodology, clinically relevant or not. I think are very important. the adquisition of editor competences depends of techical learnings and experience as author, reviewer and editor.
- As in all walks of life, there will be a range of acceptable abilities. Some editors may not be that 'quick' but, perhaps in some more reflective types, their decisions may not be so arbitrary or just plain wrong!
- Not all items are clear
- "Ensure papers selected are clinically relevant ": it's a biomedical journal, not a clinical one.
- Ensure papers selected are clinically relevant - I think importance of this would depend on the scope of the journal...? Possess a degree in medical editing or be trained as a journal editor- I think this is the purpose of this study? Few people will have advanced degrees and training in medicine and also have degrees in medical editing probably, and most of us acquire experience as journal editors 'on the job'...? Ensure papers selected have a clear story-line- importance of this might depend on the specific journal style and manuscript category? Demonstrate experience and/or training in medical journal writing - my apologies, is this experience of having published in medical journals? Or experience in AMA style? I don't know what 'medical journal writing' is... Be working towards a deeper understanding of multiple research epistemologies. Nice question which I think most of us will have to google!

**SECTION 6: Ethics and Integrity**

- 'Recommend publication of papers...' seems like a consequence of understanding the other issues, rather than a separate competency? Image manipulation a critical issue but not relevant to all areas of scientific publishing.
- One has not always had the opportunity to DEMONSTRATE something (e.g., competence) if the issue has never arisen.
- Actually each of these is critical to the process, but I feel that some of the expertise can be found with the publisher and there can be shared responsibility
- Again, all of these are important but I would argue that some are not the main responsibility of the editor-in-chief but rather other actors in the publication process
- In this regard, training to all actors in the process of publishing scientific journals also plays a central role
- Science editors should also be aware of free and commercial plagiarism detection software (e.g., iThenticate).
- It is not the editors job to ensure that all institutional review decisions are send checked- if they are presented as OK by the IRB review the editor needs not do this.
- or me they are key issues, and has published articles in this conviction. You can not use scientific communication as not to have merit and publishers are responsible of that.
- Again, you can only try your best. So, you may not be able to detect all fraud / image manipulation. Awareness, proactive monitoring, and addressing issues if / as identified are about as much as you can do. And again, you can be a gatekeeper but you can't be expected to "ensure" good research conduct or "safeguard" rights of study participants - probably going well beyond remit. In truth, I'm not quite aware of any special issues relating to " dual-use research "
- Not sure what some of these items mean
- i dont understand what you mean by dual use research

**SECTION 7: Qualities and Characteristics of Editors**

Delegate - may be the responsibility of others in the team. Learned societies - no. Editing-related organisations - if there is one available/appropriate to your geographic location. Current research portfolio or employment - not essential although competency in research through past experience might be. Past experience on editorial board - not a competency, but a way to attain the required competencies. Everyone has to have their first editorial position! Competence as a practitioner - not essential if you mean clinical practice, but more so if you include research practice. Rigid criteria - for what? Depends on whether the criteria are appropriate. Practicality - what does this mean? Personal interest in journalology - no. Professional interest & knowledge of field, perhaps. Doesn't have to be their hobby. Patience - good communication more important than patience behind the scenes. Managerial skills may sit with others in the editorial team.

1/28/2016 1:47 AM View respondent's answers Categorize as... œ

First question conflates the field (important) and the people (not important).

1/27/2016 5:01 AM View respondent's answers Categorize as... œ

Oh my. All must have. It's a complex job

1/26/2016 10:12 PM View respondent's answers Categorize as... œ

I didn't understand the meaning of some of the items, so scored them "Not at all important". Perhaps a category that allows one not to rate an item would have been useful.

1/25/2016 5:45 PM View respondent's answers Categorize as... œ

The question about professional practice is very clinician centric and completely irrelevant for some journal editors.

1/25/2016 3:12 PM View respondent's answers Categorize as... œ

Boldness is a double-edged sword and maintain strict criteria can hinder the arrival of new models or practices. The recognition from others in our field of work may be influenced by fickle issues

1/21/2016 9:58 AM View respondent's answers Categorize as... œ

An editor can be a very full role and so it can be unrealistic to expect them to carry on in their 'own' field of study. Also, while you might have a special interest it is important not to let this badly influence your decisions

1/9/2016 10:34 AM View respondent's answers Categorize as... œ

"Demonstrate the ability to perpetuate or challenge master narratives": I didn't understand this one.

1/7/2016 12:53 PM View respondent's answers Categorize as... œ

I am not confident that I understand the questions I have left open correctly.

1/6/2016 3:33 PM View respondent's answers Categorize as... œ

- "Demonstrate the ability to perpetuate or challenge master narratives" I may be misunderstanding but wonder if this is contradictory (perpetuate vs challenge)? - As far as some of these core competencies, perhaps for some it may be that some of the editors at an individual journal can contribute expertise while others might have other strengths (eg knowledge of processes related to editorial board), but others (most on this list) in my opinion certainly are necessary for all such as integrity and accountability, relevant experience etc

**SUGGESTIONS FOR MISSING COMPETENCIES**

- Able to assess appropriateness of research methods in relation to the stated research question. Able to assess validity of conclusions in relation to the research findings. Understanding of issues relating to reproducibility of research. Understanding of issues relating to selective outcome reporting (although granted this is an aspect of publication bias, which you did list)
- Nothing could possibly have been missed from this exhaustive (exhausting) list!
- Conflicts of interest was touched on. But it is important for editors to be on the look out for conflicts that have not reported by authors, and the need to deal with these.
- Having training or expertise in how to correct the published record
- An understanding of different peer review models to improve efficiency of reviewing processes, such as cascading
- understand what is impact factor Understand how citations can help with impact factor
- I think all the desirable competencies that a scientific editor should possess were addressed in this study
- Good command of Scientific English
- engage with copy editors
- After having gone through all these material, one realize the insufficiency of most persons (including oneself) in filling such a position.
- Ability and willingness to arrange regular editorial meetings to discuss matters arising.
- not much
- It would be helpful to actually define what a scientific editor is at the start. Extra one below: Ability to check that you are making consistent and transparent decisions.
- Involve patients, research subjects and communities in journal policy
- What level of editor were you referring to? I interpreted it as meaning EIC. Your note should have come at the beginning.
- Be familiar with more than one language, that means be really aware of cultural diffences and have a wider vision
- The ability to handover his/her tasks and duties to the next scientific editor.
- Although most of the traits/competencies are very important, however, it may not be possible to find a person possessing all the traits.
- Language competency particularly from a country where English is not the native-tongue
- all competencies for editor where asked

Delphi – Round 2 Text Responses

1. Review study protocols and methods and encourage authors to make them publicly available

• I think protocols are essential and a basis for editorial work

• Increase transparency, reduce the risk of outcome reporting bias

• The role of the editor is eminently educational to encourage authors to comply with the law of transparency of information and best investigation and editorial practices

• Reviewing protocols is a different activity from establishing journal policies to make them available

• Poor methodology may discredit the results if published in my journal, or be a cause for rejection

• Public availability of results is of utmost importance

• I'd expect higher score.

• This is a fundamental pillar of transparency and replication. The pre-definition of methods is a key way of demonstrating how for the research as undertaken adheres to the intended objectives of a study. Editors should be more engaged with tracking deviations from what was intended.

• More of a task for institutions and regulatory bodies than editors

• Journal editors need to take a lead on transparency and improving conduct/reporting. I would give this a higher priority.

• This does not apply to all types of scientific journals.

• My original score was close to the average rating; I think this needs to go up considering the importance of the issue

3. Seek to help authors understand magnitude of effect

•This is important, but I do not see at as being the responsibility of journal editors. Rather, this should be part of the training that researchers obtain during their studies.

•If this is effect size or interpretation of results

•It is in the interest of the journal to help the author to demonstrate in a clear way the strength of the published research

•Author education is not a central part of an editor's remit. We can point out that a result claimed as 'significant' has a small clinical effect but I do not think we should spend a lot of effort in helping authors to understand this.

•This criterion seems too focussed on trial reports and systematic reviews. Many scientific editors will work on multiple article types (news pieces, commentaries, reviews of mechanisms of disease etc.), that do not focus on magnitude of effect. For those articles that do include magnitude of effect, the role of the editor should be to confirm that the conclusions are consistent with/can be justified by the magnitude of effect. I don't believe the primary role of editors should be training or education.

- I would say it is the Editor's responsibility to understand magnitude of effect and identify poor understanding or communication in manuscripts, but not to train authors in these competencies.

•If the authors do not understand the magnitude of an effect they will not be able to communicate it effectively.

• Authors should be responsible for their own learning

• I'd stay with my score; I am not sure that this is the most important of editor's roles - this should be gained during medical education

•I think that this is very relevant

4. Assist potential authors in developing a spirit of inquiry

•As I interpreted it, I feel it’s essential to encourage particularly young authors to continue in their research endeavors and to continue to ask questions, be curious. If its an author whose paper we've accepted, this will encourage continued output from a previously successful author.

•This is important, but I do not see at as being the responsibility of journal editors. Rather, this should be part of the training that researchers obtain during their studies.

•Helps lift the articles to be more interesting / exciting

•This has nothing to do with the editor

•I think the authors should already have a developed spirit of enquiry when they accept a commission, or submit a manuscript. If the manuscript poses questions that do not seem to be acknowledged or addressed by the authors, these should be raised by the editor.

•While a culture of enquiry is health, assisting authors in developing this spirit is not an editorial competency.

•A role for academic institutions and other employers, not editors

•Not a task of an editor - least of all for "potential authors" - how many are there not?

•I think that this is very relevant

5. Develop wide acquaintance with potential authors

•It is not necessary to develop wide acquaintance, it may cause bias.

•A journal editor must know who the best people/authors in a field are and proactively encourage them to publish in their journal

•It is important for new journals or those that are not doing particularly well

•To promote the arrival of research results from new authors

•For our journal we need authors so this is important

•It is a good marketing strategy

•In my opinion, the average score is low. Most papers that have discussed the roles of the editors talk about the need for authorship networks are generated in relation to scientific journals. Smith, the former editor of BMJ, put it as a function of the editors. I not think that is a specific role of the editor in chief but editorial teams.

•A network of potential authors, who are also peer reviewers, is important for the development of the journal

•This seems to be outside the scope of editors

•I see this also in the context of promoting a journal

•I can go with the average, but this issue is not as straightforward - how do you develop wide acquaintance with authors if you are a global journal; if this means that your authors get to know you from you publications and activity in the field, yes, it is important).

8. Engage in mentorship and education of authors to help them produce work to best effect

•I rate this higher--completely aligned with my reasoning in query 4. If we have a successful author or a nearly successful author, for the good of the specialty and your patients, and our journals, we should help them gain additional insights by making sure feedback is high quality.

•Still think that it helps to be constructive

•To improve the quality of the papers submitted to the journal

•This is important at the university level

•The experience of the editor should help the prospective authors to conduct and publish good trials

•This is a 'nice to have' competency for editors who are interested in training and mentorship. It is not a core competency.

•I see this as a productive role for editors

•Although I think editorial comments to authors can be educational, I do not believe it is the place of the editor to act as mentor, unless they have another relationship in addition to author/editor.

•A role for educational and research institutions, not editors

•In an ideal world this is important and would get a higher grading; however in reality time pressures and other commitments may not allow editors to work with author teams to substantially raise the quality of the submission

•I think it is duty of editors to be involved in teaching authors

•I think that this is very relevant

11. Interact with authors to confirm undisputed changes in authorship and act on any institutional findings concerning authorship disputes

•Once all of the facts are in hand, who else should act on authorship disputes but the editor?

•Do not believe this is our role

•The average score is low. Conflicts of authority are the result of misconducts and everything that is related to behavior are the basis of the journals are an element of transmission of knowledge. We carry a decade in the EASE devoting a session at the biannual meeting on misconducts, so I understand that is a core competence

•Essential task for preserving and enhancing the journal's integrity

•I will decrease my score. This is not so important

12. Clarify the peer-review processes to authors

•Transparency is critical - who else should provide the details of a journal's peer review process except the editor?

•The peer review process is not a black box

•They should read instructions to authors for such information

•Editors should save their time by clarification of the peer-review process. It allows them to put more time on the review process itself instead of answering to authors' questions about the process.

I will decrease my score. This is not so important

13. Negotiate manuscript publication delays with authors

•In today's publishing environment, this should be irrelevant

•It is useful for planning the publication, and when a paper can be improved for subsequent publication

•This is a publication process matter, not a scientific editorial competency (although editors may take this role in some journals)

•Manuscript publication must be on time if that manuscript galleyproof is not approved we will put it up in the next upcoming issue

•There are journals that publish articles as they are accepted; negotiating delays is more relevant when problems occur during the review process and not when a manuscript is accepted and waits for publication

•I will decrease my score. This is not so important

15. Ensure authors are informed about journal and article information and/or funding

•An editor must be aware of this information and be able/willing to communicate it

•I don’t quite understand this item.

•If "funding" referring to publication fees then it is essential that authors are aware of this. Overall I feel it is important that authors are given as much information as possible regarding the preferred format of submitted articles

•This is too poorly worded to rate clearly. What is 'article information'? Authors should be informed about the journal and its funding, but this may not be the responsibility of scientific editors.

•Editors' role is to ensure their journal has clear instructions but it is up to authors to ensure they inform themselves of that information

•This is primarily an author's responsibility.

18. Provide active encouragement for revisions of manuscripts

•If an author is being asked to revise a paper, that means we are very interested in the paper. We try to do our rejections prior to revision and spend much more time at that point in the review process. if we've sent for revision, that means we will likely want the paper so I want to encourage that the authors revise.

•Not a primary responsibility of the editor

•The interaction of publishers with authors and reviewers is vital for the smooth running of the journal

•I do think that it is important that authors are encouraged to consider the suggested revisions of peer reviewers rather than to submit their article elsewhere

•I'm not sure how providing encouragement is a competency, let alone a core one

•This is really a core issue for the authors, not the editor

•I would support engagement with the revision process, but not sure if 'active encouragement' is the right way to describe the critical competencies.

•Yes. If one says "revise" then it is a certain commitment towards the author

•On a case-to-case basis

•It is very difficult to find good reviewers and sometimes encouragement is useful

•I will decrease my score. This is not so important

21. Work with publishers to defend author rights and pursue offenders

•Unsure of what this means

•I don't think as a core competency

•For best practices in research and publication

•A good skill for those interested in this but not a core competency

•I think that this very important

24. Support authors in dealing with breaches of copyright and plagiarism issues

•I rated this as very important. This issues can be bewildering to authors. iF the paper is one we are interested in, we should provide resources and advice (even as simple as referring to COPE) to help them. Particularly with self plagiarism, my experience is that most people are unaware of this issue and its not nefarious.

•This is important, but I do not see at as being the responsibility of journal editors. I suggest that copyright infringements are the responsibility of the Publisher. Plagiarism is the responsibility of the researchers' institution.

•I still think this is a shared role and thus shouldn't be seen as a key core competency

•Publishers should not stay out of these violations, as they would be accomplices

•Supporting authors is not the competency. Knowledge of copyright and recognising plagiarism are.

•I think it is very important that editors support authors in such cases

•My opinion is that in this case editors often act as educations, so this should be an important competence for them

26. Demonstrate the ability to work with authors from developing countries

•Although our journal is a US based journal, it is read internationally and about 15% of our content is from outside the US. In the not-too-distant futuere, a lot of excellent research will be coming from the "Rest of the World" and we better be in a position to be able to work w/ these authors.

•Developing countries have good authors and honest researchers. There is a need to encourage research from developing countries

•Very relevant to encourage participation from developing countries

•As with authors from developed countries, in order to ensure the dissemination of research results

•I am an editor from a developing country. Current and prospective authors are from the country or the region and I must understand and deal with their limitations

•Science is global

•Editors should demonstrate the ability to work with authors from ALL countries.

•The way this is phrased is unclear and not ideal - perhaps should refer to dealing with authors from diverse economic, cultural and language settings, or something?

•This can be rewarding and perceived as most helpful by these authors.

•Increasingly a reality

•I think it is extremely important that non-native English authors from developing countries are treated fairly.

•I find this question rather inappropriate, implying that authors from developing countries are less worthy of publication in good journals - they are the same as those from developed countries and need the same approach

•This is very important

27. Assist non-native speakers in dealing with language issues

•I gave a smaller rating here because while I do feel it is within the editors’ remit to identify language issues that affect the evaluation/readability of the scientific content, I do not think it is necessarily the editor’s role to copyedit the manuscript, that can be handled by other parties –e.g. copyeditors.

•This is important, but I do not see at as being the responsibility of journal CONTENT editors

•Language should not be a barrier

•Very relevant to hep and encourage publication of non-native english authors

•In order to ensure the dissemination of research results

•Identify issues but not necessarily assist in resolving them. Authors need to get author help.

•agree if we direct them for help

•Authors should have sufficient language skills to submit a paper to a scientific journal. Minor wording and grammatical errors can be corrected, but if the meaning of the manuscript is not clear to the editor because of language issues, it should not be the role of the editor to remedy these issues.

•Yes, should be done with sympathy and in an educational spirit.

•And that non-native English authors are given a chance to present their results in a way that does not prejudice a reader.

•To an extent, but editors cant provide a proof reading service

•This is very important (science needs to be promote)

29. Knowledge of different types of peer review

•This goes hand-in-hand with item 38 and editors should know about the latest research on the effectiveness of different peer review models

•It should be known by the editor

•I believe it is essential that an editor is aware of different types of peer review (methodological, statistical, subject expert etc.) and have an awareness of which types of peer review are required for different types of articles

It is changing world and we might as well be knowledgeable of alternate models

30. Encourage and demonstrate awareness of new findings on peer review and publishing and how these influence their journal's processes

•Publishers must be current in their field of work and how they influence the progress in their journals

•Scientific publishing must adopt an evidence based approach to its practices.

How one would demonstrate awareness of new findings? One may be aware of the new findings but this may not be relevant for the journal?

35. Evaluate and provide feedback to the reviewers on review quality

•I want to have a list of terrific reviewers and reviewing is not an innate talent. By working with reviewers and providing feedback, I help to ensure that I have excellent reviewers and I provide them feedback even if is just a quick "thanks for a great review" which makes it more likely that a reviewer will continue to review for us.

•You don't have to send the feedback but you should be able to do this

•Good reviewers are scarce, we must "cultivate"

•Good service to reviewers is to share other reviewers' comment. I'd expect score 5.

•I think this should be done more often and in a more personalized way by editors-in-chief.

•Rather than fight this uphill struggle, I'd advise editors to drop reviewers if they provide reviews of unacceptable quality.

•This is important but is infrequent in the journals i work with

•Without feedback reviewers do not know if they are doing good job or not and what are the expectations of editors

•This can be done my another team member

39. Train peer reviewers

•I do not necessarily disagree but I am not aware of many mechanisms in place to provide training for reviewers, I would be interested in having information about resources available for this.

•Critically important--same answer as #35.

•This is important, but I do not see at as being the responsibility of journal editors. Rather, this should be part of the training that researchers obtain during their studies.

•Identify potential reviewers and train them assures us a good evaluation of the papers submitted to the journal

•Ok my rating should have been higher

•Not role of editor

•Disagree that training skills are a core competency for all editors (no. 35 above, ability to evaluate and provide feedback to reviewers probably more appropriate).

•Still think this is low priority given lack of evidence for value of such training

•Or avoid peer-reviewers who are just poor...

•Training peer reviewers is important; i am not sure how the editor of a large journal can do this directly

•Evidence shows that training different forms of training or peer reviewers are not effective)

•This can be done my another team member

40. Ensure reviewer comments are shared with all peer reviewers

•This should be at least partially open if review will not be fully open. Also helps educate the reviewers

•Not really a competency

•It is part of the continuing education of the reviewers

•Ok my rating should have been higher

•This is a matter of journal policy, not a competency.

•May not be desirable

•Id' expect 5

•This is a policy issue and admin task, not a competency

•A nicety but not essential as the editor's prime duty is to readers not reviewers

•This is not what I do. I recognize it as desirable, but it would add one more round of moderating comments before transmitting them to third persons, and in the small world I'm working in, anonymity between peer reviewers would be almost completely fictional.

•I don't see this as being key

46. Ensure a very high standard of the referees don't accept sloppy reports from anyone.

•Who should do this other than the editor? And not doing it serves only to ensure low quality reviews.

•Hard to ensure, can accept sloppy report just don't use and move on

•That way we ensure the quality of the publication

•Of course, do not accept sloppy review work, but "standards" may vary across variety of reviewers. •Sometime patient or practitioner reviews are valuable,even though they may not meet "standards."

•Poorly worded. See comments re: no.44 above.

•This can be a bit tough, it is correct, but often one is stranded with a poor review and has to make th best of it due to time constraints.

•I am surprised many of my peersare prepared to accept sloppy reports. One such and I would not use that referee again.

•This is quite essential. There is nothing to be gained from bad reviews

•It's a small world - and even referees of less than very high standards may have their good points

50. Demonstrate the ability to successfully recruit peer reviewers

•Same answers to 35, 39. We have to have great reviewers.

•Finding peer reviewers is key to being a successful editor

•Who else should recruit peer reviewers?

•Because in good reviewers lies the quality of the publication

•Increasingly difficult

51. Demonstrate knowledge of marketing and advertising policies, including ethical issues

•Journals with paid advertising should know about separation of advertising and editorial but also be able to appraise content/claims in and legal issues about advertising

•Marketing is not the responsibility of a content editor. Being an ambassador for the journal, yes. Marketing, no.

•Editors should handle these issues as they are part of their field of work

•Many editors have nothing to do with marketing or advertising

•Breaches of ethics where commercial funding is involved are critical to avoid.

•I'd go with the consensus if "knowledge" is to be understood as "awareness".

•We do not allow advertising and marketing in our journals

52. Demonstrate knowledge of the article embargo process

•I would give this a lower rating, embargoes for manuscripts to be published do not necessarily need to be handled/monitored by the journal’s editor(s)

•Mainly the responsibility of the Publisher's production department. The editor needs only know that it is possible

•This does not apply to all editors or all journals

•Editors should handle these issues as they are part of their field of work

•This is very important to the success of any press release. If you have only a limited grasp on the importance of things like getting the DOIs right then you will find that there will be less coverage and you will achieve less impact of the research you publish.

•Same as 51

53. Demonstrate knowledge of indexing services

•This is more important to a publisher and is not paramount to an editor

•Without indexation there is no visibilty and hence low impact

•Editors should handle these issues as they are part of their field of work

•Why?

•An essential item for authors but not editors

•same as 51

•This is managed by Universiti Sains Malaysia Press official of the journal section

•This is an important issue for most small and scholarly journals; very often editors have misperceptions of indexing services, as well as unrealistic expectations, so knowledge about these issues is very important

54. Demonstrate knowledge of reprint processes

•I would give this a lower rating, reprint requests do not need to be handled by editors, in my experience this is handled by journal/publisher staff more involved in operations or marketing

•This is just technical stuff - someone else will know

•Editors should handle these issues as they are part of their field of work

•Not important any more with internet

•Why?

•I think in these days this is bay and large irrelevant.

•Perhaps more the job of the publisher

•This is managed by Universiti Sains Malaysia Press official of the journal section

•This is rather obsolete now in online publishing, may be relevant for big commercial journals publishing pharma research

55. Demonstrate knowledge of the specifications of the journal

•Otherwise you will wrongly advise - waste time

•Editors should handle these issues as they are part of their field of work

•How can you process an article if you don't know whether it matches the specifications of your journal?

•A matter for management not content

•This is part of the role of EIC

•This is managed by Universiti Sains Malaysia Press official of the journal section

57. Demonstrate knowledge of formatting of layout for journal issues

•I would give this a slightly lower rating, while it is relevant to have some knowledge on formatting issues, this is not in itself critical for the evaluation of a manuscript or for the development of editorial policies

•This is a job for production editors, not scientific editors

•Editor in chief may not even need to know this

•Formatting is actually more important

•It is fundamental to know about layout. Helps if you do the proofing

•The editor should know the whole publishing process

•Production these days is generally off shore

•Editors really should make efforts to advise authors on this

•same as 51

•This is managed by Universiti Sains Malaysia Press official of the journal section

59. Demonstrate knowledge of the different parts, purposes, and characteristics of different types of journals

•Editor in chief may not even need to know this

•Editors should handle these issues as they are part of their field of work

•same as 51

•This is managed by Universiti Sains Malaysia Press official of the journal section

•I still think that this is not a crucial issue for an editor

60. Demonstrate understanding of the editorial office and operations

•Editors should handle these issues as they are part of their field of work

•Essential that editor be part of the wider team to ensure high morale

•If there is a separate editorial office, they should be trusted to do their work independently

•This is managed by Universiti Sains Malaysia Press official of the journal section

63. Be aware of how design can be used to improve the readability of a document

•This is not a task for a scientific editor

•Editor in chief may not even need to know this

•Editors should handle these issues as they are part of their field of work

•Important, but not the scientific editor's remit.

•These are issues for the layout editor and the desk editor.

•I think this comment is alluding to reporting guidance such as CONSORT; this needs greater emphasis

•I don't see how this can be too important for an editor

67. Demonstrate knowledge of journal metrics and research impact

•Editor in chief may not even need to know this

•These are elements that help improve quality of periodicals

•I cannot understand the average punctuation obtained. Knowledge of the metric at impact is a core competence of the editorial teams, basic in making editorial decisions, not only for the chief editor but for the entire editorial team.

•A much overrated topic, and increasingly becoming less relevant.

•It prevents editors enter into unnecessary activities and discussions

68. Demonstrate knowledge of online publishing and products

•Knowledge of online publishing can help improve web traffic

•Publishers must be current in their field of work

69. Demonstrate knowledge of the parts, purposes, and characteristics of audio and video clips

•As long as someone does

•Publishers must be current in their field of work

•Same as 51

•Going forward video and social media content will be increasingly important; I would give this a higher priority

•This is managed by Universiti Sains Malaysia Press official of the journal section

70. Demonstrate awareness of intellectual property issues and work with publisher to handle potential breaches

•Publishers must be current in their field of work

71. Demonstrate knowledge of technical-economical aspects of medical journal production

•Can’t run a journal without finances. it has to be a good business model

•Publishers must be current in their field of work

•Not a core competency unless in a one-person journal

•More of an issue for the publishers than for the editors. And applies to medical journals only...

72. Explore and embrace innovative technologies

•Still don't think of this as core. Help should be available

•Esp technologies related to internet

•Publishers must be current in their field of work

•In fact, this is a technical problem, not a scientific one

•I recommend a careful conservatism in these issues

•This is managed by Universiti Sains Malaysia Press official of the journal section

73. Maintain close contact with the latest trends in electronic media (e.g., tablets)

•I would give this a lower rating, there is a need to stay up to date with technological advances and features to ensure the discoverability of content but I see this as less important for the editor’s role than other items, also this is something that other roles within a journal can handle beyond the editor himself/herself

•This is a job for product staff, not editors

•One day there will be no print so yes it’s a must

•Publishers must be current in their field of work

•I believe that this is context dependent and journal dependent and is not necessarily a skill required by all editors

•Knowing what is happening in tablet development has no influence on my work as an editor

•I would say very important for publishing, less so for scientific editing.

•This is insufficiently emphasized and it is the publishers role to actively teach editors on this.

•Perhaps this is more for the publisher but it will impact on its relation to the EIC

•same as 72

•Most journals are not even close to these advances in electronic media

74. Engage in multimedia publishing practices

•Engage in multimedia publishing practices

•Essential in today´s world.

•Score lower as this is a matter of choice and of resources

•same as 72

77. Demonstrate knowledge of open access models

•Editor in chief may not even need to know this

•Publishers must be current in their field of work

•This is up to the legal staff

78. Demonstrate knowledge of issues related to predatory publishing

•Editor in chief may not even need to know this

•Publishers must be current in their field of work

•I don't think this is a core competency

•This is up to the legal staff

•More emphasis should be on this.

•While interesting, I do not see how this enhances the ability of an editor who does not run a predatory publication

79. Demonstrate understanding of Creative Commons licensing

•Since CC is replacing copyright in especially open access journals, this is important

•Editor in chief may need to know this

•Publishers must be current in their field of work

•This is up to the legal staff

•Happy to leave this to management

81. Demonstrate knowledge of history of journals and scientific publications

•I put a high value on being aware of history - it adds balance to all decisions.

•Knowing the history of journals and scientific publications facilitates a conscious and committed work

•I don't think this is such a priority for editors, except the very basics

82. Demonstrate knowledge of national and regional variations between journals

•Editor in chief may need to know this

•To better target the authors about the suitability of the journal to which to send their paper

•Not a major issue

•Only if working across a range of journals: not for editors working on a single journal, hence not a core competency

•I am not sure that this segregation is appropriate, all journals strive for the same - to present good and important research

83. Demonstrate knowledge of political and geopolitical issues

•Health care is a problem of all populations in the world. Ours is an international journal, published in the US. Given that many of our readers are from developing world I need to aware of some issues in maternal-child health, for instance, that we need to address.

•To better target the authors about the suitability of the journal to which to send their paper

•Not a major issue

•How can editor demonstrate this knowledge, and how it is so relevant for the editorial work

An editor is responsible to distribute produced knowledge to different audiences including policy makers and politicians. Without knowledge in these issues it is not possible for editors to fulfill these responsibilities

84. Demonstrate familiarity with associations and their educational resources

•Editor in chief may not even need to know this

•To work together for the continued education of potential authors

88. Ensure controversial topics (political, ethical) are dealt with

•I put a high value on serving the community and this is a key way to do so.

•To ensure that the journal freely try the current issues

•Unclear what is meant by 'dealt’

•I don't really see the point of this question as it is not clear whether this is about controversial topics in the society or in editing and publishing?

89. Stimulate others to write articles and editorials

•To promote the journal among the critical mass

•This is great when it happens, but I wouldn't say a core competency.

•Required for keeping the journal´s workings transparent and wide-ranging.

•89- to 92 are all key roles in which the EIC can have a substantial impact

91. Entice leading researchers to submit to the journal

•To promote the journal among the critical mass

•it's the same question of what appears on Question 5 and has to do with the creation of a network of authorship. In my opinion it is a core competence

•This is great when it happens, but I wouldn't say a core competency.

89- to 92 are all key roles in which the EIC can have a substantial impact

93. Motivate physicians to read, ponder, and implement the information provided

•I do not necessarily disagree with the rating but my earlier lower rating was based on the fact that this seemed very much focused on clinical evidence, I viewed the role as broader and covering also non-clinical research which may or may not be relevant to promote among physicians for immediate application.

•This is already ranked above the middle but for my journal, which is a clinical journal, if the doc's aren't reading this I'm not meeting my goals. If there are ways to make our information more accessible or digestible, I need to be finding them.

•To promote the journal among the critical mass

•Physicians not a relevant audience to many journals. Leaving that aside and assuming whatever readership, the competency would be more like ability to select interesting papers and encourage papers to include implementable messages?

•I think that this is important

94. Seek feedback/opinions on the journal

•To demonstrate the necessary opening leading to improving the quality of the journal

•Experience over decades tells me that feedback/opinions are so individual they help little. Metrics are more valuable.

•Receiving feedback is the first step in improving the journal.

•I think that this is important

95. Enhance public understanding of science

•It's not the responsibility of all editors. It depends on the journal policy.

•Not a primary responsibility of a journal editor.

•To ensure that the communication process is completed

•This is for journalists, not editors

•This is great when it happens, but I wouldn't say a core competency. Public communication is not everyone's skill set and doesn't have to be

•A role for institutions and academia generally more than editors

•I would stay with my original score, as we publish health research, where the involvement of patients is crucial and they need to be better informed about what we publish

•I think that this is important

96. Demonstrate understanding of who one’s constituency is

•'Constituency' is not a helpful word here. Journal audience is covered by other competencies listed

This is managed by Universiti Sains Malaysia Press official of the journal section

98. Hold paramount the interests of the particular journal’s readers

•It's not the responsibility of all editors. It depends on the journal policy

•Target audience of the journal

•Journals are not only an element to appear in indexes or databases, journas must be read. For me is a core competence attract readers, and they read it.

• ‘Paramount' suggests more important than anything else. Unclear what this means in practice. Doesn't seem like a specific competency

99. Engage in communication with the public

•Editor in chief may need to do this

•To attract potential readers

•This is not a priority

•More journalism than editing

•The public were rarely my readers

•To me editor is not only responsible for making decisions on the manuscripts but also to distribute knowledge to different audiences.

100. Engage with existing and new scientific communities

•To attract potential readers

•To me editor is not only responsible for making decisions on the manuscripts but also to distribute knowledge to different audiences.

101. Engage with social media to reach out beyond the usual specialist audiences

•To attract potential readers

•Social media outreach important for journals but not a core competency for scientific editors.

•" - not yet convinced of value of social media - probably I am too old

•This is managed by Universiti Sains Malaysia Press official of the journal section

•To me editor is not only responsible for making decisions on the manuscripts but also to distribute knowledge to different audiences.

102. Demonstrate proficiency with writing news releases

•Should be able to check accuracy as well as appropriate style for lay audience

•I would give this a lower rating, I do not see it as critical for an editor to handle the writing of press releases, there are science/medical writers who can handle that

•News releases are not usually the responsibility of most scientific editors

•Not the responsibility of a journal editor. This is the marketing/promotional people's responsibility, and something that they should have training and experience of. We would not, for example, expect THEM to be able to decide on a manuscript..?!

•In order to increase the visibility of research results should be widely disseminated

•May be done by others, not a core competency for scientific editors. Perhaps could be identifying potentially newsworthy papers and working with communications teams?

•I can see why others have not thought this important, but it is a critical part of communicating scientific research to a lay audience. News releases from journals are very often poorly written and exaggerate key findings in all sorts of different ways (e.g. 'Intervention reduces death by 60%' versus 'Intervention leads to 2 fewer deaths per thousand people treated'). If you can integrate the development of a press release with an internal quality assurance check then this ideal: you have within your grasp the means to understand the research, spot (and fix) inconsistencies/common errors before bringing what you have learnt about the research in to the press release. You need to work with press officers to do this effectively so having a good relationship with your marketing team is important. All editors should at some point work on a press release to gain vital experience in minimizing spin in the reporting of scientific research.

•This is frequently part of the role and it is a useful skill for promotion

•This is managed by Universiti Sains Malaysia Press official of the journal section

•To me editor is not only responsible for making decisions on the manuscripts but also to distribute knowledge to different audiences.

105. Demonstrate knowledge of typography

•Most scientific editors will never need to know anything about typography

•Not an editor's responsibility, and not their decision either. This would be decided by the journal owner and publisher.

•It is part of the knowledge of editorial process

•Understand

•Not a core competency for scientific editors.

•Depending on much obsessive-compulsive behavior one needs to input into the process...

•Perhaps more an issue for copy editors / publishers

•This is managed by Universiti Sains Malaysia Press official of the journal section

106. Demonstrate knowledge of and experience with online editing

•Content editor is not equal to copy/technical editor.

•The editor should be updated and in some journals online publication is usual

•This is a core skill of editors.

•This is managed by Universiti Sains Malaysia Press official of the journal section

107. Demonstrate knowledge of the fundamentals of editing various types of manuscripts

•I am not sure that I understand the question.

•Still a bit low - these are bread and butter skills

•It is part of the job of editor

•Editors should be able to demonstrate general editorial ability, even if their day-to-day work involves editing limited article types.

•Not clear enough - what are the 'fundamentals of editing'?

•This is managed by Universiti Sains Malaysia Press official of the journal section

111. Ensure papers selected are clinically relevant

•I would give this a lower rating because many journals have scopes that go beyond clinical work and thus may not select on basis of clinical relevance

•Crucial for an editor

•To ensure the relevance and timeliness of the information published

•Not all medical papers are clinically relevant. Some are the scientific basis of future clinical work

•In fact, this seems the major role for a scientific editor

•Clinical relevance not relevant to all journals. Could reframe as ensuring a clinical relevance is articulated meaningfully where appropriate?

112. Ensure papers selected have a clear story-line

•Up a bit (although I think this is a hall mark of a good editor)

•To ensure better understanding of the message

•These are very useful

122. Engage in, and maintain interactions and good relations with media

•For better visibility of the journal

•Many publishers have a press team for this.

•Great when it happens, but not a core competency of scientific editors. PR is not everyone's skills set and doesn't have to be.

•This has to be done with and through the publisher and a press officer there.

•My original score was reflecting my opinion that informing the public about health research is important

•This is managed by Universiti Sains Malaysia Press official of the journal section

123. Select, curate, and comment on articles for publication

•Why know how to curate

•It is part of the work of the editorial office

•This is managed by Universiti Sains Malaysia Press official of the journal section

125. Demonstrate experience or familiarity with manuscript tracking software (e.g. ScholarOne, AllenTrack, PeerTrack, BenchPress)

•Today, I am not sure how an editor could do the job without this knowledge.

•You need to know what the authors contend with

•The editor should be updated

•I believe that this is context dependent and journal dependent and is not necessarily a skill required by all editors

•This is not a major task for a scientific editor, other people can help

•This is quite essential. And these interfaces can be glitchy and cumbersome

•This is managed by Universiti Sains Malaysia Press official of the journal section

•I think that this is important I will keep

•As prior experience, I don't think this is necessary.

126. Demonstrate aptitude in using technology (computers, Internet, e-mail, Manuscript Submission Systems) to perform his or her editorial duties

•Today, I am not sure how an editor could do the job without this knowledge.

•The editor should be updated

•This is not a major task for a scientific editor, other people can help

•Again this is a core skill of editors. Publishers need to use these technologies for efficient production.

•This is managed by Universiti Sains Malaysia Press official of the journal section

127. Possess a degree in medical editing or be trained as a journal editor

•I rated this higher as I interpreted this to mean that for new editors who lack a degree in medical editing, there should be some training or on boarding. I don't believe that new editors must be formally trained or trained as an apprentice. If that was the intent, I agree with this rating.

•I do not subscribe to the idea that researchers can be autodidactic about everything. Some training/mentorship is required.

•Being trained would be a plus

•Editors should receive formal education and deal with their continuing education

•No understand the process

•I believe specific editorial training is required, and having experience of being an author/academic is not sufficient training to become an editor.

•Purpose of training is to achieve competencies, not a competency in itself.

•Not essential

128. Demonstrate the ability to write editorials

•The word is "EDITORial" - who else should write them?

•Sometimes the editor writes editorials

•Many editorial staff never write editorials

•I have written many but there are so many potential writers, the editor can easily take a back seat

•Depending on the journal it is an important skill

130. Demonstrate skills in speed reading, skim reading, and critical reading

•These are skills and abilities must have the editor

•Three different skills.

•Yes to critical reading

•Don't think speed reading or skim reading are required by all editors - may be useful in high-volume journals for

•Those involved in triage but useless to others.

•I would retain my original score as I am not convinced that speed and skim reading are important, critical reading yes

•I wonder without this how you can define an editor

131. Demonstrate an aptitude for reading widely, deeply, and continually

•This is commonly known as scholarly activity. Can one be a journal editor without it?

•These are skills and abilities must have the editor

•I wonder without this how you can define an editor

132. Demonstrate experience and/or training in medical journal writing

•The editor should be updated

•As for Q128, many editors don't write

•Same as 127: I believe specific editorial training is required, and having experience of being an author/academic is not sufficient training to become an editor.

134. Demonstrate familiarity with scientific units, numerals, symbols and nomenclature

•This is a scientific editor you are dealing with here

•These are skills and abilities must have the editor

•A scientific editor ought to be familiar with scientific units etc., so this should be a 5 or very close to it.

137. Demonstrate knowledge of literature reviews

•I guess it may depend on what is implied by ‘knowledge of’, not all journals publish literature reviews so it will not be critical for all editors to have familiarity handling this, given this I would give this a rating in the middle of the range.

•Surely fundamental - editors have to know what these are about. They are ubiquitous

•These are skills and abilities must have the editor of scientific publications

•Cannot be expert at everything so must rely on specialist reviewers for this

143. Be working towards a deeper understanding of multiple research epistemologies

•This question is inferred in other questions

•At least - growing area

•These are skills and abilities must have the editor of scientific publications

•Actually a weasly question as we can all agree we should be 'working towards' most things.

144. Demonstrate knowledge of issues related to supplemental materials for manuscripts

•At least - increasingly common

•These are skills and abilities must have the editor of scientific publications

•If the journal published supplementary materials, the editor must know about any issues related to them.

•Rely on technical and copy editors for this

•These have to be viewed critically

145. Demonstrate the ability to work with editors of other journals

•These are skills and abilities must have the editor of scientific publications

•Rarely a need to do this

•This is managed by Universiti Sains Malaysia Press official of the journal section

146. Demonstrate knowledge of issues related to open data

•At least - growing importance

•These are skills and abilities must have the editor of scientific publications

•This is managed by Universiti Sains Malaysia Press official of the journal section

147. Demonstrate familiarity with referencing software

•These are skills and abilities must have the editor of scientific publications

This is managed by Universiti Sains Malaysia Press official of the journal section

148. Demonstrate knowledge of Digital Object Identifier assignment

•DOI is important for archiving

•This is a task for production editors or publishing staff, not scientific editors

•Important if the journal cares about citations

•These are skills and abilities must have the editor of scientific publications

•Digital Object Identifier. Why half so low ?. It is one of our elements citation.

•Part of today´s world.

•This is managed by Universiti Sains Malaysia Press official of the journal section

160. Demonstrate understanding of issues related to dual-use research (research with multiple purposes or applications)

•These are skills and abilities must have the editor of scientific publications

•This is a relevant commitment for a scientific editor

162. Guarantee access to, and long term preservation of, the published information

•This includes DOI rather than relying on non-permanent URLs

•I see it as part of the editors’ duty to contribute to both the access and stability of the published record, this involved preservation of the information.

•This is a publisher task

•Not the responsibility of the editor. This is the responsibility of the journal owner/publisher.

•Relevant for an editor

•These are skills and abilities must have the editor of scientific publications

•This is a publisher role, not an editorial one

•This should be the role of the publisher.

•I would say this is a publisher, policy and technological issue rather than a core competency for scientific editors.

•I would stay with my original rating, because this is the basic standard of scientific publishing

163. Encourage debate on important topics related to the journal

•To ensure adherence to the aims of the journal

164. Promote higher standards of medical journalism

•To ensure adherence to the aims of the journal

167. Demonstrate knowledge of copyright issues

•Still pretty key - you don't have to know it all

•To ensure the quality of the journal

•This is a legal issue, other people can manage it

•Potential breached of copyright in a paper should be identified by the scientific editors.

•You can no deal with plagiarism unless you have awareness in this field

171. Identify and address issues related to image manipulation

•One of the major reasons for paper retraction or withdrawal is image manipulation. These will come up and we all need to be prepared to deal with them.

•Editor in chief may not even need to know this

•To ensure the quality of the journal

•Essential! Too much bad imagery and author-made alterations

•I think this is too specific for editor

176. Effectively summarize manuscripts in fields outside your experience

•That is a skill that must have a scientific editor

177. Possess a Doctorate or Master's Degree in related content area

•If the point is widened to "possess a post-graduate qualification" to take into account MDs. It is difficult to be an editor with only an undergraduate degree.

•Knowledge not letters- see 178

•This is desirable in a scientific editor

•Editors do not necessarily all need to be content experts. Content expertise can be sought from peer-reviewers and other content experts at the journal. Scientific editors have different skills to content experts (and it is not copy editing).

•Not a competency. Qualifications are ways to demonstrate that you have competency (e.g. in academic writing, a clinical field or in editing)

•One must oneself have very good research experience.

•I have met many competent editors who do not. EICs have to be active in their field

179. Demonstrate experience and aptitude in conflict resolution

•This is a feature of teamwork

•This is an important issue for editors

•I think that this is important

181. Maintain part time professional practice

•I do not subscribe to the model of an editor being long inactive from the field.

•One should not depend on journal for a living. Can create a bias if acceptance of more articles means more monetary benefit for the editor

•Is necessary to seek the integration of teaching, research and professional practice in a particular area of knowledge, and in this case, the scientific publishing

•As a successful editor who has never been in medical practice, I do not see this as core

•As above, not all editors need to be content experts. The team should comprise content experts and 'professional' editors whose primary role is to edit manuscripts.

•Does this refer to clinical practice? Not relevant to all editors. Research practice may not be practical on a part-time basis, and some editors are full-time.

•Related to the requirement that editors have extensive knowledge of the field)

•I think that this is important

182. Maintain membership in learned societies and editing-related associations

•I do not subscribe to the model of an editor being long inactive from the field.

•Because they are our peers

•May be important, but not a competency (not hard to fill in the membership form and pay the fee!

•This is very important to stay in contact with the professional community these organizations are the best places to provide training for editors

183. Be recognized as a distinguished scholar in one's field

•I do not subscribe to the model of an editor being long inactive from the field

•The distinction itself is not important, but knowing part of a collegial group

•One needs to be recognized as competent and knowledgeable but not as a scholar

•As above, not all editors need to be content experts.

•Not a competency, and unreasonable to expect that all the editors of the world be 'distinguished scholars', although great to have a few on your team.

•Helps the journal.

•Although this may be essential for very specialist areas it is not essential at all for more general journals.

•This is an important quality of a good editor

184. Maintain an active research portfolio/ is employed in a research-oriented university or institute

•This will depend on the type of editorial role, editors who work full time for a journal/publisher may not be able to maintain active research activities (beyond those run by the journal itself).

•I do not subscribe to the model of an editor being long inactive from the field.

•An editor unaware of latest trends of research will lose the race

•Because the editor is part of a scientific community

•Does help for contact for potential articles

•Similar to Q181 Not necessary

•As above, not all editors need to be content experts.

•Not practical for full-time editors or health professionals.

•See 183

•Debatable as many journals are going on with the professional EIC model employing a person no longer involved in academic research. This still has enormous value depending on the journal.

•This is very important for editors of small and scholarly journals

•This is necessary to be up-to-date even though time constraints does not allow. I think answer to this item is biased by the current situation of responders.

185. Demonstrate past experience on an editorial board

•If this were required, there would never be any new editors

•Historically the editor is formed in editorial work and then seek formal education and continuing education

•Not necessary. Many good editors have not sat on boards

•Experience is a way of demonstrating competence, not a competence per se.

•An ambiguous question as 'editorial boards' differ so much. Some are just lists of great and good so the experience is of little value. Others are active in the editorial process so are.

•I think this is an insurance for quality leadership in the long run

186. Demonstrates competence as a practitioner in his or her field

•I do not subscribe to the model of an editor being long inactive from the field.

•The editor is specialized in a certain field of knowledge and he is done editor through the practice and formal education and continuing education

•Unless you mean the field of editing/publishing, I do not see this as a requirement

•As above, not all editors need to be content experts.

187. Demonstrate strong interpersonal skills

•Editors handle public relations with authors, reviewers, decision makers, the mass media, therefore must have interpersonal skills

190. Demonstrate the ability to achieve consensus among opinionated scientists

•Editors handle public relations with authors, reviewers, decision makers, the mass media, therefore must have interpersonal skills

•Scientific consensus probably an impossible goal, like world peace. Good interpersonal and negotiation skills probably useful.

191. Demonstrate leadership skills

•Editors handle public relations with authors, reviewers, decision makers, the mass media, therefore must have interpersonal skills

•Not all editors are leaders: some are team members

192. Demonstrate political and public relations sense

•Editors handle public relations with authors, reviewers, decision makers, the mass media, therefore must have interpersonal skills

•Not relevant to many editors, hence not core

194. Demonstrate enthusiasm

•Editors handle public relations with authors, reviewers, decision makers, the mass media, so they must show leadership

•Great, but don't think it's really a core competency

196. Demonstrate boldness

•Editors handle public relations with authors, reviewers, decision makers, the mass media, so they must show leadership

•Many editors can follow, not needing boldness

•Too vague. Prefer the wording at no.197 on independent thinking.

•Boldness can be destructive as it may lead to poor judgment

198. Maintain visibility and respect among peers and in the larger scientific community

•Editors handle public relations with authors, reviewers, decision makers, the mass media, therefore must have interpersonal skills and leadership

•Great when it happens, but don't think this is a core competency for all scientific editors.

199. Maintain rigid criteria

•About what?

•Disagree that non-specified 'rigid criteria' are a core competency. Some rigid criteria appropriate, others not.

•I do not understand the question but distrust all rigidity except in an aircraft fuselage

•Some flexibility is sometimes necessary

•This item is not clear, as well as the fact that mostly each case is different and requires an individual approach; nothing is black and white but rather a scale of grayness

200. Demonstrate the ability to perpetuate or challenge master narratives

•The inquisitiveness and creativity are characteristics of editors

•Unclear what this means. 'Perpetuate or challenge'?

•This statement is not clear in its meaning (at least to non-English persons) and relevance to editors

201. Exercise convictions with a positive attitude

•It is necessary for a good editorial work

•Acting on convictions and positive attitude are two different things

•Don't understand this management speak

•A positive attitude always helps to solve difficult issues

•It is not clear what is exactly meant here for the editors

203. Demonstrate practicality

•It is necessary for a good editorial work

•What is meant by "practicality" in editorial setting?

205. Demonstrate personal interest in medical “journalology” or “editology”

•Wow. Can't imagine doing this work without an interest in the process and the end products. I would be bored and

•More likely to miss deadlines. This is a must!

•As part of the comprehensive work on the figure of health professional and scientific editor

•Understanding of, yes, 'personal' interest, no.

207. Demonstrate the desire to advance his or her field of study

•As part of the comprehensive work on the figure of health professional and scientific editor

208. Have access to a good academic network or have the potential to grow one

•As part of the comprehensive work on the figure of health professional and scientific editor

Agood academic network can contribute to support editorial work

213. Demonstrate managerial skills

•The editor has to set the agenda and keep the ball rolling. Depending on the size of the staff, if any, and the complexity of the reviewing and editing process, there are a lot of concurrent processes happening which are people dependent. The Editor has to be able to manage this process.

•It is a feature that should have every publisher

•Managerial skills are very important to cope with the different aspects and responsibilities of the editorial work

214. Demonstrate the ability to link journal content to current affairs

•It is a feature that should have every publisher

•I don't see this as an essential role of an editor

•OK for a weekly journal but of little value for others

**Items Added from Round 1 and Rated in Round 2**

A. Able to assess appropriateness of research methods in relation to the stated research question

B. Able to assess validity of conclusions in relation to the research findings

C. Understanding of issues relating to reproducibility of research

D. Understanding of issues relating to selective outcome reporting

E. Ability to detect conflicts that have not reported by authors, and deal with these appropriately

F. Having expertise in how to correct the published record

G. An understanding of different peer review models to improve efficiency of reviewing processes, such as cascading

H. Understand impact factor and how citations can help with impact factor

I. Good command of Scientific English

J. Engage with copy editors

K. Ability and willingness to arrange regular editorial meetings to discuss matters arising

L. Ability to check that you are making consistent and transparent decisions

M. Involve patients, research subjects and communities in journal policy

N. Awareness of cultural differences

O. Ability to handover his/her tasks and duties to the next scientific editor

P. Language competency particularly from a country where English is not the native-tongue

**Delphi Round 3 Text Responses**

This is a list of the items from Round 3 of the Delphi. These items did not achieve at least 80% consensus for inclusion, thus participants rated them one last time to indicate whether they judged them to be: 1) Not important, 2)Important but not essential, or 3) Absolutely Essential. Participants were asked to invited to include comments to support their answers.
