# Supplementary material for: An international survey and modified Delphi process revealed editors’ perceptions, training needs, and ratings of competency-related statements for the development of core competencies for scientific editors of biomedical journals
Source: F1000Res. 2017 Sep 4;6:1634. [Version 1] doi: 10.12688/f1000research.12400.1 (PMC5605946; doi:10.12688/f1000research.12400.1)
Supplement: Peer review history

# Peer review: Referee response for version 1

**DOI:** 10.5256/f1000research.13429.r25638

**Published:** 2017-09-15

**Authors:** Chris Graf

**Affiliations:** 1 John Wiley & Sons Ltd., Oxford, UK

I have no recommendations to the authors that must be addressed. This report is part of a larger study, to identify core competencies for biomedical journal editors. The methods seem appropriate, and are well described. The results are presented clearly. The supplemental materials provide the materials (survey questions) and data. The conclusions are apt.

I have read this submission. I believe that I have an appropriate level of expertise to confirm that it is of an acceptable scientific standard.

# Peer review: Referee response for version 1

**DOI:** 10.5256/f1000research.13429.r25637

**Published:** 2017-09-12

**Authors:** Thomas A. Lang

**Affiliations:** 1 Tom Lang Communications and Training International, Kirkland, WA, USA

The authors surveyed 150 editors for their opinions of the knowledge and skills needed to edit scientific journals. In a modified Delphi process, the editors then rated the importance of dozens of competencies identified in a previous scoping review to create a list of minimum competencies to be addressed in editor-training programs.

 The survey and development processes seem reasonable and straightforward, and the results have high face validity, raise no concerns, and do not appear to be controversial. The authors accomplished their objective, which was to create an evidence-based list of core competencies that can form the basis for training editors of biomedical journals.

 The wording of the competency statements is not always parallel or specific, but the thrust of each is clear enough that reasonable people could develop each further.

I have read this submission. I believe that I have an appropriate level of expertise to confirm that it is of an acceptable scientific standard.
